# Multi-omic mapping of *Drosophila* protein secretomes reveals tissue-specific origins and inter-organ trafficking

**DOI:** 10.1038/s41467-026-71763-8

**Published:** 2026-04-20

**Authors:** Justin A. Bosch, Pierre Michel Jean Beltran, Cooper Cavers, James Thai LaGraff, Randy Melanson, Ankita Singh, Weihang Chen, Yanhui Hu, Sudhir Gopal Tattikota, Ying Liu, Yousuf Hashmi, John M. Asara, Tess Branon, Alice Y. Ting, Steven A. Carr, Norbert Perrimon

**Affiliations:** 1 Department of Genetics, Blavatnik Institute, Harvard Medical School, Boston, MA USA; 2 Broad Institute of MIT and Harvard, Cambridge, MA USA; 3 Division of Signal Transduction, Beth Israel Deaconess Medical Center, Boston, MA USA; 4 Department of Medicine, Harvard Medical School, Boston, MA USA; 5 Departments of Genetics, Biology, and by courtesy, Chemistry, Stanford University, Stanford, CA USA; 6 Department of Chemistry, Massachusetts Institute of Technology, Cambridge, MA USA; 7 Chan Zuckerberg Biohub-San Francisco, San Francisco, CA USA; 8 Howard Hughes Medical Institute, Boston, MA USA; 9 Present Address: Department of Human Genetics, University of Utah School of Medicine, Salt Lake City, UT USA

**Keywords:** Blood proteins, Proteomic analysis, Secretion, Drosophila

## Abstract

Secreted proteins regulate many aspects of animal biology and are attractive targets for biomarkers and therapeutics. However, comprehensively identifying the “secretome”, along with their tissues of origin, remains extremely challenging. To address this, we employed multiple ‘omics methods to define a tissue-secretome map of 535 blood plasma proteins derived from specific cell-types and organs in *Drosophila melanogaster*. This map was enabled by methodological improvements including a collection of transgenic flies to label endogenous secreted proteins in 10 major tissue types, large-scale blood isolation, whole animal snRNA-seq, and 40 CRISPR knock-in strains. Using this map, we identify features of circulating proteins: most originate from specific tissues including unusual sources (e.g. glia), many are uncharacterized, and some are shed ectodomains of transmembrane proteins. In addition, in vivo experiments revealed circulating proteins with tissue-specific expression, as well as proteins that are deposited in a different tissue from where they are synthesized, suggesting potential inter-organ functions. Our secretome map will serve as a resource to investigate blood protein function, discover candidate tissue-tissue communication signals, and mine for homologues of human biomarkers.

## Abstract (web summary)

A *Drosophila* secretome atlas identifies the tissue origins of circulating proteins, revealing unusual sources, uncharacterized proteins, ectodomain shedding, and candidate mediators of inter-organ communication.

## Introduction

Secreted proteins play essential roles in development, immunity, and metabolism by acting in the extracellular space to influence nearby or distant cells. Additionally, disruptions in secreted protein signaling are implicated in a wide range of diseases, including diabetes and cancer. Because they are more accessible than intracellular proteins, secreted proteins and their potential cell surface receptors are attractive targets for both therapeutics and biomarkers. While efforts to define the full set of secreted proteins (the “secretome”) have yielded thousands of candidates^1,2^, we still lack a comprehensive map of their cell-type-specific sources, extracellular distribution (local versus circulating), and non-autonomous functions. Such a detailed map of secreted proteins would enhance our understanding of basic biological processes and disease mechanisms, and lead to improved therapeutics and biomarkers.

Mapping secreted proteins remains technically challenging. For example, mass spectrometry (MS) of extracellular fluids can identify secreted proteins but not their source cells^3^. While the extraction of extracellular fluids from specific tissues can be performed before MS^4^, it is not adaptable to all cell types. Furthermore, combining MS with RNA-seq has been used to map candidate tissue sources^2,5^, but this approach does not physically trace the secreted protein back to the tissue of origin and cannot distinguish constitutive secretion from cell lysis or regulated secretion. Additional challenges of MS are that secreted proteins are obscured by abundant proteins or misattributed to the target tissues they accumulate in, rather than those that produce them. These issues highlight the need for new tools, especially those that directly trace and enrich secreted proteins from complex biological samples.

Proximity labeling (PL) enzymes have recently emerged as a promising solution. Promiscuous biotin ligases like BioID and TurboID can label endogenous proteins in living cells with biotin^6,7^, and biotinylated proteins can be isolated using streptavidin beads. Thus, biotinylated proteins can be enriched from specific cell populations in complex biological samples, reducing background. When targeted to the secretory pathway, these enzymes can label endogenous secreted proteins, which are then recovered from the extracellular space. Several studies have used promiscuous biotin ligases to identify cell/tissue-specific secretomes^8–16^ as well as proteins trafficking between cells and organs^17,18^. However, these studies are limited to a few cell types or organs, and broader efforts are needed to approach a full tissue-level secretome map. Furthermore, while many of these studies utilize mouse models, systematic in vivo characterization of candidate secreted proteins remains challenging due to the time, cost, and complexity of functional studies in mammals.

*Drosophila* is well-suited for this task due to its conserved physiology and powerful genetic tools. For example, flies have conserved organ systems with mammals, including the brain, muscle, and gut. Furthermore, its blood (or hemolymph) is analogous to human plasma, containing human-conserved circulating proteins that control immunity, coagulation, and systemic physiology^19–21^. These circulating proteins include “inter-organ factors”, defined here as proteins synthesized in one tissue and transported through the circulation to function in another. These include classical signaling ligands (e.g., hormones), nutrient transporters, and extracellular matrix factors. *Drosophila* genetic tools also allow rapid in vivo characterization of candidate genes at large-scale, facilitated by an enormous collection of tissue-specific Gal4 driver transgenes^22–24^. Importantly, tissue-specific expression of proximity labeling enzymes in *Drosophila* is non-toxic^6,18,25,26^. However, its small volume of blood (or hemolymph) has prevented a large-scale, unbiased secretome analysis. Therefore, improved protocols and tools are required.

Here, we employed TurboID proximity labeling, mass spectrometry, single-nucleus RNA-seq (snRNA-seq), and CRISPR gene editing to create a comprehensive secretome map in *Drosophila* third instar larvae. We identified 535 circulating secreted proteins and their tissues of origin, revealing the relative contributions of different tissues to blood proteins, uncovering uncharacterized tissue-specific secreted proteins, and identifying candidate mediators of inter-organ communication. This work establishes a scalable platform for secretome discovery in *Drosophila* and opens the door to systematic exploration of inter-tissue signaling.

## Results

### Development and optimization of an in vivo tissue-specific TurboID secretome-labeling platform in *Drosophila*

**Fig. 1:**
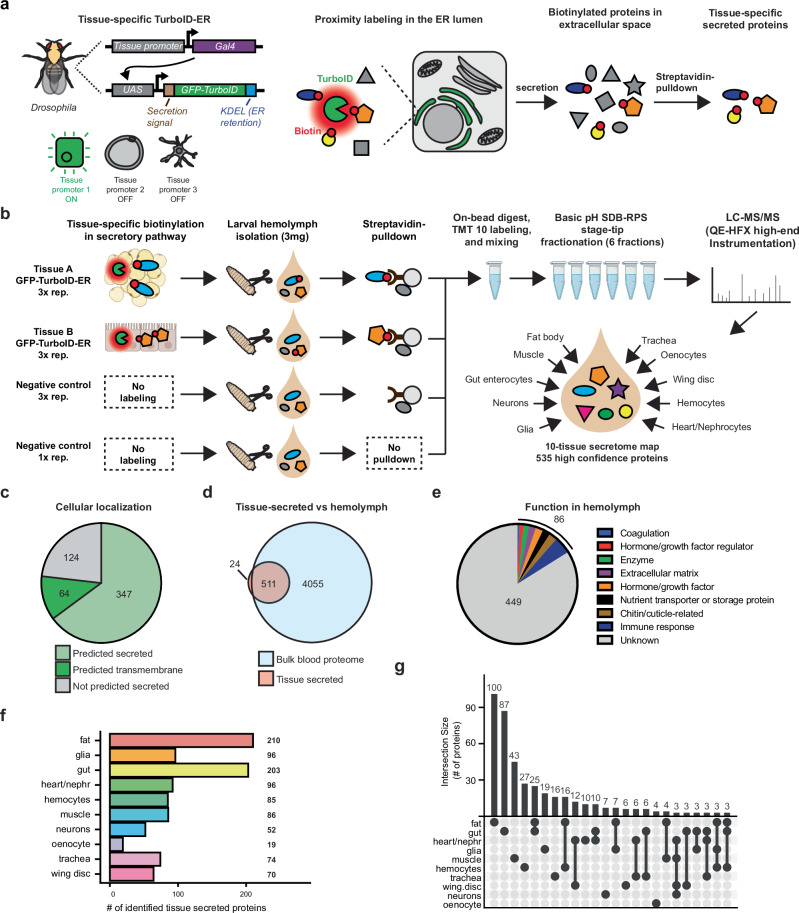
A secretome map of 10 major *Drosophila* larval tissues using quantitative proteomics. **a** Schematic of genetic cell-type-specific expression of GFP-TurboID-ER with the Gal4/UAS system, labeling of endogenous proteins in the ER-lumen, followed by purification of biotinylated proteins from the extracellular space. **b** Workflow for proteomic analysis of tissue secreted proteins. Created in BioRender. Bosch, J. (2026) https://BioRender.com/nr7nmts. **c** Pie chart of the predicted cellular localization of 535 tissue-secreted proteins. The number of proteins in each category is indicated. **d** Venn diagram of the overlap between proteins identified using tissue-labeling vs. whole blood. **e** Pie chart of known hemolymph functions of the 535 tissue-secreted proteins. **f** Bar graph showing the number of proteins identified as secreted from each tissue with a high-confidence criterion. **g** Upset plot showing the number of proteins identified as secreted by a unique tissue or by multiple tissues. Intersects with >3 proteins are not shown.

To enable identification of tissue-secreted proteins in *Drosophila*, we sought to use an in vivo proximity labeling approach. Like other studies, we wanted to genetically express a promiscuous biotin ligase (TurboID in our case) that is targeted to the endoplasmic reticulum (ER) lumen to label endogenous secreted proteins^11–15,18^, which are then purified from the extracellular space (Fig. 1a). To test our approach, we generated plasmids expressing GFP-TurboID-ER and transfected them into cultured S2R+ cells^27^ (Supplementary Note 1). We found that GFP-TurboID-ER localized to the ER lumen in S2R+ cells, selectively biotinylated proteins in the secretory pathway, and enabled recovery of labeled secreted proteins from culture media, as validated by microscopy, western blotting, and liquid chromatography–tandem mass spectrometry (LC–MS/MS) (Supplementary Figs. 1–5; Supplementary Data 1). Next, we tested labeling secreted proteins in vivo by generating a panel of transgenic flies expressing GFP-TurboID-ER in 10 major tissue types using the Gal4/UAS system^28^ and detecting biotinylated proteins in 3rd instar hemolymph (i.e., blood) (Supplementary Note 2). Our results confirmed robust labeling of secreted proteins, with distinct tissue-specific patterns and strong dominance of fat body-derived proteins, without detectable phenotypic or viability defects (Supplementary Fig. 6). To support large-scale streptavidin pulldown and quantitative LC–MS/MS, such as milligram-scale input material in triplicate, we developed a new hemolymph isolation protocol (Supplementary Note 3). Pilot LC–MS/MS experiments showed efficient enrichment of secreted and tissue-derived proteins, including known hemolymph components and uncharacterized factors, but also revealed non-specific binding of abundant hemolymph proteins to streptavidin beads (Supplementary Fig. 7, Supplementary Data 2). To address this, we developed optimal streptavidin bead-washing conditions that reduced non-specific binding without compromising recovery of biotinylated proteins (Supplementary Note 4, Supplementary Fig. 8). In summary, these reagents and protocols establish a robust and scalable workflow suitable for large-scale secretome profiling in *Drosophila*.

### A quantitative proteomic secretome map of 10 major larval tissue types

Building on our pilot LC–MS/MS results in cultured cells and larvae, we created a comprehensive tissue secretome resource from *Drosophila* larval blood, using quantitative proteomics to profile proteins secreted from 10 tissues. Biotin-labeled proteins were enriched from the blood using our improved bead-washing conditions in triplicate, released from streptavidin beads by on-bead digestion, and analyzed by LC–MS/MS employing isobaric chemical labeling using TMT reagents for precise relative quantification (Fig. 1b). A total of 2543 proteins were quantified across the ten experimental groups and fold changes were calculated against enrichments from negative control pulldown samples (Supplementary Data 3a, Supplementary Fig. 9a). The experimentally enriched proteins showed overrepresentation of proteins containing signal peptide sequences (Supplementary Fig. 9b), and principal component analysis showed clear separation of experimental groups from each other and from the control sample (Supplementary Fig. 9c). To confidently identify candidate secreted proteins, we established fold-change thresholds for each tissue by minimizing nominal false discovery rates (FDR) using a predefined list of positive-control secreted proteins and negative-control intracellular protein lists (Supplementary Data 3a, Supplementary Fig. 9d). Using these strict thresholds, we report 535 proteins as candidate secreted factors from at least one of these 10 tissues (Supplementary Data 3b). We also report 31 proteins detected in experimental replicates but with zero values in all negative controls, preventing calculation of fold-change and FDR. Therefore, they were conservatively excluded from the high confidence set (Supplementary Data 3c, see the “Methods” section).

First, we examined what proportion of the high-confidence 535 proteins are known or predicted to be secreted. Using a combination of protein databases and domain prediction algorithms, we determined that 411 (77%) have the potential to be secreted or transmembrane (Fig. 1c, Supplementary Data 3b). In addition, 267/535 (50%) of proteins are annotated as in hemolymph (Flybase, Uniprot) or present in a previous 3rd instar larval hemolymph dataset^20^ (Supplementary Data 3b). Finally, we generated a label-free 3rd instar hemolymph proteome of 4566 proteins using extensive fractionation for deep-scale analysis (Supplementary Data 3d), in which 511 (96%) of our high-confidence proteins were present (Fig. 1d). In total, 513 (96%) of our high-confidence tissue-secreted proteins are present in at least one hemolymph dataset (Supplementary Data 3b). This label-free data also highlights the advantages of TurboID-ER labeling, namely tissue-of-origin information, enrichment relative to abundant proteins, and limited to classically secreted proteins. For example, the three top oenocyte-enriched proteins (CG7763, Lectin-22C, Lectin-galC1), which are all predicted secreted C-type Lectins, are only ranked #300, #183, and #479, respectively, in label-free data. Furthermore, many cytoplasmic proteins were identified in the hemolymph proteome but not tissue-labeling experiments, such as the muscle-expressed cytoplasmic proteins Myosin heavy chain (Mhc), Muscle-specific protein 300 kDa (Msp300), and Bent (Bt) (Supplementary Data 3b, d).

To evaluate potential bias against small proteins, we compared the proportion of ≤100 amino acid proteins across the *Drosophila* proteome, our label-free larval hemolymph proteome, and our high-confidence tissue secretome. Small proteins comprise ~6.0% of the fly proteome but were underrepresented in the hemolymph proteome (2.2%) and were modestly further reduced in the tissue–secretome dataset (1.9%) (Supplementary Fig. 10, Supplementary Data 3e). Thus, while detectable, small proteins are underrepresented, which is consistent with known limitations of proximity labeling, tryptic digestion, and LC–MS/MS^29,30^.

Among the list of 535 high-confidence secreted proteins, we identified 86 proteins with established roles in hemolymph (Fig. 1e, Supplementary Data 3b). This includes the same major hemolymph proteins observed in our non-quantitative tissue-secreted dataset (Supplementary Data 2a) such as apolipoproteins and extracellular matrix proteins 2a), as well as the hormone Myoglianin (Myo), Angiotensin converting enzyme (Ance), Imaginal disc growth factor 2/3 (Idgf2/3), and Secreted decoy of InR (SDR) (Fig. 1e, Supplementary Data 3a)^31–34^. In contrast, the remaining 449 proteins have no known role in the hemolymph, including 160 unnamed “CG” proteins. (Supplementary Data 3b).

We also identified peptides corresponding to GFP-TurboID from all 10 tissue-labeling samples (Supplementary Data 3f). This likely corresponds to auto-biotinylated GFP-TurboID that can bind streptavidin beads. Furthermore, GFP-TurboID enrichment was above the strict thresholds we used to identify the set of 535 tissue-secreted proteins for 8 out of the 10 tissues. Therefore, we explored the possibility that secreted GFP-TurboID leads to false-positive enrichment of hemolymph proteins. Theoretically, if this occurs, then the abundance of proteins in whole hemolymph samples would correlate with that from the streptavidin-enriched samples. Each TMT plex experiment contained raw blood as an internal control in addition to the enrichments from tissue-labeling samples (Fig. 1b), and we observed a low correlation between raw blood and enriched samples (Supplementary Fig. 11). In addition, little or no correlation was observed between the abundance of proteins in whole hemolymph samples (label-free dataset) and relative enrichment of proteins from the streptavidin-enriched samples (TMT dataset) (Supplementary Fig. 12, Supplementary Data 3g). Therefore, these data suggest that secreted GFP-TurboID does not label blood proteins, or the effect is negligible.

Next, we examined the tissue of origin of the 535 tissue-secreted proteins. The largest number of candidate proteins was observed from the fat and gut with 210 and 203 proteins, respectively, while oenocytes and neurons had the lowest number of candidate proteins with 19 and 52 proteins, respectively (Fig. 1f, Supplementary Data 3b). Unexpectedly, 319 (59.6%) high-confidence secreted proteins originated from an individual tissue (Fig. 1g, Supplementary Fig. 13, Supplementary Data 3b). Fat body shared the largest overlap of proteins across other tissues, with 110 identified from fat and at least one other tissue (Supplementary Data 3b). Interestingly, by comparing the tissue-enrichment of proteins to their abundance in bulk blood, we find that most proteins with greater tissue-enrichment had middle to low abundances in bulk blood (Supplementary Fig. 12). The exception was fat body, which is known to secrete extremely abundant proteins such as Apolipoprotein and Larval serum protein. This suggests that tissue-specific ER-labeling and streptavidin enrichment can increase the sensitivity of blood protein identification in addition to determining the tissue of origin. In support of this, we identified 24 proteins using tissue secretome labeling but not from bulk hemolymph (Fig. 1d, Supplementary Data 3b, d).

Unexpectedly, 64 of the 535 tissue-secreted proteins have a predicted transmembrane domain (Fig. 1c, Supplementary Data 3b). This includes Short gastrulation (Sog), which is known to be shed as an ectodomain^35^, as well as Calsyntenin (Cals), Neprilysin 4 (Nep4), and Dystroglycan (Dg), which have human homologs (Calsyntenin 1/2/3, Neprilysin, Dystroglycan, respectively) that are shed as ectodomains^36^, and CG6867 (see also below). Indeed, 100% of 184 unique peptides corresponding to 12 single-pass transmembrane proteins map only to the extracellular domain of the protein (Supplementary Data 3h). These results suggest that at least some of the transmembrane proteins in our list may be released into hemolymph as ectodomains.

### Bioinformatic functional analysis of tissue-derived secreted proteins

**Fig. 2:**
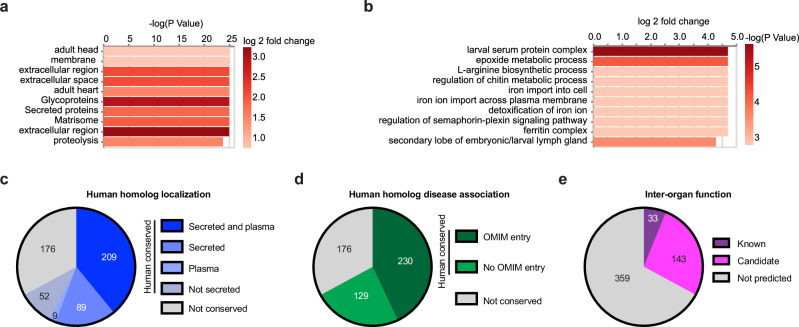
Functional bioinformatic analysis of 535 tissue-secreted proteins. Gene set enrichment with PANGEA on 535 tissue-secreted proteins. Top 10 enriched terms when ranked by **a** −log(*P* Value) and **b** log2-fold change. Bar colors indicate the opposite metric. PANGEA calculates *P*-values using a one-sided hypergeometric test. **c–e** Pie charts summarizing annotation of tissue-secreted proteins. **c** and **d** Human conservation based on whether at least one human homolog met the indicated criteria. **c** Subcellular localization of human homologs categorized as secreted or plasma proteins using the Human Protein Atlas. **d** Association of human homologs with genetic disease based on OMIM. **e** Known and predicted inter-organ proteins based on Flybase, DRSC, and FlyXCDB.

To help predict the functions of the 535 tissue-derived proteins, we employed a variety of bioinformatic tools. First, we employed gene set enrichment analysis (GSEA) using PANGEA^37^ (Fig. 2a, b, Supplementary Fig. 14a, Supplementary Data 4a). We found broad cross-tissue enrichment of terms related to the extracellular space, including “extracellular region”, “glycoproteins”, “basement membrane”, and “response to external stimulus”. Furthermore, we observed enrichment of hemolymph-related terms, such as “larval serum protein complex” and “ferritin complex”. Next, to identify possible tissue-specific biological functions, we analyzed each list of tissue-specific proteins using PANGEA (Supplementary Fig. 14b–d, Supplementary Data 4b), which showed several tissue-specific enrichments of pathway terms supported by previous publications, such as “NF-kappa B/Toll signaling pathway” for fat body^38^, “cuticle development” for trachea^39^, and “Extracellular Spatzle Activating Pathway Core Components” for hemocytes^40^. Functional enrichment analyses using an alternative method, g:Profiler^41^, showed similar results (Supplementary Fig. 15, Supplementary Data 4c), finding that all 10 tissues were enriched with terms indicative of secretion into the extracellular space, and other terms specific to individual tissues such as “extracellular matrix” (fat, glia, gut, hemocytes, muscle, neurons, trachea, wing disc), “defense response” (fat, glia, hemocytes), and “endopeptidase inhibitor activity” (fat, glia, gut, heart/nephrocytes, trachea). Together, these results suggest common extracellular functions and tissue-specific signatures.

We next examined human conservation of our 535 secreted proteins. 359 (67%) proteins have at least one human homolog (DIOPT score 3 or higher) (Fig. 2c and d, Supplementary Data 3b). Furthermore, 307 (57%) are homologs of human secreted and/or plasma proteins^2^ (Fig. 2c, Supplementary Data 3b). For example, CG6867, CG31999, and CG2493 are homologs of human proteins Olfactomedin-4 (OLFM4), Fibulin-1 (FBLN1), and Prolylcarboxypeptidase (PRCP), respectively, which have also been identified in plasma^42–44^. Furthermore, gut enterocyte-labeled CG9672 is a homolog of human Protein C, which is secreted from intestinal enterocytes and is defective in the inherited blood clotting disorder Protein C Deficiency^45^. Finally, 230 (43%) proteins have at least one human homolog with an OMIM entry (Fig. 2d, Supplementary Data 3b), and PANGEA predicts connections with human diseases enriched among the list of 535 proteins, including pre-eclampsia and myocardial infarction (Supplementary Fig. 16, Supplementary Data 4d). These results suggest that a large fraction of the tissue-derived secretome is conserved in humans and linked to disease.

To evaluate the usefulness of our dataset for studying inter-organ communication, we examined it for known and candidate factors. We identified at least 33 published inter-organ factors (Fig. 2e, Supplementary Data 3b), including the fat-secreted basement membrane protein Viking (Vkg), gut-secreted ferritin iron transporters, and muscle-secreted activin ligand Myo^31,46,47^. Interestingly, we observed unexpected tissues of origin for known inter-organ proteins, such as the basement membrane protein Nidogen (Ndg) from muscle, Torso-like (Tsl) hormone from fat body, and Ion transport peptide (ITP) hormone from muscle, which have been previously shown to be secreted from fat body, prothoracic gland, and neurosecretory cells, respectively^48–50^. Of the remaining proteins, at least 143 have properties suggestive of inter-organ function, including localization to the extracellular matrix, signaling pathway components, and domains involved in extracellular binding (Fig. 2e, Supplementary Data 3b)^37,51,52^. Examples include three muscle-secreted proteins with no prior inter-organ roles: (1) Short gastrulation (Sog), a BMP inhibitor^35^, (2) CG6867, an uncharacterized homolog of vertebrate Olfactomedin proteins^53^, and (3) Dally-like (Dlp), a heparan sulfate proteoglycan that binds and regulates morphogens like Wnt and Hedgehog^54^. Together, these results suggest our tissue secretome dataset is a rich resource for discovering new inter-organ proteins.

### A snRNA-seq atlas of *Drosophila* third instar larvae for mapping tissue-specific secreted proteins

To provide confidence in our dataset, we compared our list of tissue-secreted proteins to publicly available cell-type transcriptomic data of the encoding genes. First, we examined *Drosophila* transcriptomic data, including single-cell RNA-seq (scRNA-seq) or single-nucleus RNAseq (snRNA-seq) of the adult fly^55^, larval wing disc^56,57^, larval blood cells^58^, fly kidney^59^, and RNA-seq of dissected larval tissues^60^. These comparisons suggest that 529 (99%) of the genes encoding tissue-secreted proteins are expressed in the expected tissue type (Supplementary Data 5). While these data are encouraging, the majority of the transcriptomic datasets used for comparisons are not ideal, due to non-matching developmental timing (adult vs. larvae).

**Fig. 3:**
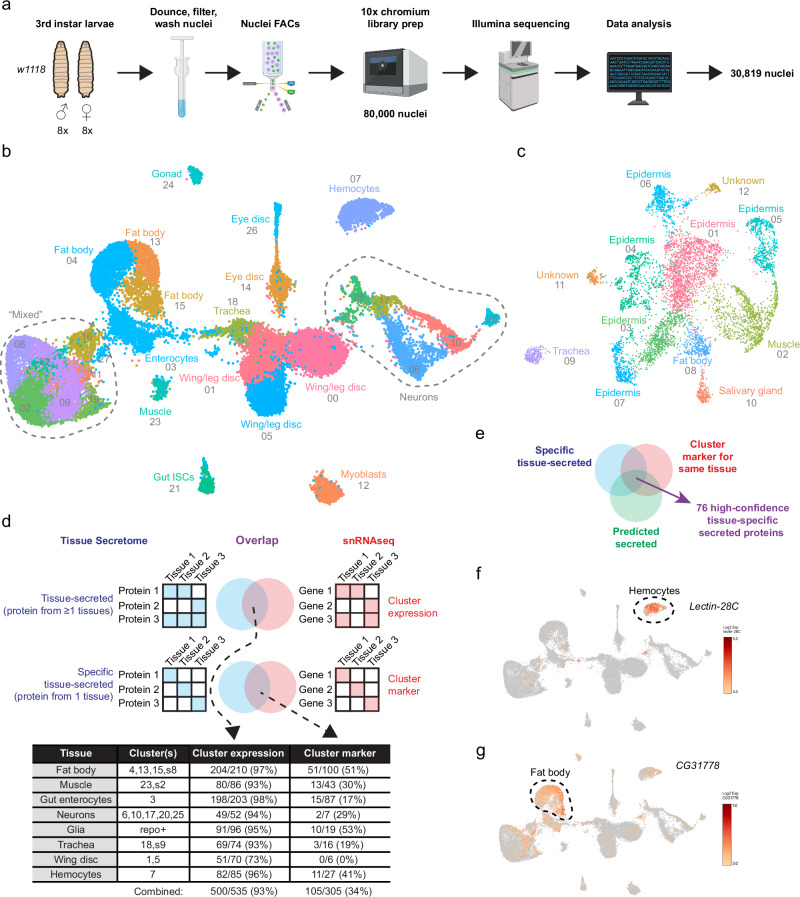
A 3rd instar larval single-nucleus RNA-seq map. **a** Workflow schematic of larval nuclear isolation, droplet sequencing, and analysis. Created in BioRender. Bosch, J. (2026) https://BioRender.com/ljt0gdw. **b** A single UMAP of the 3rd instar larvae containing 28 annotated cell-type clusters, including 8 “mixed” clusters that were re-clustered in (**c**). Resolution is 1.0. **c** A single re-clustered UMAP of the “mixed” cluster in **b**, annotated as 10 cell-types and 2 unknown cell-types. Resolution is 0.5. **d** Strategy to compare tissue secretome proteins with their encoding gene snRNA-seq expression (top). Table with overlap comparisons (bottom). **e** Venn diagram showing the approach to identify high-confidence tissue-specific secreted proteins. **f** and **g** Depiction of gene expression (heatmap) in UMAP in (**b**). Heatmap scale (right) and location of the highest cluster gene expression (dotted circle). Genes depicted are **f** *Lectin-28c* and **g** *CG31778*.

To expand on these comparisons, we generated our own single-nucleus RNA seq (snRNA-seq) atlas of wandering 3rd instar larvae to match the same developmental timepoint used for the tissue secretome screen (Fig. 3a). Through filtering steps, such as correcting for ambient RNA and removing doublets, we captured 30,819 nuclei and identified 28 cell-type clusters at resolution 1.0 (Fig. 3b, Supplementary Data 6a–b). Furthermore, we re-clustered eight poorly separated “mixed” clusters into 12 clusters at resolution 0.5 (Fig. 3c, Supplementary Data 6c). Using a panel of cell-type-specific marker genes from the literature and the Fly Cell Atlas as reference^61^, we annotated clusters into broad cell/tissue-types, including those used in our tissue–secretome screen (e.g., muscle, fat body) (Fig. 3b and c, Supplementary Data 6b–d, Supplementary Fig. 17a). For example, hemocyte marker genes are enriched in cluster 7, and gut enterocyte marker genes are enriched in cluster 3 (Supplementary Fig. 17b and c). In addition, using the glial marker gene *repo*, we identified 3476 putative glial nuclei and computed a list of marker genes (Supplementary Data 6e). Similar efforts to identify heart, nephrocyte, and oenocyte nuclei failed, perhaps because these cell types are less abundant. An interactive version of this 3rd instar larval snRNA-seq map can be found at https://www.flyrnai.org/scRNA/body_larvae/.

Next, we examined the overlap between our tissue-of-origin proteomic data and tissue-expression of the encoding genes. First, we counted how many genes encoding our 535 tissue-secreted proteins were expressed in the corresponding snRNA-seq cluster(s) (Fig. 3d, Supplementary Data 3b, Supplementary Data 6f–i). For example, of the 86 muscle-secreted proteins, 80 (93%) of the encoding genes were expressed in the muscle clusters. Second, to measure tissue-specificity, we counted how many genes encoding tissue-specific secreted proteins are marker genes for the same cluster (Fig. 3d, Supplementary Data 3b, Supplementary Data 6d, e, j). For example, of the 43 muscle-specific-secreted proteins, 13 (30%) of the encoding genes were muscle cluster marker genes. In total, these comparisons for 8 tissue types show that 93% (500 out of 535) of tissue-secreted proteins are expressed in at least one corresponding tissue cluster(s) and 34% (105 out of 305) of genes encoding tissue-specific-secreted proteins are marker genes for the expected cluster(s) (Fig. 3d).

Next, to identify possible ligand–receptor relationships between cell-types and organs, we analyzed our snRNA-seq data using FlyPhoneDB2^62^ (Supplementary Fig. 18, Supplementary Data 6k). The results highlight published local cell–cell interactions and distant inter-organ interactions, including Slit-Roundabout2 signaling in neurons^63^, Serrate-Notch signaling from imaginal discs to trachea by direct contact^64^, and Myoglianin–Thickveins from muscle to imaginal discs via circulation^31^. In addition, we identified putative inter-organ interactions involving established signaling ligands, such as Slit-Syndecan signaling from neurons to gut intestinal stem cells (ISCs) and Wnt4-Frizzled/Frizzled2 signaling between myoblasts and neurons. Integrating these results with our tissue–secretome dataset further revealed candidate inter-organ ligands, including muscle-secreted CG10359 binding to Multiple edematous wings (Mew) in imaginal discs, fat-body-secreted CG16704 binding to Stubble (Sb) in imaginal discs, and hemocyte-secreted Lectin-28c binding to Toll (Tl) in hemocytes. Together, these analyses demonstrate that combining snRNA-seq with tissue–secretome profiling can uncover both known and candidate ligand–receptor interactions within and between organs.

Finally, we defined a list of 76 high-confidence tissue-specific secreted proteins as (1) tissue-specific proteins from our proteomic screen, (2) encoded by marker genes for the same tissue cluster(s), and (3) predicted to be secreted or transmembrane (Fig. 3e, Supplementary Data 6l). In this list, we found known tissue-specific hemolymph proteins, such as Myo from muscle, Nimrod B1 (NimB1) from hemocytes, and Adenosine deaminase-related growth factor D (ADGF-D) from fat body (Supplementary Data 6l). Interestingly, this list contains mostly uncharacterized proteins, such as Lectin-28C and CG31778, which are enriched in hemocytes and fat body, respectively (Fig. 3f and g).

### A collection of knock-in flies to characterize tissue-specific secreted proteins in vivo

**Fig. 4:**
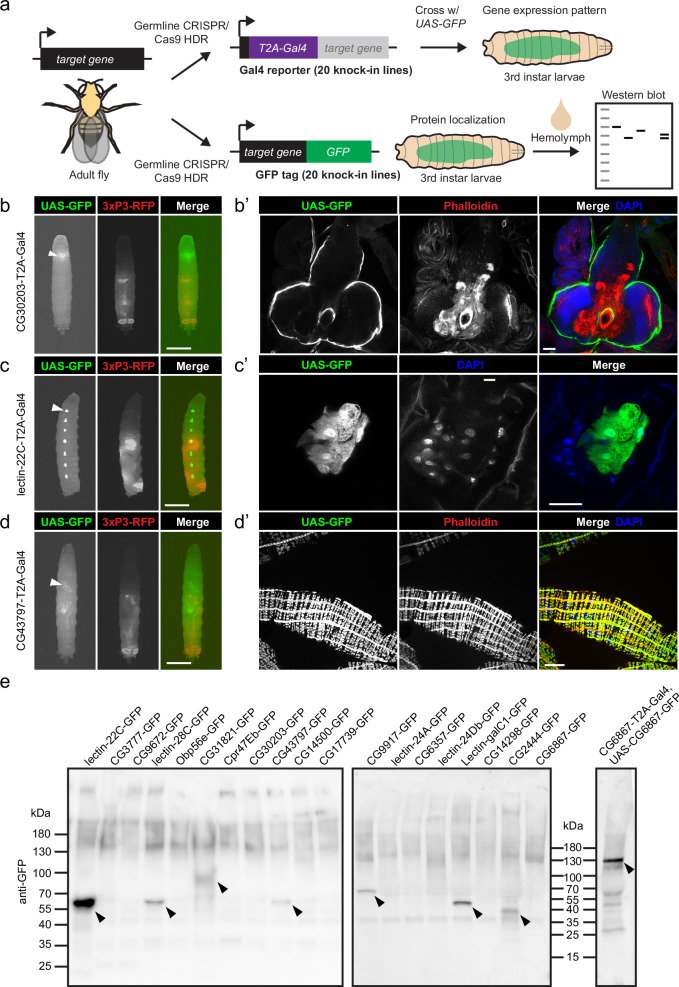
In vivo characterization of hemolymph proteins using knock-in fly lines. **a** Schematic of generating fly knock-ins and experimental use to assay gene expression and protein localization. **b–d** Widefield fluorescence microscopy images of whole 3rd instar larvae expressing *UAS-GFP* (green) under the control of the *gene-T2A-Gal4* transgene. Also shown is *3xP3-RFP* (red) marker fluorescence. Arrowheads indicate localized GFP fluorescence in the **b** brain, **c** oenocytes, and **d** visceral gut muscle. Scale bar is 1 mm. Experiments were repeated twice independently with similar results. **b’–d’** Confocal microscopy images of dissected tissues from 3rd instar larvae, showing anti-GFP (green), phalloidin-660 (red), and DAPI (blue) stains. Scale bars are 50 µm. **b–b’** *CG30203-T2A-Gal4*, **c–c’** *lectin-22C-T2A-Gal4*, **d–d’** *CG43797-T2A-Gal4*. **b’** and **c’** are confocal slices, **d’** is a projection. **e** Anti-GFP western blots of 3rd instar larval hemolymph collected from GFP-tag lines. Arrowheads indicate bands at the expected molecular weight. (58.8 kDa = Lectin-22c-GFP; 59.2 kDa = Lectin-28c-GFP; 77.4 kDa = CG31821-GFP; 56.2 kDa = CG43797-GFP; 63.1 kDa = CG9917-GFP; 50.8 kDa = Lectin-galC1-GFP; 41.4 kDa = CG2444-GFP; 134.3 kDa = CG6867-GFP). Experiments were repeated twice independently with similar results.

Next, we used in vivo knock-in fly strains to test if proteins from our proteomic screen are expressed in the expected tissue, present in circulation, and/or localize non-autonomously to distant tissues (Fig. 4a). We chose to target 22 proteins based on their tissue-specific expression, lack of characterization, presence in the blood, and prediction as secreted/transmembrane (Supplementary Data 7a and b). Included in this list are seven C-type lectin proteins (e.g. lectin-22C), six proteases/protease-inhibitors (e.g. CG31821), two proteins with no predicted functional domains or characterization (CG3777, CG9917), and the transmembrane protein CG6867 (see also above).

We generated knock-in fly strains using CRISPR/Cas9 and homology-directed repair (HDR) in the fly germline as previously described^65^. To analyze gene expression, we inserted a *T2A-Gal4* reporter gene^66^ into the 5’ coding sequence (Fig. 4a). These *T2A-Gal4* alleles are predicted to be null, as the insertion truncates the endogenous protein. To analyze protein localization, we inserted *GFP* into the 3’ coding sequence to create C-terminal GFP fusion proteins (Fig. 4a). To facilitate the creation of these knock-in lines, donor plasmids containing the positive selection marker *3xP3-RFP*^65,67^ were modified to include the negative selection marker *GMR-eya(shRNA)*, which helps identify unwanted off-target plasmid integration^68^ (Supplementary Fig. 19a and b). In total, we generated 20 *T2A-Gal4* and 20 GFP-tag knock-in fly strains (Fig. 4, Supplementary Data 7). Consistent with previous reports^68^, donor backbone integration into the germ line was common. For example, of the 88 injected G0 males that produced *3xP3-RFP*-positive progeny, 42 (48%) also produced progeny carrying the *GMR-eya(shRNA)* marker (Supplementary Fig. 19c, Supplementary Data 7).

Gene expression patterns were visualized by crossing *T2A-Gal4* lines to *UAS-GFP* flies and imaging GFP fluorescence in 3rd instar larvae (Fig. 4a). 19 out of 20 lines exhibited expression in the expected tissue(s) (Supplementary Data 7a, Fig. 4b–d, Supplementary Fig. 20). This includes cases where Gal4 expression closely mirrors that seen in our tissue–secretome data, such as *CG30203-T2A-Gal4*, *Lectin-22c-T2A-*Gal4, and *CG43797-T2A-Gal4*, which are specifically expressed in perineural glia, oenocytes, and gut visceral muscle, respectively (Fig. 4b–d, Supplementary Data 7a). One line did not match our TurboID-labeling experiments—*Obp56e-T2A-Gal4* is expressed primarily in tracheal spiracles (Supplementary Fig. 20), however Obp56e was enriched from heart/nephrocyte and wing disc (Supplementary Data 3b). Since *Obp56e* transcript is not detected in wing discs^57^ (Supplementary Data 6f) or larval nephrocytes^69^, we believe that this is a false positive. One possibility is that the wing disc Gal4 driver (*MS1096-Gal4*) is expressed in tracheal spiracles.

Next, we determined if GFP-tagged target proteins were circulating in hemolymph. We collected hemolymph from 3rd instar larvae and performed anti-GFP western blotting. Encouragingly, we detected a band at the expected molecular weight for 7 out of 19 GFP-tagged lines (Fig. 4e, Supplementary Data 7b). Only 2 out of 5 positive control GFP-tagged proteins were detected using this method (Supplementary Fig. 21), indicating a high false-negative rate and likely explaining why the remaining 11 GFP-tagged proteins were not detected in hemolymph. Interestingly, gene expression and protein band detection are not correlated by a Mann–Whitney test (Supplementary Data 7c), indicating that lack of GFP-tagged protein detection cannot be explained simply by lower gene expression.

**Fig. 5:**
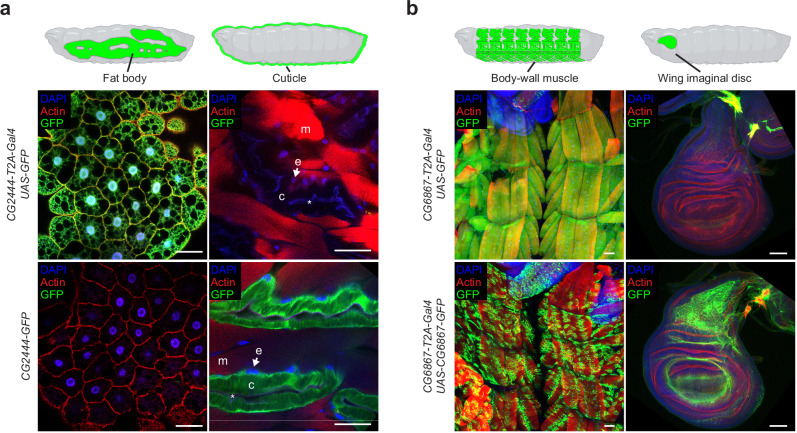
Inter-organ localization of hemolymph proteins. **a** and **b** Confocal microscopy images of dissected tissues from 3rd instar larvae with indicated genotypes, showing anti-GFP (green), phalloidin (red), and DAPI (blue) stains. Autofluorescence of the cuticle is also shown in blue. Images of the same tissue were acquired using identical confocal settings to allow direct comparison of fluorescence intensity. Scale bars are 50 µm. Schematic above confocal images show the location of indicated tissues/structures in 3rd instar larvae. Created in BioRender. Bosch, J. (2026) https://BioRender.com/6etdpnn Experiments were repeated independently twice with similar results. **a** Images are confocal slices. m = muscle, e = epidermal cell nuclei, c = cuticle, asterix (*) = outside face of cuticle. **b** Images are confocal projections.

Finally, to identify evidence of inter-organ trafficking, we imaged GFP fluorescence in 3rd instar larvae knock-in animals and systematically compared each gene’s tissue-specific expression pattern (Gal4 gene reporter) with its protein localization (GFP protein fusion) (Fig. 4a) across 19 genes. Specifically, we searched cases in which GFP-tagged proteins localized to tissues distinct from their site of synthesis. For CG2444, gene expression was restricted to the fat body, yet the protein localized to the cuticle (Fig. 5a), an important extracellular matrix on the surface of the animal. Similarly, CG6867 was expressed in somatic muscle, and the protein accumulated in wing imaginal discs (Fig. 5b), which develop into the adult wing. Both CG2444-GFP and CG6867-GFP were detected in hemolymph (Fig. 4e), supporting their release into circulation by secretion and shedding, respectively. Notably, for CG6867, we only detected the protein on wing imaginal discs and in the blood using a *UAS-CG6867-GFP* transgene and not with the endogenous CG6867-GFP knock-in line, suggesting the endogenous signal may be below the detection limit. Together, these results suggest that CG2444 is trafficked from fat-to-cuticle, and CG6867 is trafficked from muscle-to-wing imaginal disc.

In summary, our knock-in fly strains validate our tissue–secretome dataset as biologically accurate and containing tissue-specific hemolymph proteins and candidate mediators of inter-organ communication.

## Discussion

**Fig. 6:**
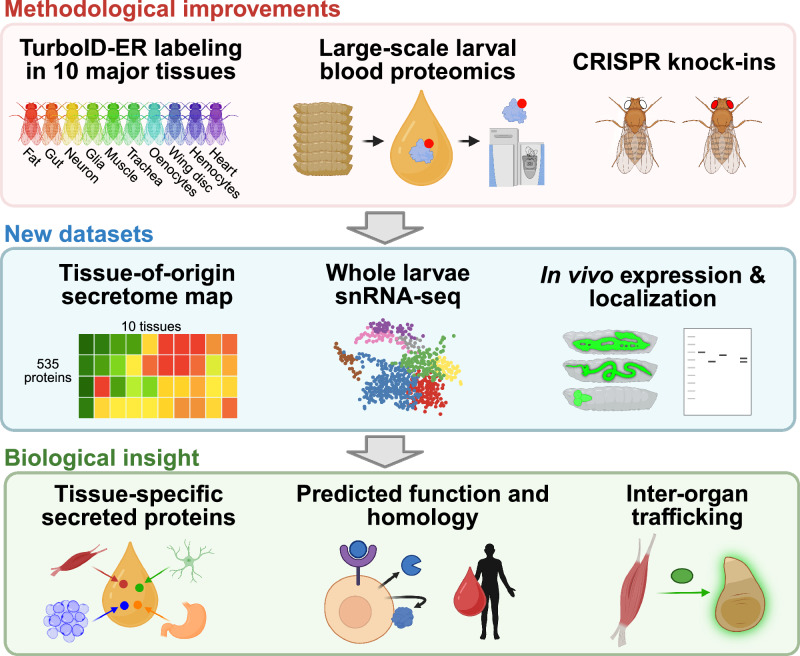
Schematic summary of *Drosophila* tissue–secretome mapping and major findings. Created in BioRender. Bosch, J. (2026) https://BioRender.com/zfrfle5.

This study presents a comprehensive in vivo tissue–secretome map in *Drosophila melanogaster* by integrating proximity labeling, mass spectrometry, single-nucleus RNA-seq, and CRISPR knock-in technology (Fig. 6). We report genetic tools, protocols, and datasets that will broadly benefit the scientific community working on secreted proteins, hemolymph proteins, and inter-organ communication. For example, our datasets can help assign functions to uncharacterized proteins and may be a source of human–fly conserved tissue biomarkers.

We report cell lines and fly strains for labeling secretory proteins in *Drosophila*. In particular, our *UAS-GFP-TurboID-ER* fly lines are compatible with thousands of Gal4 lines^70^ to label secreted proteins in a cell-type-specific manner. Furthermore, generating two-transgene *X-Gal4, UAS-GFP-TurboID-ER* fly lines is made simple by screening for GFP fluorescence in animals. We provide 10 such *GFP-TurboID-ER* lines in this study, which express in major tissue types (e.g., muscle, glia, neurons, etc.) and should be useful to the *Drosophila* community to identify secreted proteins or intracellular ER/Golgi-resident proteins.

In addition, we report optimized protocols for isolating *Drosophila* hemolymph and streptavidin pulldowns. Previous hemolymph collection methods from larvae or adults were not scalable for large-scale streptavidin pulldowns and often do not remove hemocytes and cell debris^20,71,72^. Our protocol yields >3 mg of soluble hemolymph proteins in ~30 min, which is comparable to human blood plasma samples^73^ and is sufficient for streptavidin pulldown and LC–MS/MS^74^. We used larvae because they yield large amounts of hemolymph^75–77^, but this protocol could be adapted for adult flies. Similarly, while we focused on soluble proteins, this protocol could be applied to circulating exosomes, metabolites, and extracellular RNA. Finally, we empirically determined a wash buffer (2% SDS and 4 M Urea) that reduces non-specific binding of abundant LSP proteins to streptavidin beads. While it is unclear whether LSP proteins are representative of other non-specific binders, the inclusion of SDS and high urea concentrations is likely beneficial. Similar conditions have been used by others to reduce background binding to streptavidin beads^78–81^.

We report proteomic datasets of secreted proteins in *Drosophila*. We identified 72 proteins secreted from S2R+ cells, including uncharacterized proteins such as CG5080. Importantly, we report an in vivo tissue–secretome map of 535 proteins in *Drosophila* hemolymph from 10 major tissue types, most of which have no previously described role in hemolymph. The predominance of fat body-derived proteins is expected, as this tissue is functionally analogous to the mammalian liver, the primary source of circulating plasma proteins^82,83^. Unexpectedly, more than half of the circulating proteins originated from a single tissue. While other studies have applied in vivo TurboID-ER labeling in mice^8,11–16,18^, often focusing on a limited number of tissues, our study extends this approach to 10 major tissue types to facilitate cross-tissue analysis of the secretome. In contrast to a previous study that identified protein trafficking between tissues in *Drosophila*^18^, our study focused on circulating proteins, offering a more scalable approach using hemolymph-only collection and enabling direct cross-sample comparison. Finally, using label-free MS of larval hemolymph, we identified 4566 proteins, which is four times more than a previous study^20^. Importantly, tissue-specific labeling identified circulating proteins not observed in bulk hemolymph. Together, these proteomic resources represent a comprehensive dataset of larval *Drosophila* secreted and hemolymph proteins.

Bioinformatic analysis of the 535 tissue-secreted proteins indicates that this dataset is a valuable resource for interrogating extracellular and inter-organ biology. These proteins are enriched for extracellular functions and for tissue-specific pathways consistent with known biology. Notably, we defined at least 143 proteins with annotated features suggestive of inter-organ function, and three proteins that may signal through candidate receptors, providing a foundation for future studies. The large degree of conservation between these fly proteins and human secreted, circulating, and disease-associated proteins underscores the relevance of these data beyond *Drosophila*.

We report a snRNA-seq atlas of whole third instar *Drosophila* larvae, which we used to compare our proteomics results. While previous studies profiled dissected larval tissues^56–58^, we generated a snRNA-seq dataset from intact whole larvae, and it is publicly accessible at flyrnai.org. Interestingly, our snRNA-seq dataset revealed at least two clusters of unknown identity. We identified cell-types corresponding to 8/10 tissues used for TurboID labeling and found convincing overlap between the genes expressed, as well as tissue-specific expression. We did not identify oenocytes and heart/nephrocytes, perhaps because our dataset was relatively small (~30k nuclei) and these cell-types are less frequent. In addition, not all tissue-specific proteins corresponded to cluster-specific marker genes in the snRNA-seq data, which may reflect post-transcriptional regulation, regulated secretion, or limitations in the sensitivity of either method.

We report a collection of 40 CRISPR/Cas9 knock-in fly strains to investigate the quality of our proteomics data. All but one Gal4 line expressed in the expected tissue(s), including specific patterns in oenocytes, fat body, and hemocytes, as well as sub-cell-types within tissues, such as perineural glia and enterocytes in a sub-region of the midgut. These Gal4 lines may be valuable tools for gene-function studies, including tissue-specific drivers as well as loss-of-function alleles. For example, *CG31821-Gal4* and *CG14298-Gal4* are expressed in circular gut muscles and mouth hook muscles, respectively, which, to our knowledge, are not represented by existing Gal4 drivers. Many of our GFP knock-in lines showed GFP signal in hemolymph or localized to distant tissues, confirming their secretion. Because we generated knock-in fly strains for largely uncharacterized genes, this in vivo data opens areas of investigation. For example, *CG31821-T2A-Gal4* is homozygous lethal, suggesting this gene is essential, and may have functions related to visceral muscle like its human homolog Serine carboxypeptidase 1 (SCPEP1), which is expressed in smooth muscle^84^ and present in plasma^1^. Finally, we showed tissue-specific expression of seven uncharacterized C-type lectins (e.g., Lectin-22c) in the gut, muscle, oenocytes, fat body, and hemocytes. Given the established roles of C-type lectins in innate immunity, their distinct expression patterns may indicate coordinated immune functions across multiple tissues.

Importantly, the generation of these knock-in lines was facilitated by our improved donor plasmids incorporating both positive and negative visible selection markers. Donor backbone integration is a frequent outcome of CRISPR-HDR genome editing^67,68^, yet positive selection markers alone do not distinguish these imprecise events. Our inclusion of a separate marker on the backbone, as used by others^67,68^, enables simple exclusion of backbone integration events prior to labor-intensive molecular validation, substantially reducing screening effort. Thus, these improved *T2A-Gal4* and GFP-fusion donor plasmids should facilitate knock-in generation at additional loci.

Using our knock-in fly strains, we identified two uncharacterized proteins that localize to tissues distinct from their site of synthesis, suggesting they have inter-organ functions. CG2444 belongs to the single von Willebrand factor C-domain (SVC proteins) family^85^, which is thought to respond to environmental challenges. Since the fat body is known to be an integrator of metabolic and immune changes^86^, and the cuticle is a barrier to the environment, including pathogens^87^, CG2444 may be a remote factor from the fat body that alters cuticle composition or function. Our results also suggest a mechanism of CG2444-GFP transcytosis through epidermal cells, like that observed for Serp transcytosis through tracheal cells^88^. CG6867, a transmembrane protein, is the single fly ortholog of vertebrate olfactomedins and closely resembles the human transmembrane protein Gliomedin (GLDN)^53^. Since the ectodomain of Gliomedin is known to be shed^89^ and binds to the extracellular matrix (ECM) protein Perlecan^90^, we hypothesize that the ectodomain of CG6867 is shed from muscle and integrates into the ECM of distal tissues such as the wing imaginal discs. This finding, together with our identification of candidate ectodomains of other transmembrane proteins, suggests that shedding may be an understudied source of circulating inter-organ factors. Future studies of these two candidate inter-organ factors should investigate their roles in target tissues.

An important concern is that TurboID-ER may be secreted and label proteins extracellularly, thus confounding the tissue-specificity imparted by its genetic expression. Indeed, we detected GFP-TurboID-KDEL in S2R+ media and in larval hemolymph. This could be explained by overwhelming the KDEL receptor or by cell culture artifacts, such as cell lysis, and may be addressed by reducing TurboID-ER expression. However, several lines of evidence suggest that extracellular labeling is minimal and does not confound our results. First, ATP levels are low in the extracellular space^91^ and extracellular labeling with TurboID requires added ATP^92^. Second, we saw minimal labeling of abundant cow proteins (from fetal bovine serum) in S2R+ cell culture media, which are not visible on protein gels but are detectable by MS. Third, we saw tissue-specific labeling in vivo rather than widespread labeling of abundant proteins, and the proteomic profiles from tissue-specific labeling vs. total hemolymph were distinct. Finally, the use of a KDEL sequence to retain promiscuous biotin ligases in the ER has been used successfully by others to label tissue-specific secreted proteins^8,11,13–16,18^. Together, these results indicate that while some TurboID-ER is secreted, it does not impact the specificity of our secretome-mapping strategy.

Another concern is artifacts caused by TurboID labeling. For example, our list of 535 tissue-secreted proteins contains a small number of ER-resident, cytoplasmic, and transmembrane proteins. TurboID-ER expression could, in theory, cause biotinylated proteins to mislocalize, such as by protein misfolding, ER-stress, or saturation of KDEL receptors. However, 96% of the proteins identified in our tissue–secretome map were also present in hemolymph from wild-type animals, and our fly strains constitutively expressing TurboID-ER were viable and fertile, arguing against widespread mislocalization or ER-stress toxicity. Furthermore, GFP-tagged knock-in lines confirmed hemolymph secretion for many of these proteins. We also speculate that non-secretory proteins were identified due to mislocalization of TurboID-ER into the cytoplasm and subsequent cell leakage, either of which could be tissue-specific. Indeed, we identified seven ribosomal proteins (e.g., RpL8) specifically from muscle. Finally, transmembrane proteins likely represent shed ectodomains rather than intact membrane proteins, as supported by peptide mapping and their lack of exosome annotations. Therefore, we argue that these possible artifacts do not impede us from identifying true secreted proteins.

We note important limitations with our in vivo TurboID-labeling strategy to identify secreted proteins in hemolymph. Since TurboID-ER labels surface-exposed lysine residues on proteins in the secretory pathway, LC–MS/MS may simply miss proteins that are low abundance, small, or otherwise have few accessible lysines. For example, we did not identify Dilp or Unpaired hormones, although other hormones such as Myo and ITP were identified. In addition, our approach should not identify secreted proteins that bypass the ER or remain locally deposited. Conversely, some proteins identified in hemolymph may not function there, but instead were released by tissue leakage, blood collection, or biotin labeling. These could include ER resident proteins, ECM-deposited proteins, or proteins from other extracellular locations (e.g., tracheal lumen, gut lumen, cuticle). For example, we identified the iotaTrypsin digestive enzyme from gut enterocytes and the ER-resident protein ERp44 from six tissues. Finally, because our analysis was limited to 3rd instar larvae, some proteins relevant to adult physiology or translational contexts may not have been identified.

Additional limitations arise from the use of tissue-specific Gal4 drivers, as their strength, timing, and specificity can influence whether proteins are detected and correctly assigned to their tissue(s) of origin. Weak drivers may produce insufficient TurboID-ER labeling, whereas strong drivers (e.g. *lpp-Gal4*) could increase protein recovery. Temporal expression differences may also limit the detection of proteins secreted earlier in development. Furthermore, some drivers exhibit off-target expression (e.g., *repo-Gal4* and *MS1096-Gal4* in salivary glands, *lpp-Gal4* in glia^93^), which could lead to incorrect tissue assignments. Conversely, some drivers may be too spatially restricted to capture the full secretome of a tissue (e.g., *MS1096-Gal4*, which is limited to the wing disc pouch). Finally, some secreted proteins undergo endocytosis and subsequent re-secretion^94^, which could confound tissue-of-origin assignments if labeling occurs in recipient tissues. For example, we identified Apolpp from muscle in addition to its established fat body origin, though *apolpp* is expressed in muscle^55^ (https://www.flyrnai.org/scRNA/body_larvae/).

In addition to experimental limitations, there is an inherent trade-off between sensitivity and specificity in defining high-confidence tissue-secreted proteins. We set tissue-specific fold-change thresholds to minimize false discoveries and present a highly confident protein set, consistent with common proteomics practice to reduce costly follow-up on false leads. The empirical FDR for these thresholds ranged from 0% to 13.8%, within commonly used ranges (typically ~5%). However, minimizing false discoveries increases false negatives, and proteins not identified as secreted should not be interpreted as truly absent from circulation. Accordingly, our thresholds represent a conservative guideline, and more relaxed thresholds could expand candidate lists depending on tolerance for false discoveries and availability of supporting biological evidence.

An additional limitation concerns the use of C-terminal GFP knock-in alleles to validate circulating proteins by western blot. Although GFP tagging enables direct detection, it may alter folding, stability, trafficking, or proteolytic processing, especially for secreted proteins whose maturation or cleavage may separate GFP from the circulating fragment. Low expression or protein accumulation, combined with a high anti-GFP background, may also cause false negatives. Consistent with this, we observed similarly high false-negative rates for experimental knock-in lines and known hemolymph proteins (Fig. 4e, Supplementary Fig. 21), indicating that limited detection sensitivity is an inherent assay limitation rather than evidence of failed secretion.

Our datasets provide a foundation for further exploration and utility. Researchers can search our datasets to investigate specific proteins/tissues of interest, and we have already experimentally identified at least two candidate inter-organ proteins, highlighting their potential to discover new biology. Additionally, this resource may facilitate the discovery of human biomarkers, as illustrated by our identification of over 300 fly proteins whose human homologs are secreted or detected in plasma. Future studies employing additional Gal4 drivers could expand coverage to tissues not examined here, such as the ring gland or epidermis. Furthermore, this method could be applied to disease models to identify tissue-specific induced factors that promote or prevent pathology. Finally, our tissue-specific TurboID-ER labeling and large-scale collection approach could be adapted to other species, including insect disease vectors, and to other extracellular compartments beyond hemolymph, such as the gut lumen, cuticle, or proteins transferred between individuals.

## Methods

### Plasmid cloning

Plasmid DNAs were constructed and propagated using standard protocols. Briefly, chemically competent TOP10 *E. coli* (Invitrogen, C404010) were transformed with plasmids containing either Ampicillin or Kanamycin resistance genes and were selected on LB-Agar plates with 100 µg/mL Ampicillin or 50 µg/mL Kanamycin. Similarly, gateway destination plasmids containing the *ccdB* toxic gene were propagated in *ccdB*-resistant *E. coli* (Invitrogen, A10460) by culturing in LB containing 100 µg/mL Ampicillin and 25 µg/mL Chloramphenicol. Oligo and dsDNA sequences are in Supplementary Data 8a.

*pMT-EGFP-V5* (Addgene #240240, DGRC #1696) was generated by EcoRI/XbaI (NEB, R3101S, R0145S) restriction enzyme digestion and ligation of PCR-amplified *EGFP* sequence into *pMT/V5-His B* (Invitrogen, V4120-20).

#### Entry vectors

Construction of pEntr vectors (for Gateway cloning) was performed by Gibson assembly (NEB, E2621S) of PCR amplified backbone from *pEntr-dTOPO* (Invitrogen C4040-10) and PCR-amplified gene coding sequence (when appropriate, with or without stop codon).

*pEntr_sfGFP-TurboID* (Addgene #240226, DGRC #1682) and *pEntr_BiP-sfGFP-TurboID-KDEL* (Addgene #240223, DGRC #1679) were generated by Gibson assembly of overlapping PCR-amplified fragments containing *sfGFP*, *TurboID*, and *pEntr* backbone sequences. *sfGFP* (superfolder GFP) was amplified from *pCRISPaint-sfGFP-3xP3-RFP* (Addgene #127566)^65^. *TurboID* was amplified from *pUAS-V5-TurboID-NES* (Addgene #116904)^6^. *BiP* and *KDEL* sequences were added by oligos.

*pEntr_HA-spGFP11* (Addgene #240224, DGRC #1680) was generated by Gibson assembly of a *HA-spGFP11* gBlock DNA fragment into *pEntr* backbone sequence.

*pEntr_spatzle-HA* (Addgene #240227, DGRC #1683) and *pEntr_hhN-HA* (Addgene #240225, DGRC #1681) were generated by Gibson assembly of overlapping PCR-amplified fragments containing *spatzle* or *hhN*, and *pEntr_HA-spGFP11* backbone sequences. *spatzle* sequence was amplified from larval cDNA. *hhN* was amplified from *Actin Hh-FL*^95^. hhN is an N-terminal portion of Hh that normally results after protein cleavage. Full-length spatzle can be cleaved by Easter^96^; however, this is unlikely to occur in S2R+ cells, which express low levels of Easter (DGET, https://www.flyrnai.org/tools/dget/web/). In both cases, the C-terminal HA tag is fused to the mature signaling portion of the protein.

*pEntr_CG6867_nostop* (Addgene #240238, DGRC #1694) was generated by Gibson assembly of PCR-amplified coding sequence into *pEntr* backbone sequence. *CG6867* was amplified from cDNA clone FI21454 (DGRC #1661063).

#### Gateway vectors

*pWal10-roe-sfGFP* (Addgene #240239, DGRC #1695) was generated by PCR amplification of the *sfGFP* sequence from *pCRISPaint-sfGFP-3xP3-RFP* (Addgene #127566)^65^ and ligation into *pWal10-roe*^97^ that was digested with XbaI (NEB, R0145).

#### Gateway cloning LR reactions

*pMT-sfGFP-TurboID* (Addgene #240231, DGRC #1687)*, pMT-sfGFP-TurboID-ER* (Addgene #240230, DGRC #1686)*, pUAS-sfGFP-TurboID-ER* (Addgene #240232, DGRC #1688), pMT-Myc-BirA*G3 (Addgene #240233, DGRC #1689), pMT-Myc-BirA*G3-ER (Addgene #240234, DGRC #1690)*, pUAS-spatzle-HA* (Addgene #240235, DGRC #1691)*, pUAS-hhN-HA* (Addgene #240236, DGRC #1692), and *pUAS-CG6867-sfGFP* (Addgene #240237, DGRC #1693) were constructed by Gateway cloning. Cloning reactions were performed using LR Clonase II Enzyme mix (Invitrogen 11791-020). Published entry plasmids used were pEntr_Myc-BirA*-G3 and pEntr_Myc-BirA*G3-ER (Addgene # 173814)^18^. Published destination plasmids used were *pWalium10-roe*^97^, *pValium10-roe*^97^, and *pMK33-GW*^98^. See Supplementary Data 8b for information on assembled gateway plasmids.

#### Scarless GFP and Gal4 HDR donor plasmids with a GMReya(shRNA) marker

*pHD-sfGFP-ScarlessDsRed-GMR-eya(shRNA)* (Addgene #240228, DGRC #1684) and *pHD-T2A-Gal4-3xP3DsRed-GMR-eya(shRNA)* (Addgene #240229, DGRC #1685) were cloned by first mutating the EcoRI site in *pBS-GMR-eya(shRNA)* (Addgene #157991)^68^ using site-directed mutagenesis (NEB, E0554S) to produce *pBS-GMR-eya(shRNA)_EcoRImut* (Addgene #240222, DGRC #1678). *sfGFP-ScarlessDsRed* was PCR amplified from *pHD-sfGFP-ScarlessDsRed* (Addgene # 80811) (flycrispr.org)^67^, and *T2A-Gal4-ScarlessDsRed* was PCR amplified from Bosch CRISPaint plasmid *pCRISPaint-T2A-GAL4-Hsp70*^99^. Each fragment was cloned into *pBS-GMR-eya(shRNA)_EcoRImut*, which had been digested with SacI and HindIII (NEB, R3156S, R3104S), by Gibson assembly.

#### Cloning homology arms into donor plasmids

For T2A-Gal4 knock-ins, 1–2 kb genomic homology arms were designed that flank a sgRNA target cut site in the 5’ coding sequence. Homology arms were PCR amplified from *nos-Cas9* fly genomic DNA, such that genes on Chr. X or II were amplified from *nos-Cas9attP2*, and genes on Chr. III were amplified from *nos-Cas9attP40*.

For sfGFP knock-ins, 1–2 kb genomic homology arms were designed that flank a sgRNA target cut site near the start (for N-terminal fusion) or stop codon (for C-terminal fusion). Homology arms were amplified as for T2A-Gal4 knock-ins. In addition, we introduced 4–5 mutations in the PAM and spacer sequence that introduce synonymous codons in the coding sequence. Mutations were encoded on PCR primer 5’ extensions or a synthesized dsDNA fragment that bridged the wild-type homology arm and the insert.

PCR amplified homology arms, and in some cases synthesized dsDNA, were assembled in a Gibson reaction with *pHD-T2A-Gal4-ScarlessDsRed-GMR-eya(shRNA)* that had been digested with EcoRI-HF (NEB, R3101S), or *pHD-T2A-Gal4-ScarlessDsRed-GMR-eya(shRNA)* that had been digested with AscI/SacI-HF (NEB, R0558S, R3156S). In some cases, correctly assembled donor plasmids were screened by colony PCR using common primer pairs that flank the homology arms.

#### sgRNA plasmid cloning

sgRNA target sites were selected using the Find CRISPRs tool (v3.1.0) (https://www.flyrnai.org/crispr3/web/)^100^. Plasmids encoding sgRNAs were constructed by annealing oligos and ligating into *pCFD3* (Addgene # 49410)^101^ digested with BpiI (Thermo Fisher, ER1011), or by PCR amplifying dual sgRNA sequences and Gibson cloning into *pCFD5* (Addgene # 73914)^102^ digested with BpiI.

### S2R+ cell culture

*Drosophila* S2R+ cells^27^ were cultured at 25 °C using Schneider’s media (21720-024; Thermo Fisher Scientific) with 10% fetal bovine serum (A3912; Sigma) and 50 U/mL penicillin–streptomycin (15070-063; Thermo Fisher Scientific). Cells were transfected using Effectene (301427; Qiagen) following the manufacturer’s instructions.

For testing TurboID constructs by imaging, S2R+ cells were transfected with *pMT-sfGFP-TurboID* or *pMT-sfGFP-TurboID-ER* plasmids in six-well plates. Four days after transfection, 100 μM CuSO_4_ and 50 μM biotin (Sigma, B4639-1G) were added to induce gene expression and promote biotinylation, respectively. 24 h later, cells were resuspended, diluted to 1 × 10^6^ cells/mL, and transferred to 384-well glass plates (PerkinElmer, 6007558).

For testing labeling of Spz and hhN secreted proteins, S2R+ cells were transfected in six-well plates with *pAct-Gal4* (Y. Hiromi, National Institute of Genetics, Mishima, Japan), and *pUAS-spatzle-HA* or *pUAS-hhN-HA*, and *pMT-GFP-V5* or *pMT-sfGFP-TurboID-ER*.

*S2R*+*-MT-sfGFP-TurboID* (DGRC #350) and *S2R*+*-MT-sfGFP-TurboID-ER* (DGRC #351) stable cell lines were generated by transfecting S2R+ with *pMT-sfGFP-TurboID* or *pMT-sfGFP-TurboID-ER* plasmids in six-well plates. These *pMK33*^42^-derived plasmids contain a Hygromycin resistance gene and a *Metallothionein* promoter to induce gene expression. After 4 days, transfected cells were selected with 200 µg/mL Hygromycin (Calbiochem; 400051-1MU) in Schneider’s medium for ~1 month. *S2R*+-MT-Myc-BirA*G3 and *S2R*+-MT-Myc-BirA*G3-ER cell lines were isolated in the same manner.

For western analysis of biotinylated proteins, *S2R*+*-MT-sfGFP-TurboID* or *S2R*+*-MT-sfGFP-TurboID-ER* cells were grown in T25 flasks until near confluency (8 × 10^6^ cells/mL). 100 μM CuSO_4_ and 50 μM biotin (Sigma, B4639-1G) were added to induce gene expression and promote biotinylation, respectively. 24 h later, cells and culture media were collected separately. First, 1 mL of culture media was collected and centrifuged at 1000×*g* for 5 min to pellet remaining cells. The cell-free media was filtered through a 0.2 µm centrifuge filter (VWR, 525-0017) and concentrated with a 0.5 mL 3 kDa centrifuge filter (Sigma, UFC500324) at 14,000×*g*. To help remove excess biotin from the sample, 500 µL of PBS was added to the retentate on the column, and the sample was centrifuged again. This biotin wash was performed 2×. The retentate was recovered (~25 µL) and protein concentration determined by BCA assay (Thermo Fisher, 23227). Second, the transfected cells were resuspended, centrifuged at 1000×*g* for 5 min, and the supernatant was discarded. The cell pellet was resuspended in PBS, and centrifugation was repeated; the supernatant was discarded, the cell pellet was lysed in RIPA buffer with protease inhibitors (Thermo Fisher, 89901, 87786), and the protein concentration was determined by BCA assay.

For small-scale streptavidin pulldown experiments involving transfection, 4 days after transfection in six-well plates, 100 μM CuSO4 and 50 μM biotin (Sigma, B4639-1G) were added to induce gene expression and promote biotinylation, respectively. For small-scale streptavidin pulldown experiments involving stable cell lines, cells were grown to ~8 × 10^6^ cells/mL in six-well plates, and 100 μM CuSO_4_ and 50 μM biotin were added to induce gene expression and promote biotinylation, respectively. 24 h later, 1 mL of culture media was collected and centrifuged at 1000×*g* for 5 min to pellet remaining cells. The cell-free media was filtered through a 0.2 µm centrifuge filter (VWR, 525-0017) and concentrated with 0.5 mL 3 kDa centrifuge filter (Sigma, UFC500324) at 14,000×*g*. To help remove excess biotin from the sample, 500 µL of PBS was added to the retentate on the column, and the sample was centrifuged again. This biotin wash was performed 2×. The retentate was recovered (~25 µL) and protein concentration determined by BCA assay (Thermo Fisher, 23227).

For large-scale streptavidin pulldown experiments, *S2R*+*-MT-sfGFP-TurboID* and *S2R*+*-MT-sfGFP-TurboID-ER* cells were grown in 182 cm^2^ cell culture flasks (hold 30 mL volume) until nearly confluent (8 × 10^6^ cells/mL). 100 μM CuSO_4_ and 50 μM biotin (Sigma, B4639-1G) were added to induce gene expression and promote biotinylation, respectively. 24 h later, 30 mL of culture media was collected and centrifuged at 1000×*g* for 5 min to pellet remaining cells. The cell-free media was filtered through a 0.2 µm centrifuge filter (Thomas Scientific, 229710) and concentrated with 15 mL 3 kDa centrifuge filter (Sigma, UFC900324) at 4000×*g*. To help remove excess biotin from the sample, 15 mL of PBS was added to the retentate on the column, and the sample was centrifuged again. This biotin wash was performed 2×. The retentate was recovered (200–500 µL) and protein concentration determined by BCA assay (Thermo Fisher, 23227).

### Antibody staining and imaging

S2R+ cells in 384-well plates were fixed in 4% paraformaldehyde (Electron Microscopy Sciences, 15710) for 30 min, washed with PBS with 0.1% Triton X-100 (PBT) three times for 5 min each, stained with primary antibodies overnight at 4˚ °C, washed with PBT, stained with secondary antibodies for 2 h at 25˚ °C, and washed with PBS. Plates were imaged on an IN Cell Analyzer 6000 (GE Healthcare) using a ×60 objective. Images were processed using Fiji (v2.14) software.

Antibodies used:

mouse anti-Cnx99A (1:10, DSHB, Cnx99A 6-2-1)

chicken anti-GFP (1:1000, AVES labs GFP1020)

streptavidin-647 (1:500, Thermo Fisher, S21374)

goat anti-chicken 488 (1:500, Thermo Fisher, A-11039)

donkey anti-mouse 488 (1:500, Molecular Probes A-21202)

rabbit anti-myc (1:250, Cell Signaling 71D10)

donkey anti-rabbit 488 (1:500, Molecular Probes A21206)

Whole larvae fluorescence was imaged by immobilizing 3rd instar larvae in a drop of PBS in a glass-well dish on a slide warmer (set to maximum) for 5 min. Larvae were imaged on a Zeiss Axio Zoom V16 fluorescence microscope. Tissue-Gal4, UAS-GFP-TurboID-ER lines were crossed with *UAS-6x-GFP* to boost the fluorescence signal.

For imaging larval tissues, wandering 3rd instar larvae were dissected in PBS, and tissues were fixed in 4% paraformaldehyde for 20 min. Tissues were permeabilized in PBT, blocked for 1 h in 5% normal goat serum (S-1000, Vector Labs) at room temperature, and incubated with anti-GFP antibody overnight at 4 °C, washed with PBT, incubated with anti-chicken 488 and phalloidin and DAPI for 4 h at room temperature, washed with PBT and PBS, and incubated in mounting media (90% glycerol + 10% PBS) overnight at 4 °C. Stained tissues were mounted on a glass slide under a coverslip using Vectashield (H-1000, Vector Laboratories Inc). Images of stained tissues were acquired on a Zeiss 780 confocal microscope. Images were processed using Fiji (v2.14) software.

Antibodies/stains used:

chicken anti-GFP (1:1000, AVES labs GFP1020)

goat anti-chicken 488 (1:500, Thermo Fisher, A-11039)

phalloidin-660 (1:40, Invitrogen, A22285)

DAPI (1:1000, Invitrogen, D1306)

### Western blotting and SDS–PAGE gel staining

Protein or cell samples were denatured in 2× SDS Sample buffer (100 mM Tris–Cl, pH 6.8, 4% SDS, 0.2% bromophenol blue, 20% glycerol, 0.58 M β-mercaptoethanol) by boiling for 10 min. Denatured proteins and Pageruler Prestained Protein Ladder (Thermo Fisher Scientific 26616) were loaded into 4–20% Mini-PROTEAN TGX gels (Biorad 4561096) using running buffer (25 mM Tris, 192 mM glycine, 0.1% SDS, pH 8.3). Equal amounts of denatured proteins were loaded per lane unless otherwise noted. Gels were run at 100–200 V in a Mini-PROTEAN Tetra Vertical Electrophoresis Cell (Biorad 1658004).

Denatured proteins loaded per lane:

Fig. 4e—3.6 µg

Supplementary Fig. 3a and b—15 µg

Supplementary Fig. 3c and d—15 µg

Supplementary Fig. 4—Pulldown: Not determined due to sample buffer elution. 20 µL loaded.

Supplementary Fig. 4—Input: 5.4 µg

Supplementary Fig. 5a—Not determined due to sample buffer elution. 20 µL loaded.

Supplementary Fig. 5b—Input: 5.4 µg

Supplementary Fig. 5b—Flowthrough: same volume as input

Supplementary Fig. 5b—Pulldown: Not determined due to sample buffer elution. 20 µL loaded.

Supplementary Fig. 6c and d—3.6 µg

Supplementary Fig. 6e—Dilution series, µg loaded indicated on the figure.

Supplementary Fig. 7c—Not determined due to sample buffer elution. 20 µL loaded.

Supplementary Fig. 7d—20 µg

Supplementary Fig. 8a—Not determined due to sample buffer elution. 20 µL loaded.

Supplementary Fig. 8b—Not determined due to sample buffer elution. 20 µL loaded.

Supplementary Fig. 21—3.6 µg

For large-scale pulldown/M.S. involving excised Coomassie-stained gel bands, 5/6th of the elution was run on a gel, and individual protein bands were excised, whereas 1/6th of the elution was run 1 cm into the gel, and the entire lane was excised.

For western blotting, proteins were transferred to Immobilon-FL PVDF (Millipore IPFL00010) in transfer buffer (25 mM Tris, 192 mM glycine) using a Trans-Blot Turbo Transfer System (Biorad 1704150) (Standard SD program). When applicable, blots were stained with Ponceau S Solution (Sigma, P7170) for 5 min, washed with ddH_2_O, imaged on a ChemiDoc MP Imaging System (BioRad), and destained in 0.1 M NaOH. Blots were incubated in TBST (1 × TBS + 0.1% Tween20) for 20 min on an orbital shaker, blocked in 3% BSA (Sigma, A3912) in TBST for 1 h at room temperature, and incubated with primary antibody diluted in blocking solution overnight at 4 °C. Blots were washed with TBST and incubated in secondary antibody in blocking solution for 4 h at room temperature. Blots were washed in TBST before detection of proteins. HRP-conjugated streptavidin or secondary antibodies were visualized using ECL (34580, ThermoFisher). Fluorophore-conjugated secondary antibodies were visualized using fluorescence. Blots were imaged on a ChemiDoc MP Imaging System (BioRad). Target proteins were reprobed by incubating blots with stripping buffer (Thermo Scientific 46430) following the manufacturer’s instructions, re-blocked in 3% BSA in TBST, and incubated with primary antibody overnight as described.

Antibodies used:

Streptavidin-HRP (0.3 µg/mL, Thermo Fisher, S911)

Rhodamine Anti-Actin (1:10,000, Biorad 12004163)

rabbit anti-GFP (1:5000, Invitrogen a6455)

goat anti-rabbit 800 (1:5000, Thermo Fisher, A32730)

chicken anti-HA (1:5000, Aves, ET-HA100)

goat anti-chicken-HRP (1:1000, Sigma, SAB3700199)

rabbit anti-LSP-1γ (1:5000)^103^

rabbit anti-myc (1:2000, Cell Signaling 71D10)

donkey anti-rabbit-HRP (1:3000, Amersham NA934)

SDS–PAGE gels were stained using silver staining or Coomassie staining. For silver-stained gels, we used the Pierce Silver Stain Kit (Thermo Fisher, 24612). For Coomassie staining, SDS–PAGE gels were fixed in fixing solution (40% Methanol/10% acetic acid) for 1 h, stained in 0.25% Coomassie Brilliant Blue R-250 in fixing solution for 4 h, and destained in fixing solution. Stained gels were imaged on a ChemiDoc MP Imaging System. Gel lanes and bands were cut out using a clean razor blade.

### Streptavidin pulldowns

Streptavidin pulldowns were performed as previously^6^ with modifications noted.

For small-scale pulldowns (i.e., for western analysis), 360 µg of sample was resuspended in 500 µL RIPA buffer + protease inhibitors (Thermo Fisher, 89901, 87786). 7.5 µL of resuspended sample was removed as input and boiled with 7.5 µL 4× SDS Sample Buffer (200 mM Tris–Cl pH 6.8, 8% SDS, 0.4% bromophenol blue, 40% glycerol, 1.16 M β-mercaptoethanol). 30 µL of magnetic streptavidin beads (Pierce 88817) were washed in RIPA buffer using a magnetic rack (Bio-Rad, 1614916), combined with the sample, and allowed to bind the beads for 1 h at room temperature. Note that Supplementary Fig. 5b used the small-scale pulldown protocol.

For large-scale pulldowns (i.e., for LC–MS/MS), 3 mg of sample was diluted with RIPA buffer + 1× protease inhibitors to 500 µL total volume. 5 µL of this sample input was retained and boiled with 4× sample buffer for later SDS–PAGE analysis. 250 µL of magnetic streptavidin beads were washed in RIPA buffer using a magnetic rack, combined with the sample, and allowed to bind the beads for 1 h at room temperature. Note that Supplementary Fig. 5a used the large-scale pulldown protocol.

Sample + beads were incubated rocking for one hour at room temperature. Beads were washed sequentially to remove nonspecific binders (1 mL for each wash): twice with RIPA buffer, once with 1 M KCl, once with 0.1 M Na_2_CO_3_, once with 2 M urea in 10 mM Tris–HCl (pH 8.0), and twice with RIPA buffer.

To reduce non-specific binding to beads, we tested alternative urea wash steps, which ranged from 2, 4, 6, or 8 M urea and 0%, 1%, or 2% SDS. We used 4 M urea + 2% SDS in 10 mM Tris–HCl pH 8.0, for pulldowns for the full 10-tissue secretome map using TMT labeling and LC–MS/MS.

For elution of biotinylated proteins from beads to run on an SDS–PAGE gel, beads were boiled in 30 µL of 3× SDS Sample buffer with biotin and DTT (150 mM Tris–Cl pH 6.8, 6% SDS, 0.3% bromophenol blue, 30% glycerol, 0.87 M β-mercaptoethanol, 2 mM biotin, 20 mM DTT) for 10 min.

For submission of biotinylated proteins for on-bead digestion, we suspended washed beads in 500 µL in PBS (for LC–MS/MS using spectral counting) or RIPA buffer (for LC–MS/MS using TMT labeling).

### Protein localization prediction and searching

Uniprot entry #s from LC–MS/MS data were input into the Uniprot Retrieve/ID mapping tool (https://www.uniprot.org/id-mapping), and exported fields “Subcellular Location”, “Topological domain”, “Transmembrane”, and “Signal”. For the “Subcellular Location” and “Topological domain” fields, secreted and/or transmembrane proteins were identified using the keywords “secreted” or “extracellular”. For the “Transmembrane” and “Signal” fields, secreted and/or transmembrane proteins were identified by the presence of a predicted transmembrane domain or signal sequence, respectively.

Uniprot entry #s were converted to FlyBase gene names (FBgns), input into the Flybase Batch Download tool (http://flybase.org/batchdownload), and exported the field “Gene Ontology(GO): Cellular Component”. Secreted and/or transmembrane proteins were identified using keywords “extracellular space; GO:0005615” or “extracellular region; GO:0005576”.

FBgns were compared with predictions of “Secreted proteins” and “Trans-membrane proteins”, the Gene List Annotation for *Drosophila* (GLAD) tool (v1.0) (https://www.flyrnai.org/tools/glad/web/)^52^.

FBgns were converted to Flybase polypeptides (FBpp) for all protein isoforms (http://flybase.org/download/sequence/batch) and used as input for SignalP 6.0^104^, SignalP 5.0^105^, TMHMM 2.0^106^, DeepTMHMM 1.0^107^, and DeepLoc 2.0^108^. In all cases, the default threshold of each software package was used to classify whether a protein had a secretion signal sequence (SignalP), transmembrane domain (TMHMM and DeepTMHMM), or “Extracellular” localization (DeepLoc). To maximize identifying domains suggestive of secretion, any single protein isoform that scored as secreted, transmembrane, or extracellular was used as a representative of the gene encoding all protein isoforms.

We classified proteins as either “secreted” or “transmembrane” by comparing localization predictions from Uniprot, Flybase, GLAD, and bioinformatic prediction software. For example, proteins that contained a secretion signal sequence with no transmembrane domain were classified as “secreted”, and proteins that contained a transmembrane domain as “transmembrane”.

535 tissue-secreted proteins were designated “found in or annotated as in hemolymph” by combined searching of the term “hemolymph” (FBbt:00005061) on Flybase, Uniprot subcellular location “hemolymph”, presence in a previous 3rd instar hemolymph proteomic dataset^20^, and presence in this study’s label-free 3rd instar hemolymph proteomic dataset. 535 tissue-secreted proteins were designated “known function in hemolymph” and sub-functional categories (e.g., Hormone/growth factor) by manual curation using Flybase reports and literature searching.

Pie charts were made using Prism (v10.4.1) (Graphpad).

### Homology and disease association searching

Fly genes with at least one human homolog were identified using the DIOPT Ortholog Finder (https://www.flyrnai.org/diopt) version 10.0^109^ by searching Fly → Human using Flybase gene numbers (FBgns) corresponding to the 535 tissue-specific proteins. Homologs were defined as having a DIOPT score of 3 or higher.

Fly genes that correspond to conserved secreted and/or plasma proteins were identified by querying DIOPT Human → Fly using a list of human secreted proteins and a list of human plasma proteins from the Human Protein Atlas^2^. Homologs were defined as having a DIOPT score of 3 or higher.

Fly genes with at least one human homolog with at least one OMIM phenotype were identified using FlyBase (dmel_human_orthologs_disease_fb_2025_05.tsv).

### Bioinformatic definition of small and large proteins

The *Drosophila* proteome containing 30,836 protein isoforms was downloaded from Flybase (dmel-all-translation-r6.66.fasta). A Python (v3.13.2) script was used to extract FBgn IDs encoding proteins 100aa or smaller for all protein isoforms (847 in total). A separate Python script was used to extract FBgn IDs encoding any protein isoforms larger than 100aa (13,139 in total).

### *Drosophila* genetics

Flies were maintained on standard fly food at 25 °C. All in vivo experiments were performed using wandering third instar *Drosophila melanogaster* larvae (5 days after egg laying), unless otherwise indicated. Larvae were not sexed except where specified.

Fly stocks were obtained from the Perrimon lab collection, stock centers, donating labs, or generated in this study (see below).

Perrimon Lab stocks:

*Lpp-Gal4* (Chr. III)

*Hml-Gal4* (Chr. X)

*yw; nos-Cas9 attp40* (Chr. II)

*yw; nos-Cas9 attp2* (Chr. III)

Bloomington *Drosophila* Stock Center:

*w*^*1118*^ (5905)

*repo-Gal4* (7415)

*promE-Gal4* (65405)

*elav-Gal4* (8760)

*MS10906-Gal4* (8860)

*mef2-Gal4* (27390)

w[1118]; Herm[3xP3-ECFP,alphatub-piggyBacK10]M6; MKRS/TM6B, Tb[1]) (32070)

yv,P[y[+t7.7] = nos-phiC31\int.NLS]X;P [y[+t7.7] = CaryP]attP40 (25709)

P[y[+t7.7] = nos-phiC31\int.NLS]X, y(1) sc(1) v(1) sev(21); P[y[+t7.7] = CaryP]attP2 (25710)

*UAS-6x-GFP* (52262)

Kyoto Stock Center:

*btl-Gal4* (105276)

*myo1a-Gal4* (112001)

Other sources:

*4xHand-Gal4* ^110^

This study (BDSC stock # indicated):

*w; UAS-sfGFP-TurboID-ER Chr.II* (BDSC 606643)

*w;; UAS-sfGFP-TurboID-ER Chr.III* (BDSC 606644)

*w;; Mef2-Gal4, UAS-sfGFP-TurboID-ER/TM3, Sb* (BDSC 606645)

*w, MS1096-Gal4; UAS-sfGFP-TurboID-ER* (BDSC 606646)

*w;; 4xHand-Gal4, UAS-sfGFP-TurboID-ER/CyO* (BDSC 606647)

*w, hml-Gal4; UAS-sfGFP-TurboID-ER* (BDSC 606648)

*w;; promE-Gal4, UAS-sfGFP-TurboID-ER/TM3* (BDSC 606649)

*w; Myola-Gal4, UAS-sfGFP-TurboID-ER/CyO* (BDSC 606650)

*w;; lpp-Gal4, UAS-sfGFP-TurboID-ER/TM3* (BDSC 606651)

*w;; btl-Gal4, UAS-sfGFP-TurboID-ER/TM3* (BDSC 606652)

*w;; elav-Gal4, UAS-sfGFP-TurboID-ER/TM3* (BDSC 606653)

*w;; repo-Gal4, UAS-sfGFP-TurboID-ER/TM3* (BDSC 606695)

*yw;; UAS-CG6867-sfGFP* (BDSC 606654)

See Supplementary Data 7 for information on knock-in fly strains.

Transgenic flies were made by injecting an *attB*-containing plasmid at 200 ng/µL into *nos-PhiC31* integrase-expressing embryos that contain an *attP* landing site (*attP40* or *attP2*). The plasmid contains a *white+* marker gene for positive selection. Injected adults were outcrossed to *white-* balancer chromosome lines to isolate transgenic founder flies with *white*+(red) eyes and generate balanced stocks.

Fly strains stably carrying both *Gal4* and *UAS-sfGFP-TurboID-ER* transgenes were generated by genetic crosses between *Gal4* strains with *UAS-sfGFP-TurboID-ER*, using recombination in cases where both transgenes were on the same chromosome. Isolating flies carrying both transgenes was aided by manually selecting larvae or adults that contained GFP fluorescence, using a Zeiss Axio Zoom V16 fluorescence microscope.

Knock-in flies were made by co-injecting the sgRNA plasmid and donor plasmid (500 ng/µL total) into *nos-Cas9* embryos. We used a 5:1 ratio of donor:sgRNA plasmid. *nos-Cas9 attp40* was used for targeting genes on Chr. III and *nos-Cas9 attp2* was used for targeting genes on Chr. X, II, and IV. Injected embryos were raised to adult flies, which were outcrossed to *yw*, and adult progeny were screened for RFP+ fluorescent eyes on a Zeiss Axio Zoom V16 fluorescence microscope. Injected males were set up as single male crosses to *yw* females, and injected females were set up as pooled crosses with *yw* males. We recorded the number of injected male crosses or pooled female crosses that gave any RFP+ progeny. We also recorded the number of injected male crosses or pooled female crosses that gave any RFP+ progeny that had small eyes. Candidate knock-in flies (RFP+, normal-size eyes) were outcrossed to a balancer chromosome to establish a stock, while also collecting RFP+ flies for PCR genotyping. Genomic DNA was isolated from 25 adult RFP+ flies using the Quick-DNA miniprep kit (Zymo, D3024). Candidate RFP+ lines were genotyped by PCR by amplifying and sequencing the region flanking both homology arms. Primers that amplify the homology arm region were designed such that one primer binds to the genomic sequence distal to the homology arm and the other primer binds to the insert sequence. This strategy ensures that the target locus is amplified. Amplified homology arm regions were Sanger sequenced to confirm the correct knock-in event. The overall knock-in success rate was calculated as the number of targeting constructs (i.e., unique injection mixes) vs. the number of successful knock-in fly strains.

To generate scarless sfGFP-tagged knock-in lines, the *3xP3-RFP* marker was removed by crossing pre-excision alleles to a PBac transposase strain (*Herm[3xP3-ECFP,alphatub-piggyBacK10]M6*), outcrossing F1 progeny to a balancer strain, and selecting F2 progeny that lack the RFP+ eye marker. Candidate sfGFP-tagged alleles were genotyped as described above, using one primer that binds the *GFP* sequence and one that binds distal to the homology arm.

### Larval hemolymph isolation

#### Large-scale collection (for streptavidin pulldowns)

To synchronize developmental timing and prevent overcrowding of larvae, we allowed adult flies to lay eggs on food with yeast pellets for 6–8 h, and progeny were raised at 25 °C. Four days later, larvae were floated with 20% Glycerol (Sigma, G7757-1GA) and ~200 were transferred to fresh fly food containing excess biotin (Sigma, B4639-1G). Biotin fly food was prepared by mixing a biotin stock solution (1 mM Biotin in H_2_O) 1:10 with microwaved liquid fly food (final 100 µM Biotin). Transferred larvae were raised on biotin fly food at 25 °C for 24 h. Five-day-old larvae were floated with 20% Glycerol and washed using a mesh basket (Genesee Scientific, 46-102, 57-107, 57-101) to remove excess biotin fly food stuck to larvae. 1 g washed larvae (~400 larvae) were transferred to a glass dish (Carolina Biological Supply 742300) on ice. Larvae were washed with ice-cold PBS (Gibco, 10010023) in the glass dish until no food particles remained, after which larvae were resuspended in 1000 µL ice cold PBS.

Larvae were bled using microscissors (World Precision Instruments, 501778) to nick the posterior cuticle. Larval bleeding for each sample (~400 larvae) was performed within ~30 min while keeping the glass dish on ice. Larval blood in PBS was filtered through a 100 µm cell strainer (Falcon, 352360) to remove carcasses, using ice-cold PBS to gently rinse the glass dish and carcasses in the cell strainer, finishing with no more than 15 mL diluted crude larval blood in PBS per sample (~400 larvae). Crude diluted blood was centrifuged at 250×*g* for 10 min, the supernatant was passed through a 0.8 µm/0.2 µm filter (Pall Corporation 4187) using a 30 mL syringe (BD 309650), and the flowthrough was centrifuged through a 15 mL 3 kDa centrifugal filter unit (Millipore UFC9003) at 4000×*g*. The resulting ~250 µL purified, concentrated blood was removed from the centrifugal filter unit and diluted with 500 µL RIPA (Thermo Fisher, 89901) + 1.5× protease inhibitors (Thermo Fisher, 87786). Protein concentration was determined using a BCA assay (Thermo Fisher, 23227). The remaining sample was snap-frozen in liquid nitrogen and stored at −80 °C.

#### Small-scale collection (for non-pulldowns, for western blotting)

Twenty 3rd instar larvae were washed in cold PBS and transferred to a paper towel to dry for 1 min. Larvae were transferred to a 1” × 1” square of parafilm. Larvae were bled using microscissors (World Precision Instruments, 501778) to nick the posterior cuticle, and the carcasses were pushed together to pool the bled hemolymph. 2 µL of raw hemolymph was removed and transferred to 10 µL of cold PBS. The diluted hemolymph was centrifuged at 1000×*g* for 10 min at 4 °C to pellet cells and tissue. 10 µL of supernatant was transferred to a new tube containing 10 µL 4× sample buffer and boiled for 10 min.

### LC–MS/MS for pilot TurboID-ER labeling experiments in S2R+ cells and hemolymph

Protein samples were reduced with 55 mM DTT, alkylated with 10 mM iodoacetamide (Sigma-Aldrich), and digested overnight with TPCK-modified trypsin/LysC (Promega) at pH = 8.3. Peptides were immunoprecipitated with a pTyr antibody, dried out in a SpeedVac, resuspended in 10 μL of 1% Acetonitrile/98.9% Water, 0.1% Formic acid. 3 μL of the digested protein samples were analyzed in positive ion mode via microcapillary tandem mass spectrometry (LC–MS/MS) using a high-resolution hybrid QExactive HF Orbitrap Mass Spectrometer (Thermo Fisher Scientific) via HCD with data-dependent analysis (DDA) with 1 MS1 scan followed by 8 MS2 scans per cycle (Top 8). Peptides were delivered and separated using an EASY-nLC1000 nanoflow UPLC (Thermo Fisher Scientific) at 300 nL/min using self-packed 15 cm length × 75 μm i.d. C18 fritted microcapillary columns. Solvent gradient conditions were 90 min from 3% B buffer to 38% B (B buffer: 100% acetonitrile; A buffer: 0.9% acetonitrile/0.1% formic acid/99.0% water). MS/MS spectra were analyzed using Mascot Version 2.7 (Matrix Science) by searching the reversed and concatenated *Drosophila* protein Database: the Drosophila_20180328 database (unknown version, 13767 entries) with a parent ion tolerance of 18 ppm and fragment ion tolerance of 0.05 Da. Carbamidomethylation of cysteine (+57.0293 Da) was specified as a fixed modification, and oxidation of Methionine (+15.9949 Da), deamidation of Asparagine/Glutamine (+0.984 Da) as variable modifications. Results were imported into Scaffold Q+S 5.0 software (Proteome Software, Inc.) with a peptide threshold of ~70%, protein threshold of 95%, resulting in a peptide false discovery rate (FDR) of ~1%.

For tissue-labeling experiments involving whole-lane in-gel digestion, tissue-enriched proteins were calculated as >3 spectral counts from the tissue and at least 0.25 enrichment (# spectral counts from specific tissue/# spectral counts from all tissues).

### Tissue secretome map: On-bead trypsin digestion of biotinylated proteins

On-bead trypsin digestion was performed as described previously^6^. The biotin-labeled proteins bound to magnetic beads were further washed to remove detergent traces. Magnetic beads were immobilized and washed twice with 200 μL of 50 mM Tris–HCl buffer (pH 7.5), followed by two washes with 2 M urea/50 mM Tris (pH 7.5) buffer. A partial trypsin digestion was performed to release proteins from the beads by using 80 μL of 2 M urea/50 mM Tris containing 1 mM DTT and 0.4 μg trypsin (Mass Spectrometry Grade, Promega, catalog #V5280) for 1 h at 25 °C. Magnetic beads were immobilized and the supernatant containing partially digested proteins was transferred to a fresh tube. The beads were washed twice with 60 μL of 2 M urea/50 mM Tris buffer (pH 7.5), and the washes were combined with the on-bead digest supernatant. Proteins were reduced with 4 mM DTT for 30 min at 25 °C with shaking, followed by alkylation with 10 mM iodoacetamide for 45 min in the dark at 25 °C. Proteins were completely digested by adding 0.5 μg of trypsin and incubating overnight at 25 °C with shaking. Following the overnight incubation, samples were acidified to 1% formic acid (FA) and desalted using stage tips containing 2× C18 disks (Empore, Fisher Scientific catalog # 13-110-019) as described next. The stage tip column was conditioned with 1 × 100 μL methanol, 1 × 100 μL 50% acetonitrile (ACN)/1% FA, and 2 × 100 μL 0.1% FA washes. Acidified peptides were bound to the column, washed with 2 × 100 μL 0.1% FA, and eluted with 50 μL 50% ACN/0.1% FA. Eluted peptides were dried using a vacuum concentrator.

### Tissue secretome map: TMT-labeling and fractionation of peptides

Desalted peptides were labeled with 10-plex TMT reagents (ThermoFisher Scientific, catalog #9406) and fractionated as described previously^6^. Dried peptides were reconstituted in 100 μL of 50 mM HEPES, labeled using 0.8 mg of TMT reagent in 41 μL of anhydrous acetonitrile for 1 h at room temperature. The TMT reactions were quenched with 8 μL of 5% hydroxylamine at room temperature for 15 min with shaking, and the labeled peptides were desalted on C18 stage tips as described above. Peptides were fractionated by basic reverse phase using styrenedivinylbenzene-reverse phase sulfonate (SDB-RPS, Empore, Fisher Scientific catalog # 13-110-023) material in stage tip columns. Two-disc punches were packed in a tip and equilibrated with 50 μL methanol, 50 μL 50% CAN/0.1% FA, and 2 × 75 μL 0.1% FA washes. Peptides were reconstituted in 0.1% FA and loaded into the column, followed by conditioning with 25 μL of 20 mM ammonium formate (AF). Next, peptides were sequentially eluted into 6 × 100 μL fractions with 20 mM AF and varying concentrations of ACN: 5%, 10%, 15%, 20%, 30%, and 55%. The six peptide fractions were dried by vacuum centrifugation.

### Tissue secretome map: liquid chromatography and mass spectrometry analysis

Desalted peptides were resuspended in 9 μL of 3% ACN/0.1% FA and analyzed by online nanoflow liquid chromatography tandem mass spectrometry (LC–MS/MS) using a Proxeon Easy-nLC 1200 coupled to a Q Exactive HF-X Hybrid Quadrupole-Orbitrap Mass Spectrometer (ThermoFisher Scientific) set to positive ion mode acquisition. Four μL of sample from each fraction were injected in a capillary column (360 × 75 µm, 50 °C) containing an integrated emitter tip packed to a length of approximately 25 cm with ReproSil-Pur C18-AQ 1.9 μm beads (Dr. Maisch GmbH, catalog # r119.aq). Chromatography was performed with a 110 min gradient of solvent A (3% ACN/0.1% FA) and solvent B (90% ACN/0.1% FA). The gradient profile, described as min:% solvent B, was 0:2, 1:6, 85:30, 94:60, 95:90, 100:90, 101:50, 110:50. Ion acquisition was performed in data-dependent MS2 (ddMS2) mode with the following relevant parameters: MS1 acquisition (60,000 resolution, 3E6 AGC target, 10 ms max injection time) and MS2 acquisition (Loop count = 20, 0.7*m*/*z* isolation window, 31 NCE, 45,000 resolution, 5E4 AGC target, 105 ms max injection time, 1E4 intensity threshold, 15 s dynamic exclusion, and charge exclusion for unassigned, 1 and >6).

### Tissue secretome map: proteomic data analysis

Collected RAW LC–MS/MS data were analyzed using the Spectrum Mill software package v6.1 pre-release (Agilent Technologies). MS2 spectra were extracted from RAW files and merged if originating from the same precursor, or within a retention time window of ±60 s and *m*/*z* range of ±1.4, followed by filtering for precursor mass range of 750–6000 Da and sequence tag length > 0. MS/MS search was performed against a custom concatenated FASTA database containing (1) the *Drosophila melanogaster* UniProt (www.uniprot.org) protein database (UP000000803) downloaded in September 2018, (2) a list of common non-human and non-fly contaminants, and (3) the GFP-TurboID-KDEL protein sequence (Supplementary Note 5). Search parameters were set to “Trypsin allow P”, <5 missed cleavages, fixed modifications (cysteine carbamidomethylation and TMT10 on N-term and internal lysine), and variable modifications (oxidized methionine, acetylation of the protein N-terminus, pyroglutamic acid on N-term Q, pyro carbamidomethyl on N-term C, and NHS-Biotin on K). Matching criteria included a 30% minimum matched peak intensity and a precursor and product mass tolerance of ±20 ppm. Peptide-level matches were validated if found to be below the 1.0% false discovery rate (FDR) threshold and within a precursor charge range of 2–5. TMT reporter ion intensities were corrected for isotopic impurities in the Spectrum Mill protein/peptide summary module using the afRICA correction method, which implements determinant calculations according to Cramer’s Rule^111^ and correction factors obtained from the reagent manufacturer’s certificate of analysis (Thermo Fisher, cat. # 90406, lot # UA280170).

A protein-centric summary containing TMT channel intensities was exported from Spectrum Mill for downstream analysis in the R (v4.1.0) environment for statistical computing. Proteins were filtered to remove those that do not originate from *Drosophila* or the TurboID protein. TMT quantitative values were removed if <2 peptides were quantified, or the protein score was below 20 for the plex to avoid low-quality quantification. In addition, proteins with a PaxDB (v5.0) score^112^ >1500 ppm are removed to remove extremely abundant contaminant proteins (e.g., ribosomes). Sample agreement and outliers were inspected by principal component analysis. Those with large distances in PC1 and PC2 from the average of the experimental group were flagged and manually inspected. Two outlier samples were detected and removed for downstream analysis. Downstream analysis was performed on each plex and tissue group separately. First, log TMT ratios were calculated by dividing by the median of the negative control samples within that plex and log2-transformed. Two-sample moderated *T*-tests were performed using the limma package (v3.48.3) in R with correction for multiple testing by calculating local FDR^113^, producing protein log_2_ fold-changes (logFC) and *q*-values for contrasts between samples expressing TurboID and controls. Missing values were not imputed, therefore logFC were not calculated for a protein within a specific plex and tissue group if (a) the protein had missing values across all replicates from the negative control samples in that plex or (b) the protein had missing values across all the experimental samples from the tissue group within the plex. The separate list of proteins with “infinite” logFC (Supplementary Data 3c) was defined as (a) having no reporter intensity for all negative control replicates in all plexes and (b) having reporter intensity values for all three replicates for a given tissue in a plex. Secretion signal sequences were determined by the Uniprot (www.uniprot.org) “Signal” field annotation. Correlation analysis was performed using Pearson’s correlation by comparing TMT ratios between all samples in a plex (Supplementary Fig. 11). Pearson’s correlation was also performed between the TMT logFC from the tissue secretome map and the log10 spectral counts from the label-free blood proteome dataset (see the “Methods” section and Supplementary Fig. 12).

### Tissue secretome map: Defining high-confidence tissue secreted factors

High-confidence proteins were defined by using an empirical false-discovery rate criterion using a predefined list of secreted positive control (PC) proteins and non-secreted negative controls (NC) (Supplementary Data 3a). The positive control list was obtained from ref. ^18^, which contains the following: (1) fly receptors and secreted factors and (2) fly orthologs of human receptors, secreted proteins, and blood plasma proteins. The negative control list contains proteins from the Uniprot database with “cytoplasm”, “nucleus”, or “mitochondria” subcellular location annotations, and proteins from the GLAD database (https://fgr.hms.harvard.edu/glad)^52^ with mitochondrial or transcription factor/DNA-binding annotation. Proteins were sorted in descending order using the logFC, and the empirical false discovery rate (FDR = FP/(FP + TP)) was calculated at each logFC threshold. False positives (FP) were defined as proteins in the NC list with a logFC equal to or greater than the threshold. True positives (TP) were defined as proteins in the PC list with a logFC equal to or greater than the threshold. The empirical FDR was plotted as a function of the logFC and inspected to manually select a point that minimizes logFC while preserving a small FDR. Four redundant protein isoforms were removed, which were selected in a way so as not to lose tissue-of-origin information. We removed B7Z0B3 (*CG10359*) from muscle, because B7Z0B2 (*CG10359*) was identified from muscle/heart/wingdisc/neurons. We removed B7YZF1 (*CG42336*) from fat, because A1Z8I8 (*CG42336*) was identified from fat/gut. We removed D3PK81 (*CtsF*) from muscle, because Q9VN93 (*CtsF*) was identified from muscle, is a longer isoform, and has a “reviewed” status at Uniprot. We removed Q8IQU1 (*verm*) from the trachea, because E1JI40 (*verm*) was identified from trachea/gut/heart/wingdisc.

### Blood proteome: Label-free proteomic analysis of whole blood

Whole blood from *Drosophila* larvae (250 µL at 30 mg/mL) was digested using the S-Trap Midi Spin Column (Protifi, catalog # C02-midi-10) as per the manufacturer's recommendation. Blood was diluted to 500 µL in SDS solubilization buffer (5% SDS, 50 mM TEAB, pH 7.5), clarified by centrifugation, and acidified using 50 µL of 12% phosphoric acid. The S-trap column was conditioned with 3.3 mL S-Trap buffer (90% MeOH, 100 mM TEAB, pH 7.1), samples were loaded by centrifugation, and washed with 3× 3 mL S-Trap buffer. Digestion was performed overnight with 1:50 wt:wt trypsin and 1:50 wt:wt LysC in 50 mM TEAB. Peptides were eluted sequentially with 500 µL each of 50 mM TEAB, 0.2% FA, and 0.2%FA/50%ACN. A total of 100 µg was lyophilized, resuspended in buffer A (5 mM ammonium formate, pH 10, in 2% acetonitrile), and fractionated by high pH reversed phase separation using a 3.5 µm Agilent Zorbax 300 Extend-C18 column (2.1 mm ID × 250 mm length). Samples were loaded onto the column and separated at a flow rate of 1 mL/min in a 96 min gradient with the following concentrations of solvent B (5 mM ammonium formate, pH 10.0 in 90% vol/vol MeCN): 16% solvent B at 13 min, 40% solvent B at 73 min, 44% solvent B at 77 min, 60% solvent B at 82 min, and 60% solvent B at 96 min. A total of 96 fractions were collected and concatenated non-sequentially into a final 24 fractions for proteomic analysis. Fractions were dried via vacuum centrifugation.

### Blood proteome: liquid chromatography and mass spectrometry analysis

Dried peptides were reconstituted at an estimated concentration of 0.5 µg/µL in 3% ACN/0.1% FA and analyzed by online nanoflow liquid chromatography tandem mass spectrometry (LC–MS/MS) using a Proxeon Easy-nLC 1200 coupled to a Q Exactive HF-X Hybrid Quadrupole-Orbitrap Mass Spectrometer (ThermoFisher Scientific). One μg of sample from each fraction was in a column setting similar to the one above. Chromatography was performed with a 110 min gradient of solvent A (3% ACN/0.1% FA) and solvent B (90% ACN/0.1% FA). The gradient profile, described as min: % solvent B, was 0:2, 1:6, 85:30, 94:60, 95:90, 100:90, 101:50, and 110:50. Ion acquisition was performed in data-dependent MS2 (ddMS2) mode with the following relevant parameters: MS1 acquisition (60,000 resolution, 3E6 AGC target, 10 ms max injection time) and MS2 acquisition (Loop count = 20, 0.7*m*/*z* isolation window, 28 NCE, 15,000 resolution, 1E5 AGC target, 50 ms max injection time, 1E4 intensity threshold, 7 s dynamic exclusion, and charge exclusion for unassigned, 1 and >6).

### Blood proteome: proteomic data analysis

Collected RAW LC–MS/MS data were analyzed using the Spectrum Mill software package v6.1 pre-release (Agilent Technologies). MS2 spectra were extracted from RAW files and merged if originating from the same precursor, or within a retention time window of ±60 s and *m*/*z* range of ±1.4, followed by filtering for precursor mass range of 750–6000 Da and sequence tag length > 0. MS/MS search was performed against a custom concatenated FASTA database containing (1) the *Drosophila melanogaster* UniProt protein database (UP000000803) downloaded in September 2018 and (2) a list of common non-human and non-fly contaminants. Search parameters were set to “Trypsin allow P”, <5 missed cleavages, fixed modifications (cysteine carbamidomethylation), and variable modifications (oxidized methionine, acetylation of the protein N-terminus, pyroglutamic acid on N-term Q, pyro carbamidomethyl on N-term C, and NHS-Biotin on K). Matching criteria included a 30% minimum matched peak intensity and a precursor and product mass tolerance of ±20 ppm. Peptide-level matches were validated if found to be below the 1.0% false discovery rate (FDR) threshold and within a precursor charge range of 2–5. Non-*Drosophila* proteins and proteins with a single unique peptide were removed from the results. The number of peptide-spectrum matches per protein (spectral counts) was used for semi-quantitative analysis and comparison to enrichments from the TMT-based tissue secretome map proteomics results.

### Gene set enrichment analysis, pathway enrichment analysis, and visualization

The gene set categories selected using PANGEA (v2beta)^37^ were GO slims, GLAD gene group annotation, pathway annotation from KEGG, DRSC, and FlyBase, expression annotation from AGR, as well as the tissue-specific gene expression from bulk as well as single-cell RNAseq datasets. The relevant gene sets from PANGEA results were selected, and the heatmaps were generated offline using MeV v4.7.4 (TM4 Microarray Software Suite). The scale on each plot was customized/optimized for visualization. The scale for both the heatmaps and bar graphs is −log_10_*P-*value, reflecting the significance of enrichment analysis. For analyzing connections with human disease, we separated queries using the Disease annotation AGR gene set category.

Pathway enrichment analysis was performed using g:Profiler (gprofiler2 v0.2.1)^41^ with the 535 high-confidence secreted proteins (Supplementary Data 3b) as input and all proteins identified across all plexes (Supplementary Data 3a) as a custom background. The pathway-enrichment results for all tissues were imported into Cytoscape v3.8.2 using the EnrichmentMap application for network visualization and cluster analysis with parameters *p*-value threshold of 1.0, FDR *Q*-value threshold of 0.05, and Jaccard Overlap Combined value of 0.375^114^.

### Bioinformatic prediction of tissue-secreted inter-organ proteins

The list of 535 tissue-secreted proteins was compared against annotated lists of “Matrisome” genes and “Major Signaling Pathway” genes using GLAD^52^, “DRSC PathON signaling pathway core components” and “FlyBase signaling pathway (experimental evidence)” using PANGEA^37^, and “XC domains likely involved in protein–protein interactions” and “XC domains mainly found in signaling molecules such as growth factors, hormones, and neuropeptides” using FlyXCDB^51^. Proteins overlapping with at least one of these gene lists were designated a potential inter-organ protein.

### Validating protein tissue-of-origin with transcriptome data

The processed *Drosophila* scRNA-seq datasets, such as FCA datasets and larval wing disc datasets, were obtained from the DRscDB database^61^, while tissue-specific bulk RNAseq datasets were obtained from the DGET database (v2.0.1)^115^. The tissue/cell type annotation from public datasets was manually matched to the tissue annotation of the secretome data then the genes expressed in each tissue/cell type were compared to the genes identified in the secretome data.

Overlap of our protein tissue secretome and our 3rd instar larval snRNA-seq data was determined by intersecting lists of FlyBase gene names (FBgn) between the two datasets. We represented LC–MS/MS-identified tissue secreted proteins as FBgns (Supplementary Data 3b) to enable matching FBgns from transcriptomic data. For validation of expression in a tissue, we counted the number of “Tissue X”-secreted proteins (Supplementary Data 3b) that are expressed in any “Tissue X” clusters. We defined expression as 1% or greater average expression OR 10% or greater percent expressed in Supplementary Data 5f–h. For validation of tissue-specific expression, we counted the number of “Tissue X”-specific secreted proteins (Supplementary Data 3b) that are marker genes in any “Tissue X” clusters. We defined a marker gene as 0.54 or greater avg_log2FC AND >0.05 p_val_adj. To calculate the combined number of tissue-secreted proteins that have a corresponding encoding gene expressed in the same tissue cluster(s), we counted the number of unique proteins expressed in at least one tissue (500) (Supplementary Data 6i). To calculate the combined number of tissue-specific-secreted proteins that have a corresponding encoding marker gene for the same tissue cluster(s), we counted the total number of cluster marker overlap proteins (105) (Supplementary Data 6j).

snRNA-seq data were processed using FlyPhoneDB2 (v1.0)^62^ by uploading the Seurat object and using default parameters, namely knowledgebase version 2, 0.1 percentage cutoff, and 1000 permutation times.

The 76 high-confidence tissue-specific secreted proteins were determined by counting all tissue-specific secreted proteins that overlapped a marker gene for the same cluster and were predicted to be secreted or transmembrane.

### Nuclei isolation from 3rd instar larvae, encapsulation, and sequencing for snRNA-seq

To synchronize developmental timing and prevent overcrowding of larvae, adult flies (*w1118*) were allowed to lay eggs on food supplemented with yeast pellets for 2–4 h. Progeny were raised at 25 °C. Five days later, 16 wandering third instar larvae (8 male, 8 female) that had not everted their spiracles were collected, washed with PBS, snap-frozen in liquid nitrogen, and stored at −80 °C.

Nuclei were isolated following a previously described protocol^55,116^. Briefly, 16 frozen larvae were homogenized in a glass dounce containing 1 mL of homogenization buffer (250 mM sucrose, 10 mM Tris, pH 8.0, 25 mM KCl, 5 mM MgCl_2_, 0.1% Triton-x 100, 0.5% RNaisin Plus [Promega, N2615], 1x protease inhibitor [Promega, G652A], 0.1 mM DTT). Nuclei were released by 20 strokes with a loose pestle, followed by 40 strokes with a tight pestle. Lysate containing nuclei was filtered sequentially through a 35 µm cell strainer (VWR, 21008-948) and a 40 µm cell strainer (Bel-Art, H13680-0040). A crude nuclei sample was isolated by centrifuging filtered lysate for 10 min at 1000×*g* at 4 °C, discarding the supernatant, and resuspending the pellet in 1000 µL 1×PBS/1%BSA by gentle pipetting. The crude nuclei sample was washed by three additional rounds of centrifugation and resuspension in 1×PBS/1%BSA. The washed nuclei sample was filtered through a 40 µm cell strainer (Bel-Art, H13680-0040), labeled with 1 µM DRAQ7 Dye (Invitrogen D15106), and 500,000 healthy nuclei were isolated by Sony SH800Z Cell Sorter into 1×PBS. FACS sorted nuclei were centrifuged for 10 min at 1000×*g* at 4 °C and resuspended in 250 µL 1×PBS/1%BSA + 1:1000 DAPI (manufacturer). Nuclei concentration was determined by counting DAPI+ nuclei on a hemocytometer, and sample concentration was adjusted to a final concentration of 1000 nuclei/µL in 1×PBS/1%BSA.

Nuclei were encapsulated and libraries created using the Chromium Next GEM Single Cell 3’ Reagents Kits v3.1, according to the 10X genomics protocol (Chromium Next GEM Single Cell 3’_v3.1_Rev_D). Samples were sequenced on an Illumina NovaSeq 6000 S4. (Following could be the data processing and analysis)

### Analysis of snRNA-seq data

Sequence alignment, cell calling, ambient RNA correction, and doublet removal were performed on eight replicates separately and before pooling for batch correction, dimension reductions, and clustering. Sequence alignment and cell calling were performed using CellRanger v7.1.0 (10X genomics) in ‘count’ mode using --force-cells=10000. DecontX (v1.0.0) was performed under default settings and used empty drops from each sample as background. After background correction, cells with fewer than 50 genes or 80 UMIs were removed. Cells with log10GenesPerUMI > 1 and UMI larger than 100,000 were also removed. DoubletFinder (v3) with 5% doublet formation rate (according to 10X suggestion) was used to remove potential multiplets from downstream analysis. After these quality control steps, about 30K high-quality cells from all samples were retained for downstream analysis. Harmony (v0.1) was performed on the retained cells, and the top 30 PC of the harmony-corrected PCs were used for UMAP visualization and Leiden clustering. Cells were grouped and annotated under clustering resolution = 1 for the best separation of rarer cell types by top marker genes. The web-based portal for data mining was implemented (https://www.flyrnai.org/scRNA/body_larvae/).

### RNA-seq expression analysis of GFP-tagged genes

RNA-seq expression values were obtained from DGET^115^. Genes were classified based on the presence or absence of a detectable GFP-tagged protein band by Western blot. Expression levels were compared between band-positive and band-negative genes using two-sided Mann–Whitney *U* tests, performed independently across six larval developmental stages. Average ranks were used to account for tied expression values.

### Reporting summary

Further information on research design is available in the Nature Portfolio Reporting Summary linked to this article.

## Supplementary information

Supplementary Information

Supplementary Data 1

Supplementary Data 2

Supplementary Data 3

Supplementary Data 4

Supplementary Data 5

Supplementary Data 6

Supplementary Data 7

Supplementary Data 8

Reporting Summary

Transparent Peer Review file

## Source data

Source Data

## Data Availability

Plasmids are available from Addgene and the *Drosophila* Genomic Resource Center (DGRC). Cell lines are available from the DGRC. Fly lines are available from the Bloomington *Drosophila* Stock Center (BDSC). Raw mass spectrometry data for the pilot secretome experiments from S2R+ and 3rd instar larvae are available from FigShare. 10.6084/m9.figshare.31286095. Raw mass spectrometry data for the Tissue Secretome Map (TMT experiment) and Blood proteome (Label-free experiment) have been deposited in the public proteomics repository MassIVE and are accessible at accession number MSV000098658. ftp://massive-ftp.ucsd.edu/v10/MSV000098658/. Raw snRNA-seq is available from the GEO at accession number GSE302679. https://www.ncbi.nlm.nih.gov/geo/query/acc.cgi?acc=GSE302679. snRNA-seq data can also be accessed on a web-based portal: https://www.flyrnai.org/scRNA/body_larvae/. Raw graph data, western blots, and gels are in Source Data. Source data are provided with this paper.
