# Supplementary material for: Multi-omic mapping of Drosophila protein secretomes reveals tissue-specific origins and inter-organ trafficking
Source: Nat Commun. 2026 Apr 20;17:5425. doi: 10.1038/s41467-026-71763-8 (PMC13280006; doi:10.1038/s41467-026-71763-8)
Supplement: Supplementary file 1 — Supplementary Information [file 41467_2026_71763_MOESM1_ESM.pdf]

Supplementary Information

Multi-omic mapping of Drosophila protein secretomes reveals tissue-specific origins and inter-organ trafficking

Bosch et al. 2026

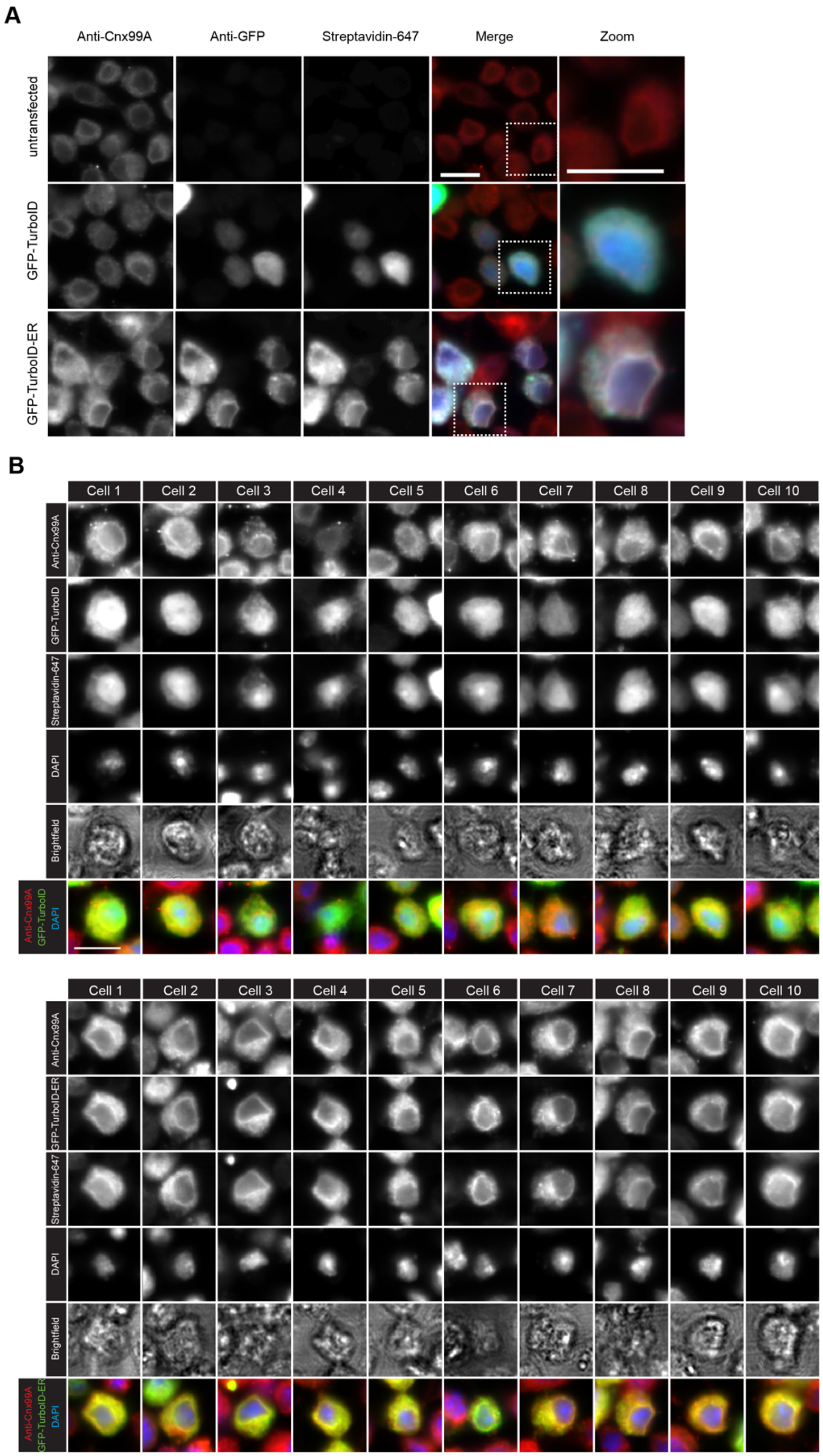

Supplementary Figure 1: Imaging-based localization of GFP-TurboID proteins and biotin labeling in transfected S2R+ cells. (A) Confocal microscopy of S2R+ cells transfected with *pMT-GFP-TurboID* or *pMT-GFP-TurboID-ER*. ER detected with Anti-Cnx99A (red), biotinylated proteins detected with streptavidin-647 (blue), and GFP-TurboID detected with anti-GFP (green). Zoomed regions indicated by white boxes. Scale bar is 10μm. Red and green channel signal intensity is the same for all samples. Blue channel signal for GFP-TurboID sample was lowered to match the signal intensity of GFP-TurboID-ER sample. **(B)** Representative images of 10 individual transfected cells expressing either GFP-TurboID (top) or GFP-TurboID-ER (bottom) to illustrate ER-localization of GFP-TurboID-ER and streptavidin-647.

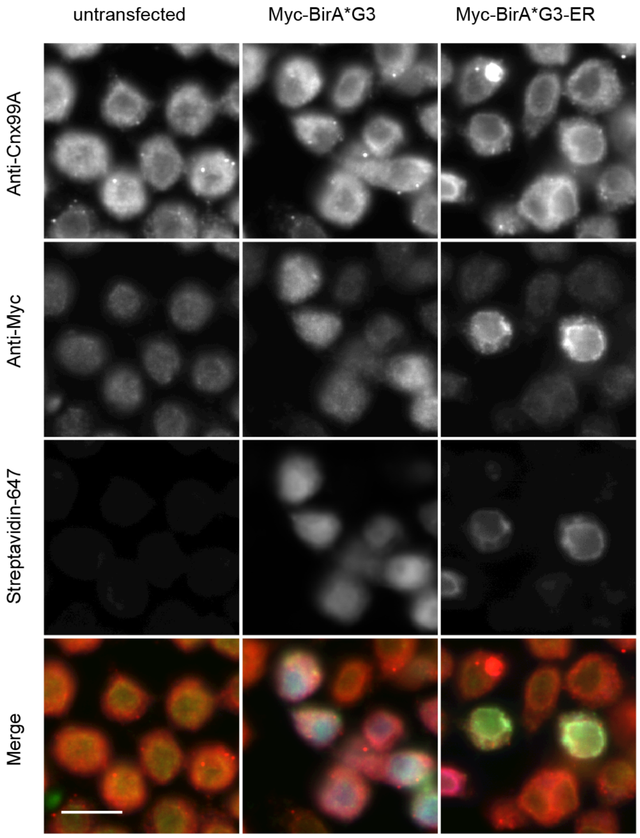

Supplementary Figure 2: Imaging-based localization of Myc-BirA*G3 and Myc-BirA*G3-ER proteins and biotin labeling in transfected S2R+ cells. Confocal microscopy of S2R+ cells transfected with *pMT-Myc-BirA*G3* or *pMT-Myc-BirA*G3-ER*. ER detected with Anti-Cnx99A (red), biotinylated proteins detected with streptavidin-647 (blue), and GFP-TurboID detected with anti-GFP (green). Scale bar is 10µm. Red and green channel signal intensity is the same for all samples. Blue channel signal for Myc-BirA*G3 sample was lowered to match the signal intensity of Myc-BirA*G3-ER sample. Experiments were repeated twice with similar results.

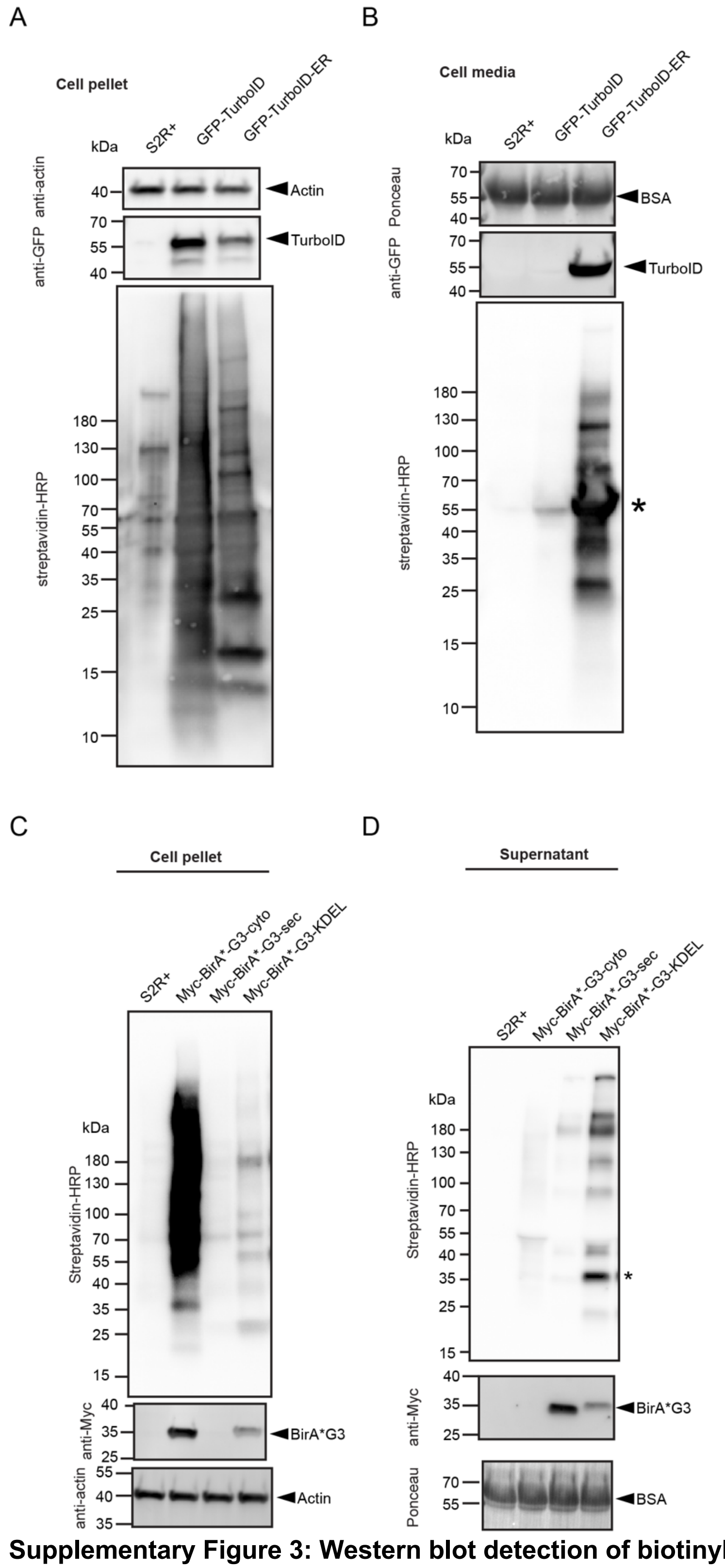

Supplementary Figure 3: Western blot detection of biotinylated proteins in S2R+ cell pellets and media transfected with TurboID and BirA*G3 constructs. (A-B) Western blot of cell lysates **(A)** or cell media **(B)** from S2R+ cells stably expressing GFP-TurboID or GFP-TurboID-ER. Each lane loaded 15μg protein. Arrowheads indicate expected band, asterisk indicates auto-biotinylated GFP-TurboID. Experiments were repeated twice with similar results. **(C-D)** Western blot of cell lysates **(C)** or cell media **(D)** S2R+ cells transfected with *pMT-Myc-BirA*G3* or *pMT-Myc-BirA*G3-ER*. Each lane loaded 15μg protein. Arrowheads indicate expected band, asterisk indicates auto-biotinylated Myc-BirA*G3. Ponceau staining shown is from a parallel replicate blot loaded with identical samples and protein amounts. Experiments were repeated twice with similar results.

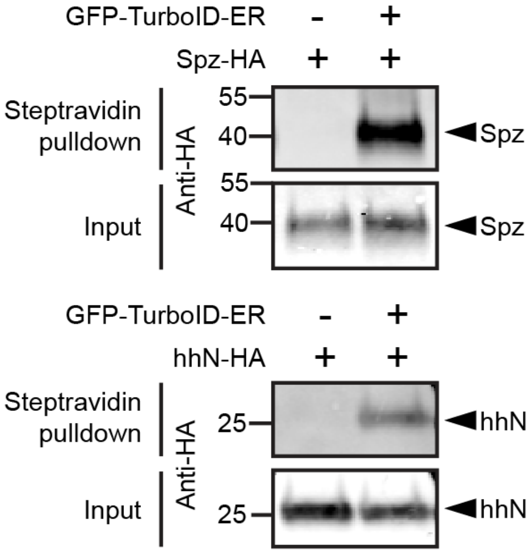

Supplementary Figure 4: Streptavidin-pulldown of biotinylated HA-tagged known secreted proteins. Western blot of streptavidin-pulldown of HA-tagged secreted proteins in media from S2R+ cells transfected with *pMT-GFP-TurboID-ER*. Arrowheads indicate expected bands. Each lane loaded 15μg protein. Predicted molecular weight: Spz-HA 38.3 kDa, hhN-HA 23.6 kDa.

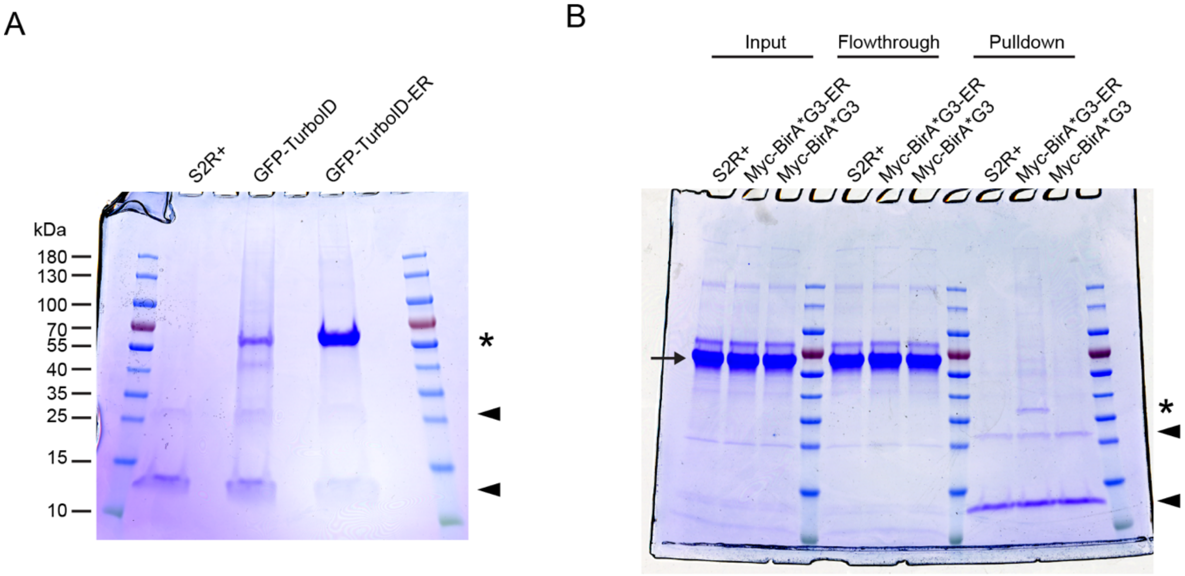

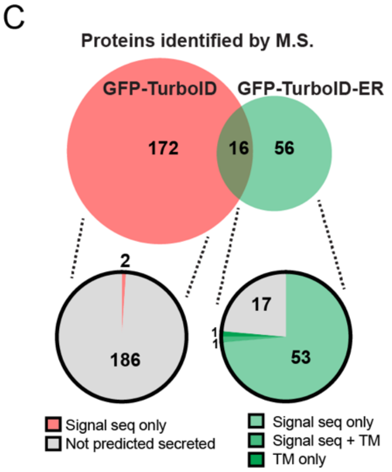

D

Protein	Spectral counts	Function
Grp170	41	Chaperone
LanA	28	Extracellular Matrix
CG5080	24	Unknown
Irc	22	Anti-microbial
LanB1	10	Extracellular Matrix
LanB2	9	Extracellular Matrix
CG7484	5	Unknown
Pvf2	2	FGF ligand
Daw	2	Activin-beta ligand
CG12093	2	Unknown

Supplementary Figure 5: Large-scale pulldown of biotinylated proteins from the media of GFP-TurboID-ER expressing S2R+ cells (A) Coomassie-stained SDS-PAGE gel of protein streptavidin-pulldown from 30ml media from *pMT-GFP-TurboID* or *pMT-GFP-TurboID-ER* stable cell lines. Arrowheads indicate streptavidin monomer and dimer. Asterisk indicates auto-biotinylated GFP-TurboID. Note this used the large-scale pulldown protocol (see methods). **(B)** Coomassie-stained SDS-PAGE gel of protein streptavidin-pulldown from 1ml media from *pMT-GFP-TurboID* or *pMT-GFP-TurboID-ER* stable cell lines. Gel includes input and post-bead flowthrough. Arrowheads indicate streptavidin monomer and dimer. Asterisk indicates auto-biotinylated Myc-BirA*G3. Arrow indicates bovine serum albumin (BSA) from the fetal bovine serum (FBS) in cell culture media. Note this used the small-scale pulldown (see methods). **(C)** Analysis of proteins identified following whole biotinylated protein pulldown from cell media, gel lane excision, and liquid chromatography-tandem mass spectrometry (LC-MS/MS). Venn diagram showing the number of proteins identified (top), pie charts showing the fraction of predicted secreted proteins (bottom). **(D)** Table showing example secreted proteins identified from *pMT-GFP-TurboID-ER* cell media.

A

Recombinant TurboID fly strain	Tissue-specific labeling
UAS-GFP-TurboID-ER	None (control)
lpp-Gal4, UAS-GFP-TurboID-ER	Fat body
mef2-Gal4, UAS-GFP-TurboID-ER	Muscle
myo1a-Gal4, UAS-GFP-TurboID-ER	Gut enterocytes
elav-Gal4, UAS-GFP-TurboID-ER	Neurons
repo-Gal4, UAS-GFP-TurboID-ER	Glia
btl-Gal4, UAS-GFP-TurboID-ER	Trachea
prome-Gal4, UAS-GFP-TurboID-ER	Oenocytes
ms1096-Gal4, UAS-GFP-TurboID-ER	Wing disc
hml-Gal4, UAS-GFP-TurboID-ER	Hemocytes
hand4x-Gal4, UAS-GFP-TurboID-ER	Heart/Nephrocytes

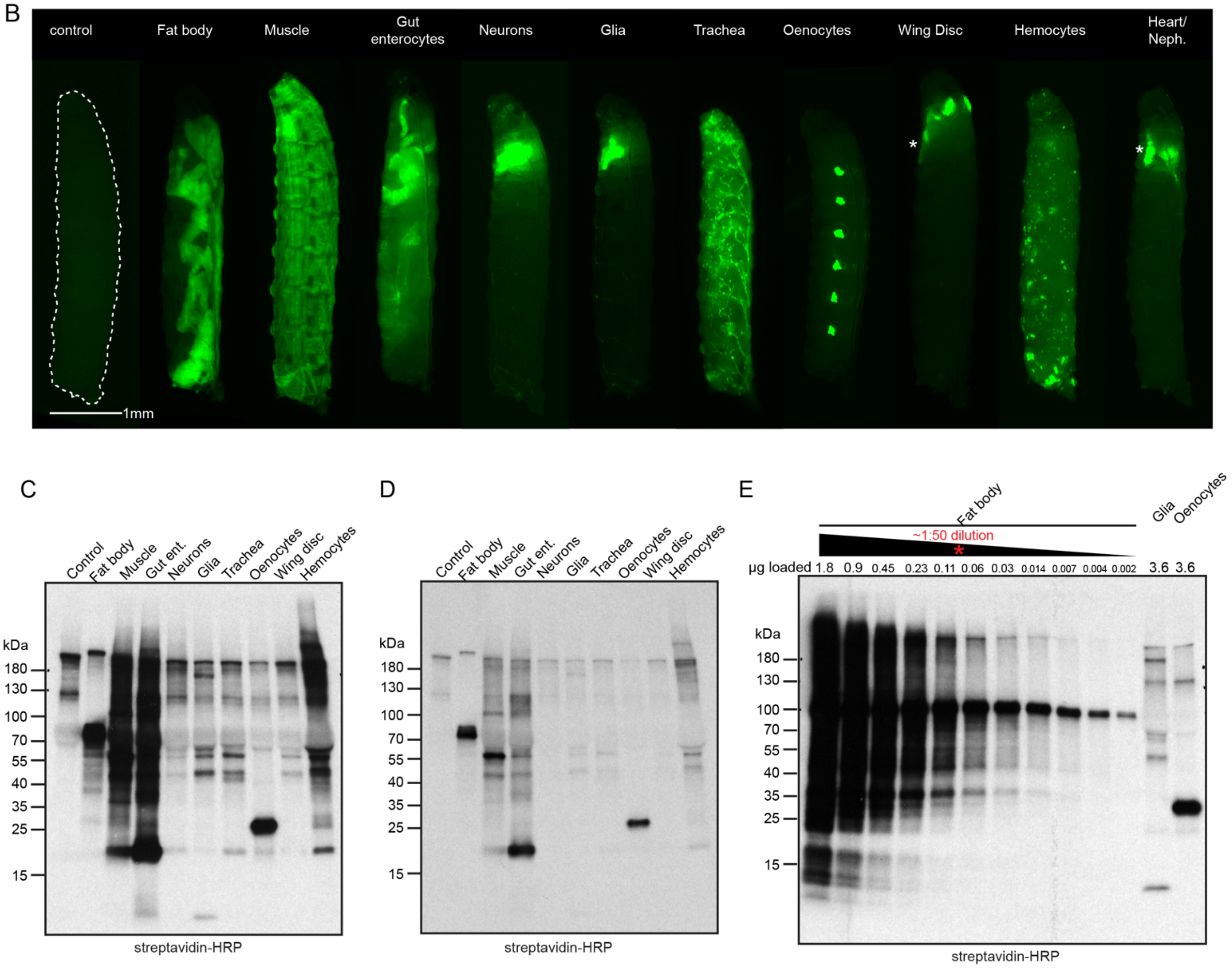

Supplementary Figure 6: In vivo labeling of secreted proteins in specific tissues of *Drosophila* 3rd instar larvae. (A) Table of recombinant fly strains expressing GFP-TurboID-ER in major tissue types. **(B)** Stereo fluorescence microscopy 3rd instar larvae. Recombinant fly strain expression visualized with *UAS-6x-GFP* crossed to recombinant lines. Asterisks indicate expression in salivary glands. **(C)** Western blot of biotinylated proteins in purified hemolymph from 20 3rd instar larvae expressing GFP-TurboID-ER in specific tissues. All lanes loaded 3.6µg protein, except the fat body labeling sample (72ng). Experiments were repeated twice with similar results. **(D)** Lower exposure of blot in **(C)**. **(E)** Western blot of biotinylated proteins in purified hemolymph from 3rd instar larvae expressing GFP-TurboID-ER in specific tissues. Serial dilution of fat body labeling sample to identify 72ng as comparable signal intensity to glia and oenocyte labeling samples.

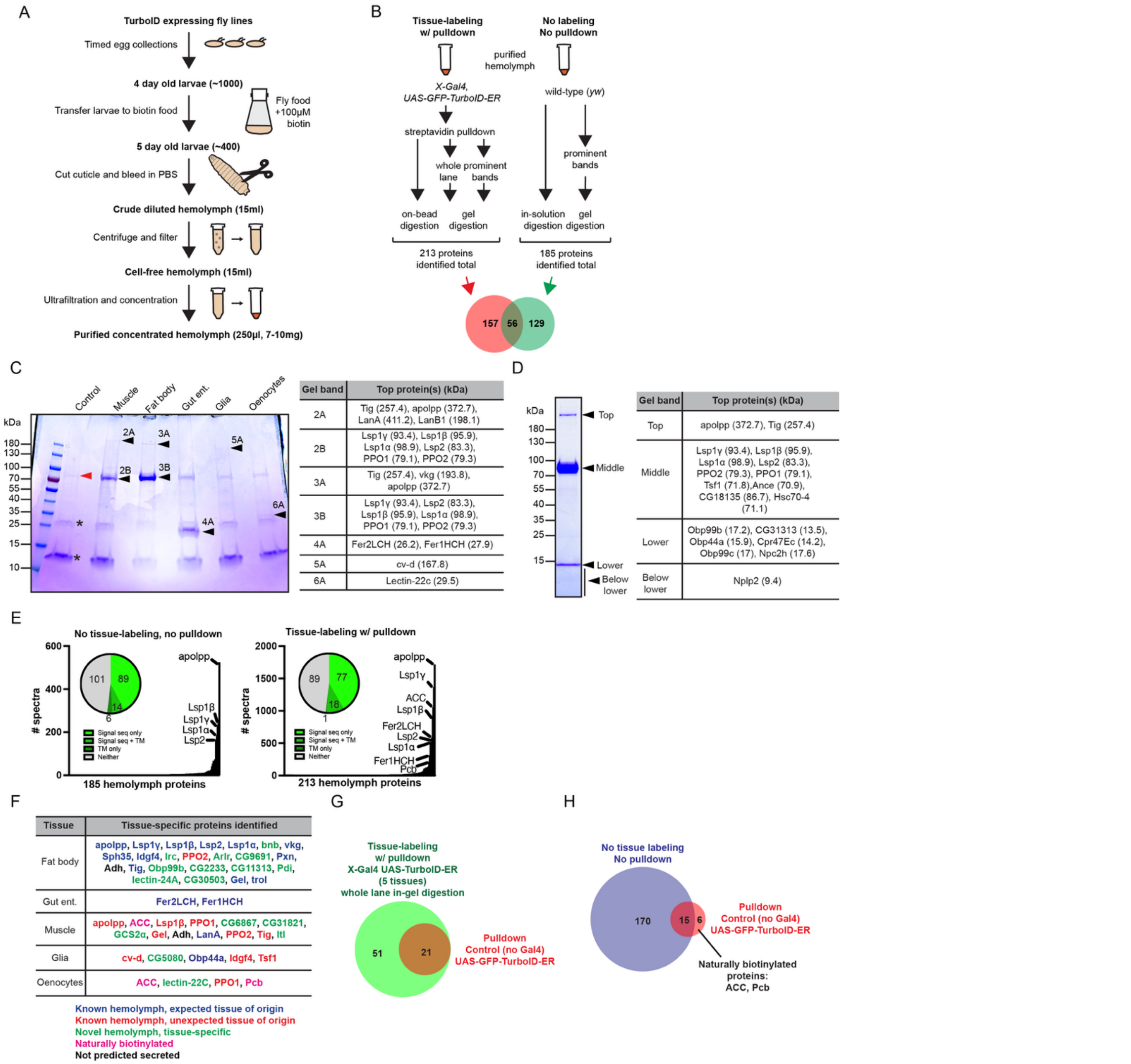

Supplementary Figure 7: Large-scale pulldown hemolymph biotinylated proteins from 3rd instar hemolymph expressing tissue-specific GFP-TurboID-ER. (A) Schematic of large-scale hemolymph isolation and purification from 3rd instar larvae. **(B)** Schematic of five experiments identifying proteins from hemolymph by LC-MS/MS. Three experiments were performed using tissue-labeling with streptavidin pulldown, and two using no labeling and no streptavidin pulldown. Venn diagram shows the number of proteins identified and overlap. **(C)** Coomassie-stained SDS-PAGE gel of protein streptavidin-pulldown from purified hemolymph from 3rd instar larvae expressing GFP-TurboID-ER in specific tissues (left). Black arrowheads indicate protein bands excised for mass spectrometry. Red arrowhead indicates likely non-specific pulldown of LSPs. Asterisks indicate streptavidin monomer and dimer. Table (right) shows top proteins identified. **(D)** Coomassie-stained SDS-PAGE gel of purified hemolymph from 3rd instar larvae (left). Black arrowheads indicate protein bands excised for mass spectrometry. Table (right) shows top proteins identified. **(E)** Analysis of proteins identified following mass spectrometry, ranked by # of peptide spectra, and predicted as secreted. **(F)** Table of 48 tissue-enriched proteins identified from tissue-labeling streptavidin pulldown and whole-lane gel excision. Enriched proteins defined as ≥0.25 spectral counts/sum spectral counts all experimental samples, and at least 3 spectral counts in enriched tissue. Previous characterization determined using Flybase ("hemolymph" GO term) and literature searching. **(G)** Venn diagram of # of proteins identified by LC-MS/MS in tissue-labeling vs. no labeling control for pulldown experiments involving whole lane in-gel digestion. **(H)** Venn diagram of # of proteins identified by LC-MS/MS in raw hemolymph (no pulldown) vs. no labeling control with pulldown involving whole lane in-gel digestion

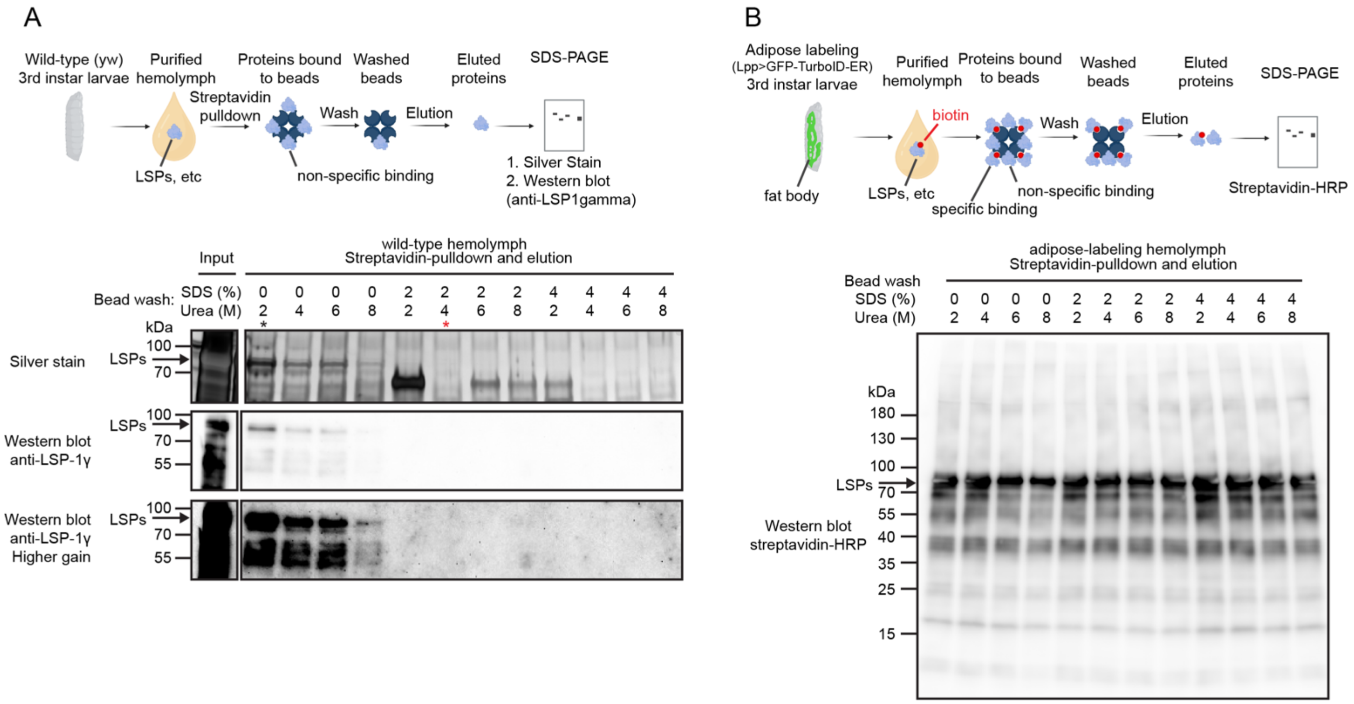

Supplementary Figure 8: Improved streptavidin-bead washing. (A) Experiment schematic and results. Silver stained SDS-PAGE gel (top) and western blot (middle/bottom) of eluted proteins after streptavidin pulldown of purified hemolymph from wild-type 3rd instar larvae. Arrow indicates molecular weight of LSPs. Different beads washing conditions are indicated. Improved washing condition uses 2% SDS and 4M Urea (red asterisk) compared to previous washing condition of 2M Urea (black asterisk). Created in BioRender. Bosch, J. (2026) https://BioRender.com/b8yidst **(B)** Experiment schematic and results. Western blot of eluted biotinylated proteins after streptavidin pulldown of purified hemolymph from 3rd instar larvae with fat body labeling. Arrow indicates molecular weight of LSPs. Different beads washing conditions are indicated.

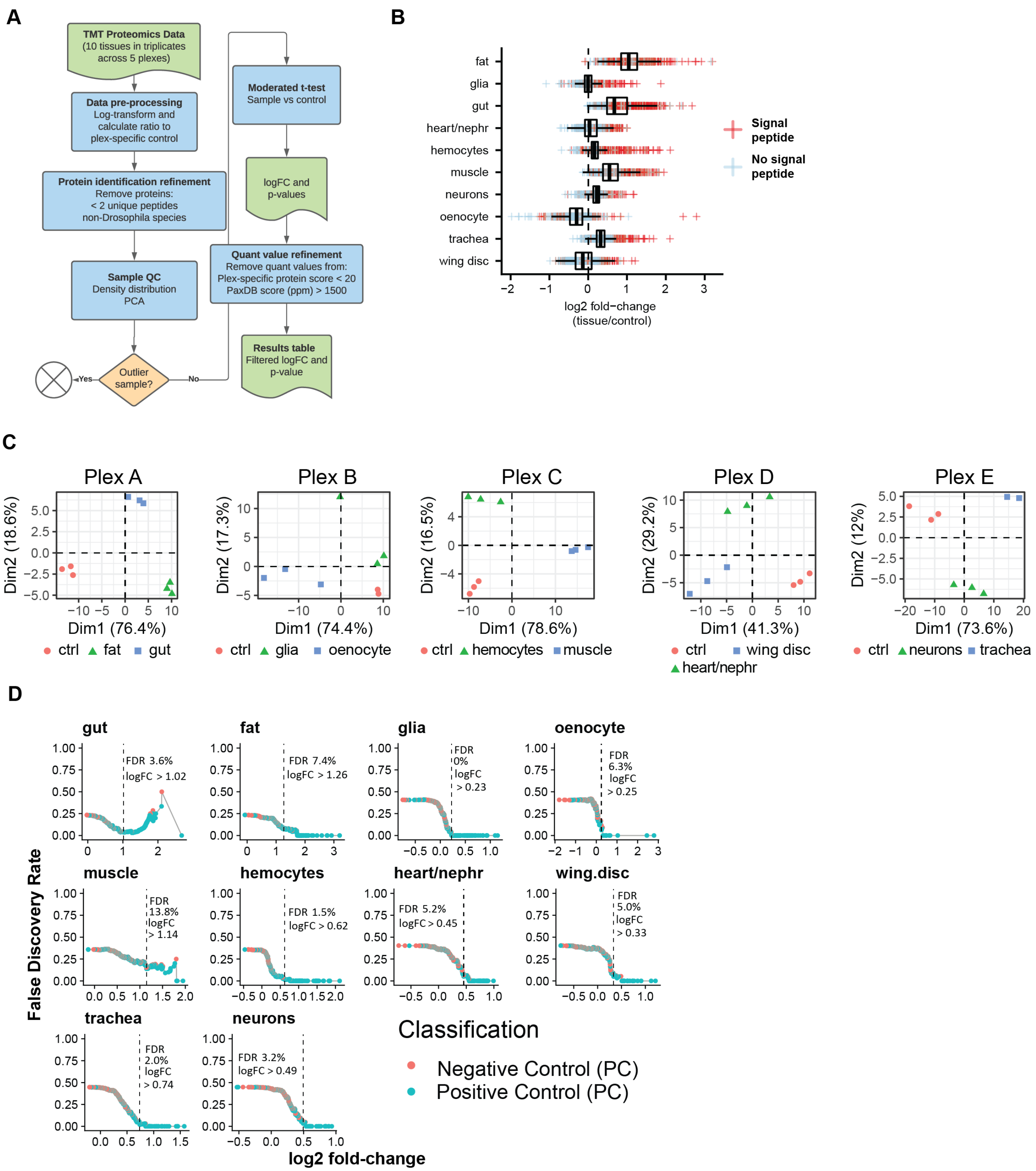

Supplementary Figure 9: Workflows and data related to quantitative TMT proteomics experiments. (A) Workflow for data processing and filtering criteria of proteomics data from tissue secreted proteins. **(B)** Boxplots showing log2 fold-change of TMT intensities for each of the 10 tissues relative to the appropriate control. Individual protein values are shown in the plot with color indicating the content of a signal peptide. Enrichment of signal peptide-containing proteins is observed at higher fold-changes for all tissues. Signal peptide annotation by Uniprot. **(C)** Principal component analysis plots for each multiplexed set of samples show cluster of biological replicate samples. **(D)** Scatter plots showing empirical false discovery rate as a function of log2 fold-change cutoffs using negative and positive controls for classification. The dotted line indicates manually selected point that tries to minimize the fold-change threshold at an acceptable empirical false discovery rate.

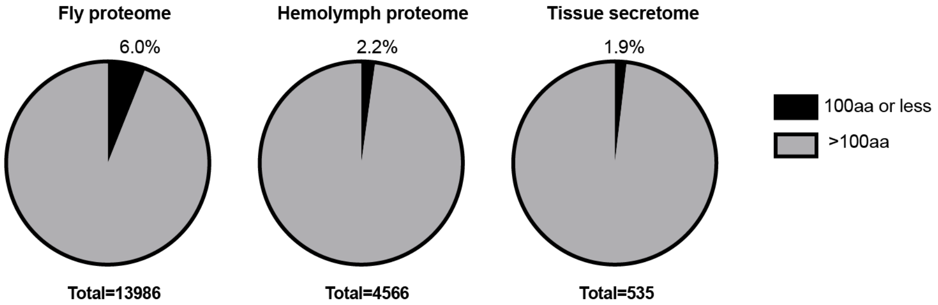

Supplementary Figure 10: Small proteins in tissue-secretome and hemolymph proteomic datasets. Pie charts showing the proportion of small proteins (100aa or less) in the fly proteome, hemolymph proteome, and tissue secretome datasets.

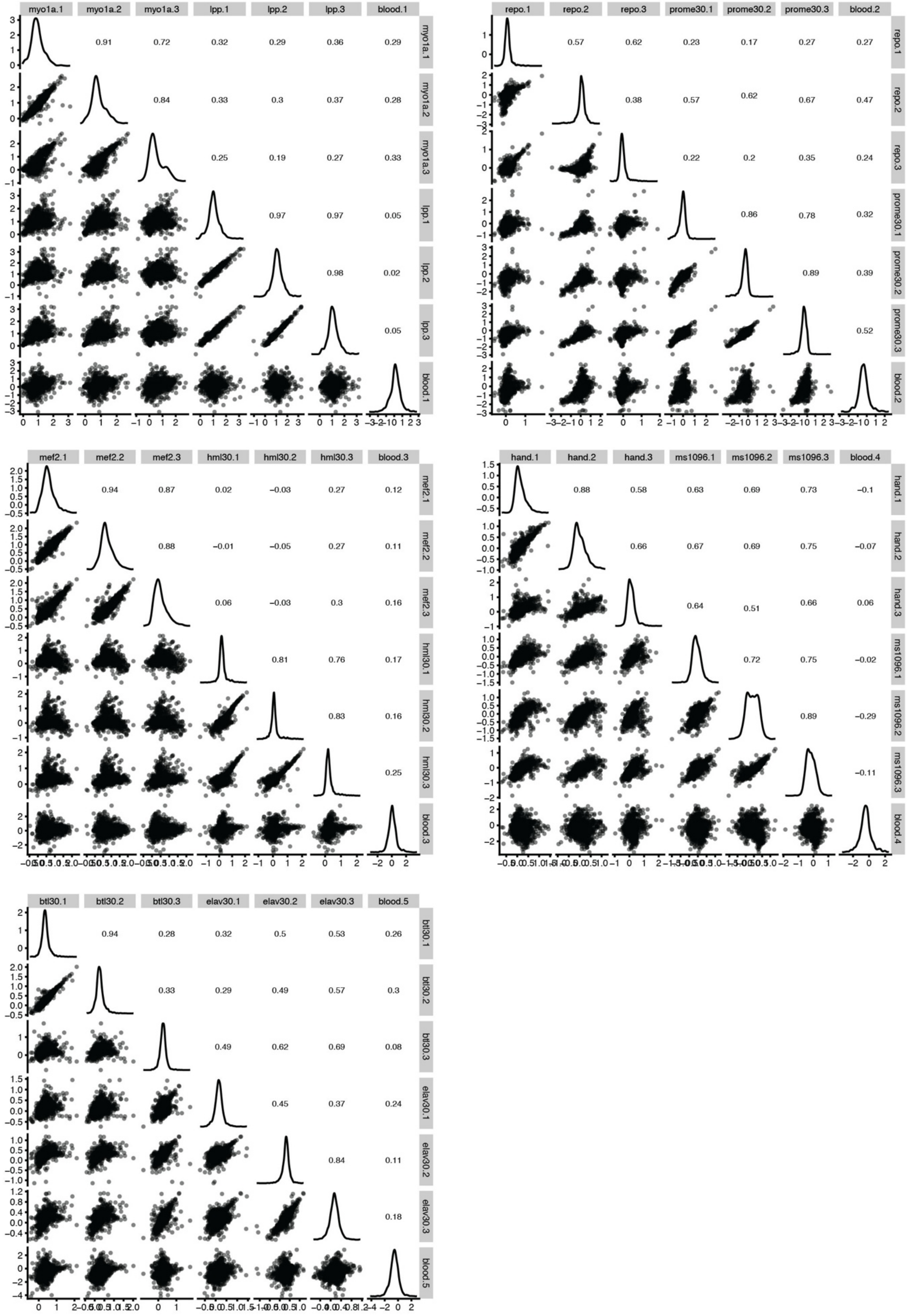

Supplementary Figure 11: Pairwise comparison of protein enrichment across tissue secretome samples. Pairwise plot matrix between samples in the tissue secretome dataset. Pairwise TMT ratio data was plotted within each TMT plex experiment. Scatter plots are shown below the diagonal, density distributions on the diagonal, and Pearson correlation values are shown above the diagonal.

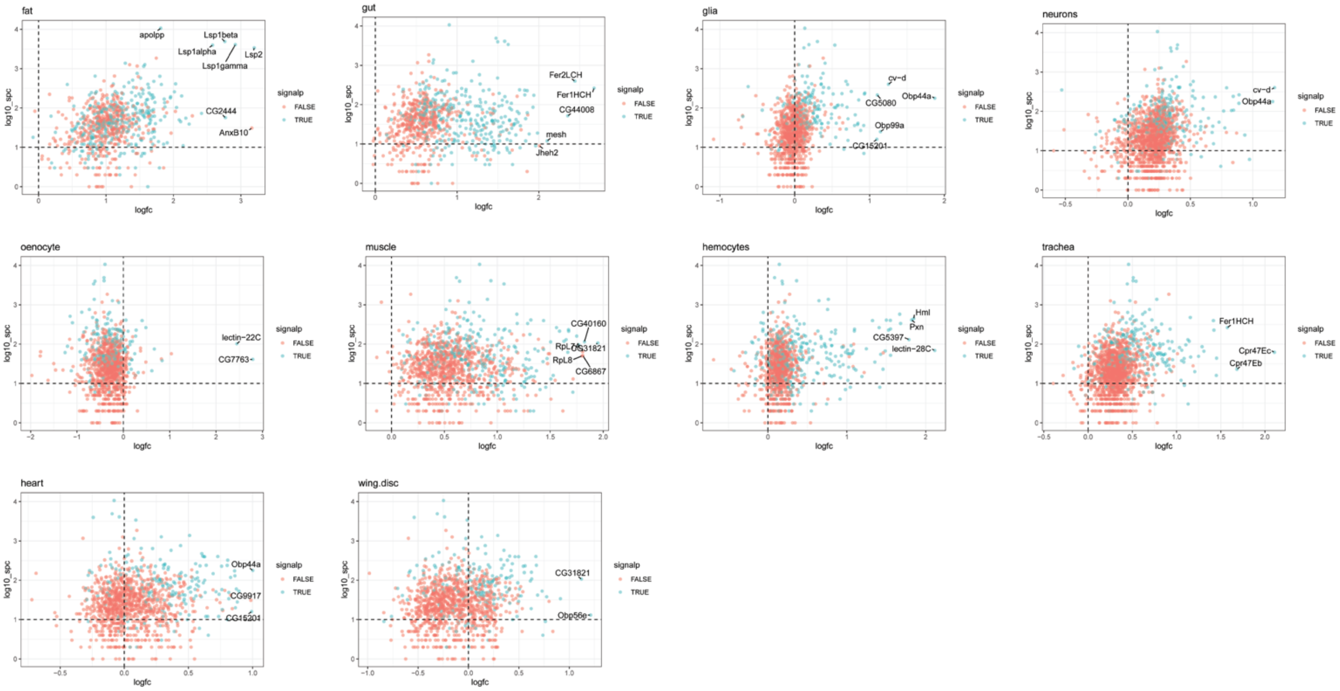

Supplementary Figure 12: Comparison of tissue-specific enrichment versus bulk hemolymph abundance of secreted proteins. Scatter plot showing log_2 fold-change from the tissue secretome dataset (tissue/control; logfc) vs the label-free protein abundance from the whole blood proteome dataset as log_{10} spectral counts (log10_spc). Color indicates the content of a signal peptide. Signal peptide annotation by Uniprot. Proteins with a large logfc are highlighted.

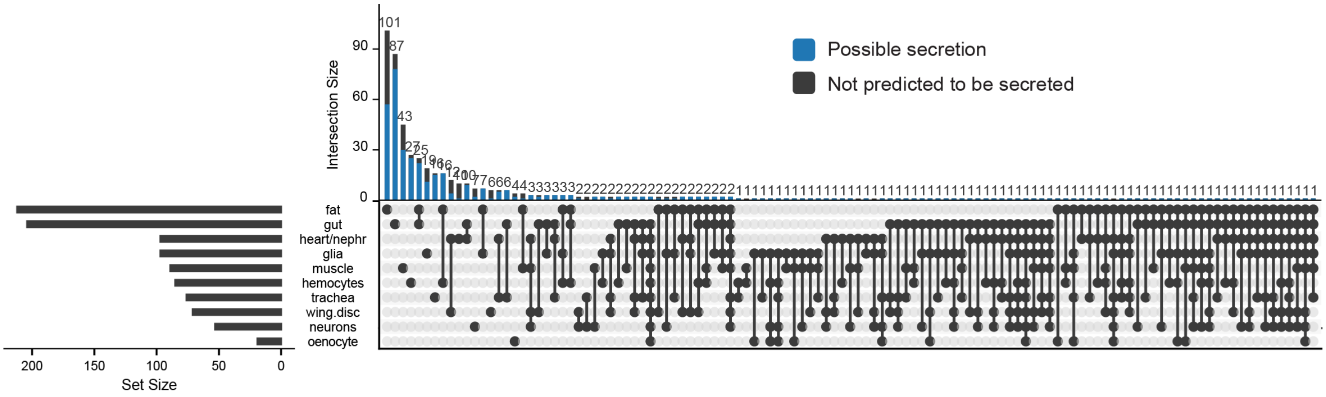

Supplementary Figure 13: Tissue-specificity map of 535 identified proteins. Upset plot showing the number of proteins identified as secreted by a unique tissue or by multiple tissues. The blue portion of the bar indicates the proportion of proteins annotated as secreted of transmembrane for each intersect.

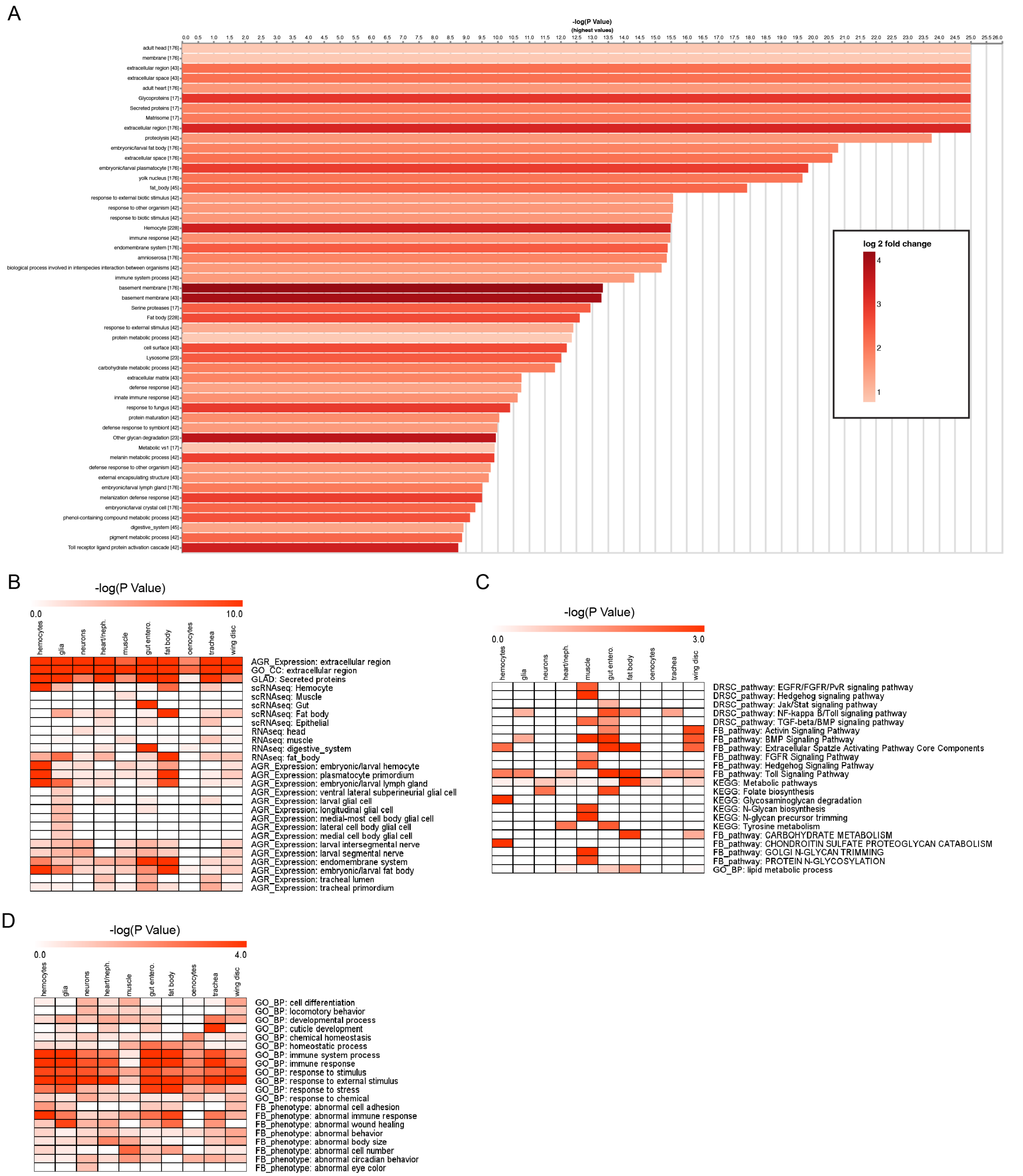

Supplementary Figure 14: Gene set enrichment analysis of tissue-secreted proteins using PANGEA. (A) Bar graph of gene set enrichment analysis on 535 tissue-secreted proteins using PANGEA. Y-axis shows gene set name and [category ID], X-axis shows -log(P value) with bar color intensity showing log 2 fold change. **(B-D)** Enrichment heatmap of gene set enrichment analysis on secreted proteins from 10 individual tissues. **(B)** Subcellular localization and tissue specificity based on gene set annotation from bulk and scRNAseq datasets as well as AGR/GO/DRSC annotation. **(C)** Signaling and metabolic pathways analysis based on FlyBase, DRSC and KEGG annotation. **(D)** Biological process and phenotype analysis based on gene ontology and FlyBase annotation.

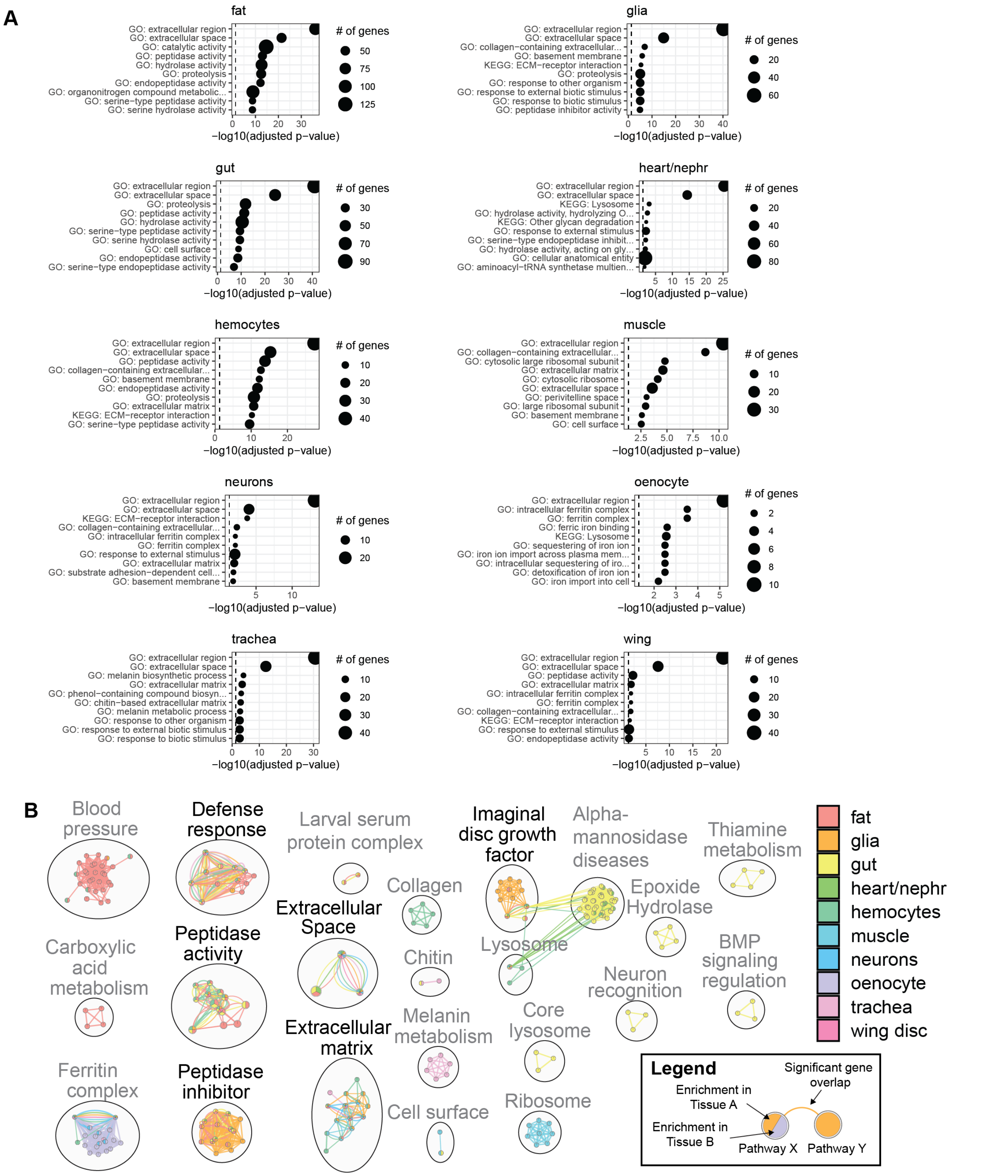

Supplementary Figure 15: Gene set enrichment analysis of tissue-secreted proteins using g:Profiler. (A) Dot plot showing the top 10 pathways from pathway enrichment results for each tissue using g:Profiler. Size indicates the number of genes in the pathway. **(B)** Network summary of pathway enrichment results. Nodes indicate pathways with FDR-adjusted p-value below 0.05 colored by the tissue in which the enrichment was observed. Edges indicate that two pathways share a large proportion of genes. Clusters of nodes indicate pathways with similar gene sets with a manual annotation of the common biological theme. Clusters of particular interest are highlighted: immune response, peptidase activity, peptidase inhibitor, extracellular space, extracellular matrix, and imaginal disc growth factor.

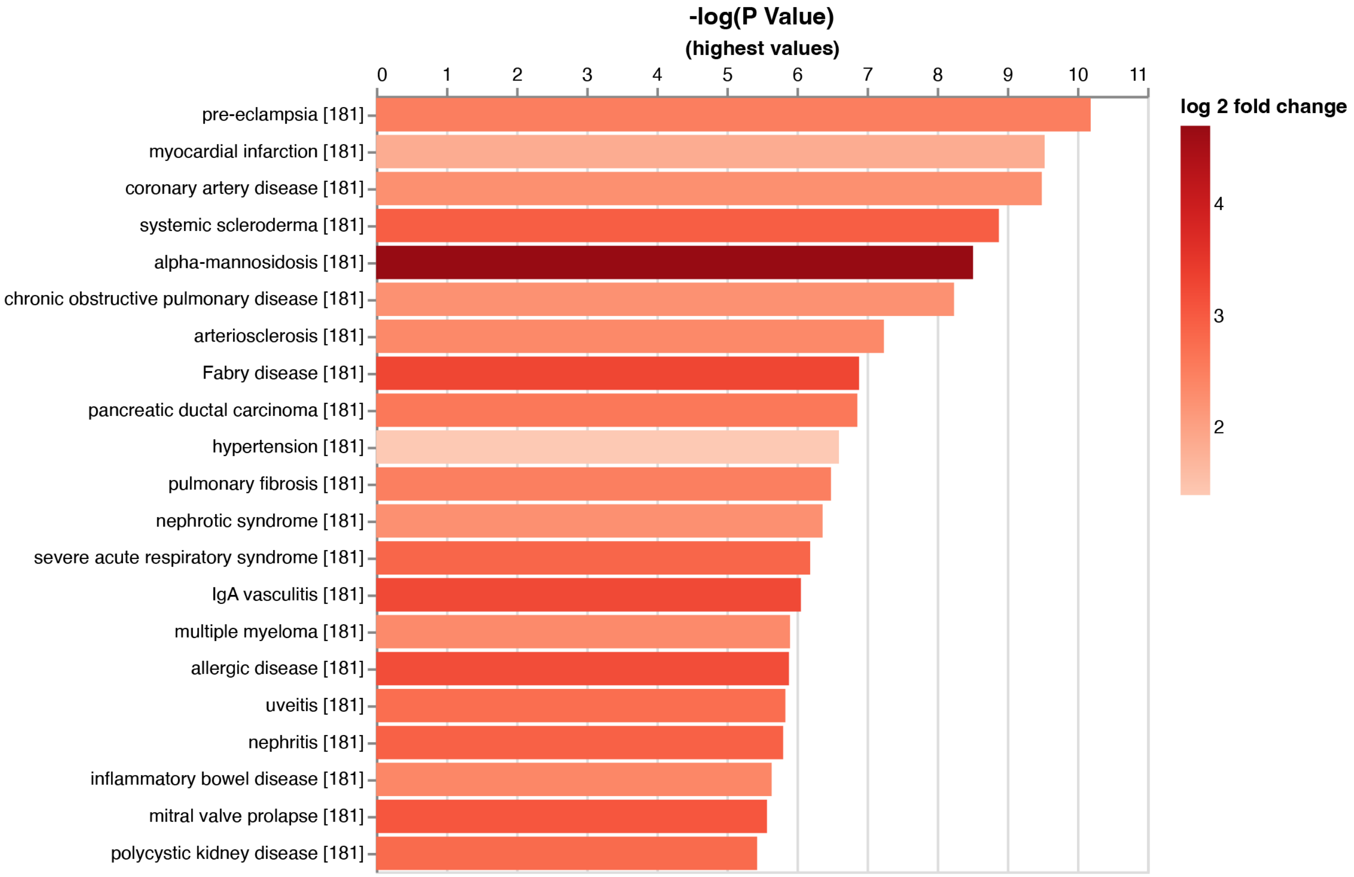

Supplementary Figure 16: Gene set disease enrichment analysis of tissue-secreted proteins. Bar graph of gene set enrichment analysis on 535 tissue-secreted proteins using PANGEA using Disease annotation AGR.

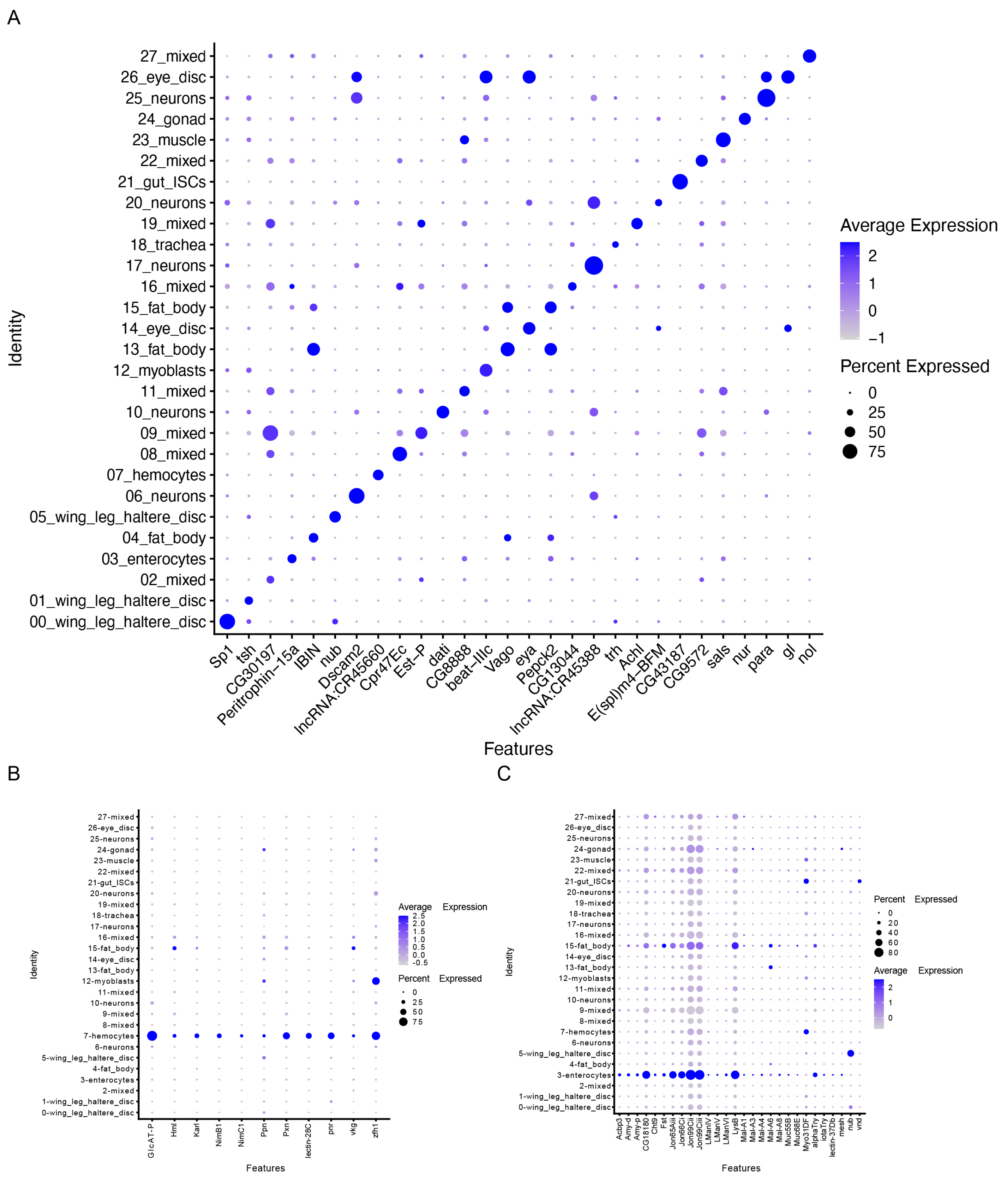

Supplementary Figure 17: Marker gene expression analysis in the larval snRNA-seq atlas. (A-C) Dot plots showing expression levels and percentage of cells expressing the top marker gene in each cluster based on average expression (logFC). Color gradient of the dot represents the expression level, while the size represents percentage of cells expressing the marker gene per cluster. **(A)** Top marker gene of all clusters. **(B)** Hemocyte marker genes. **(C)** Gut enterocyte marker genes.

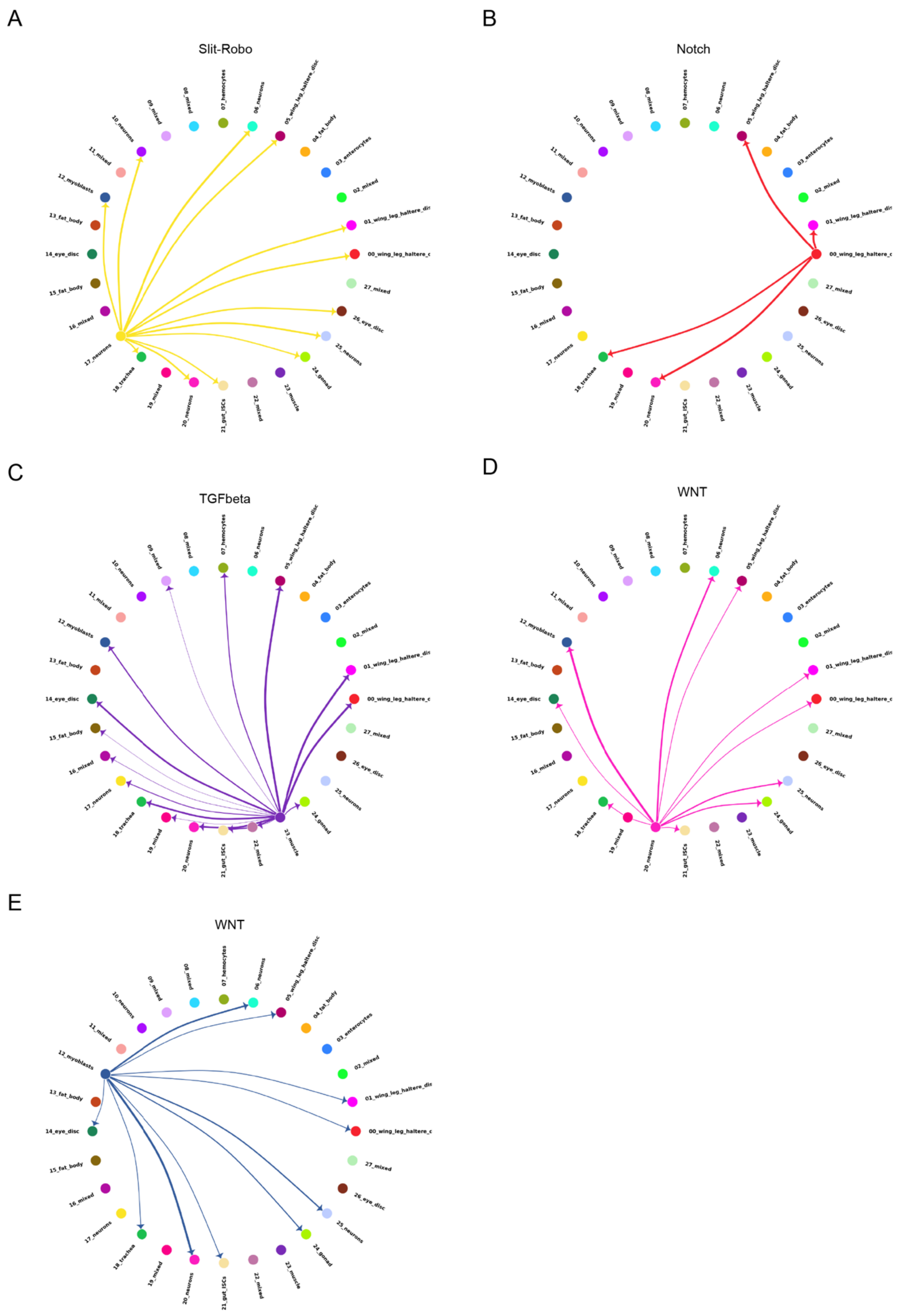

Supplementary Figure 18: Circle plots of signaling pathway networks inferred by FlyPhoneDB2 (A-E)
Circle plots with indicated signaling pathway, cell clusters from snRNA-seq map, and arrows showing directionality of cluster-to-cluster ligand->receptor relationships.

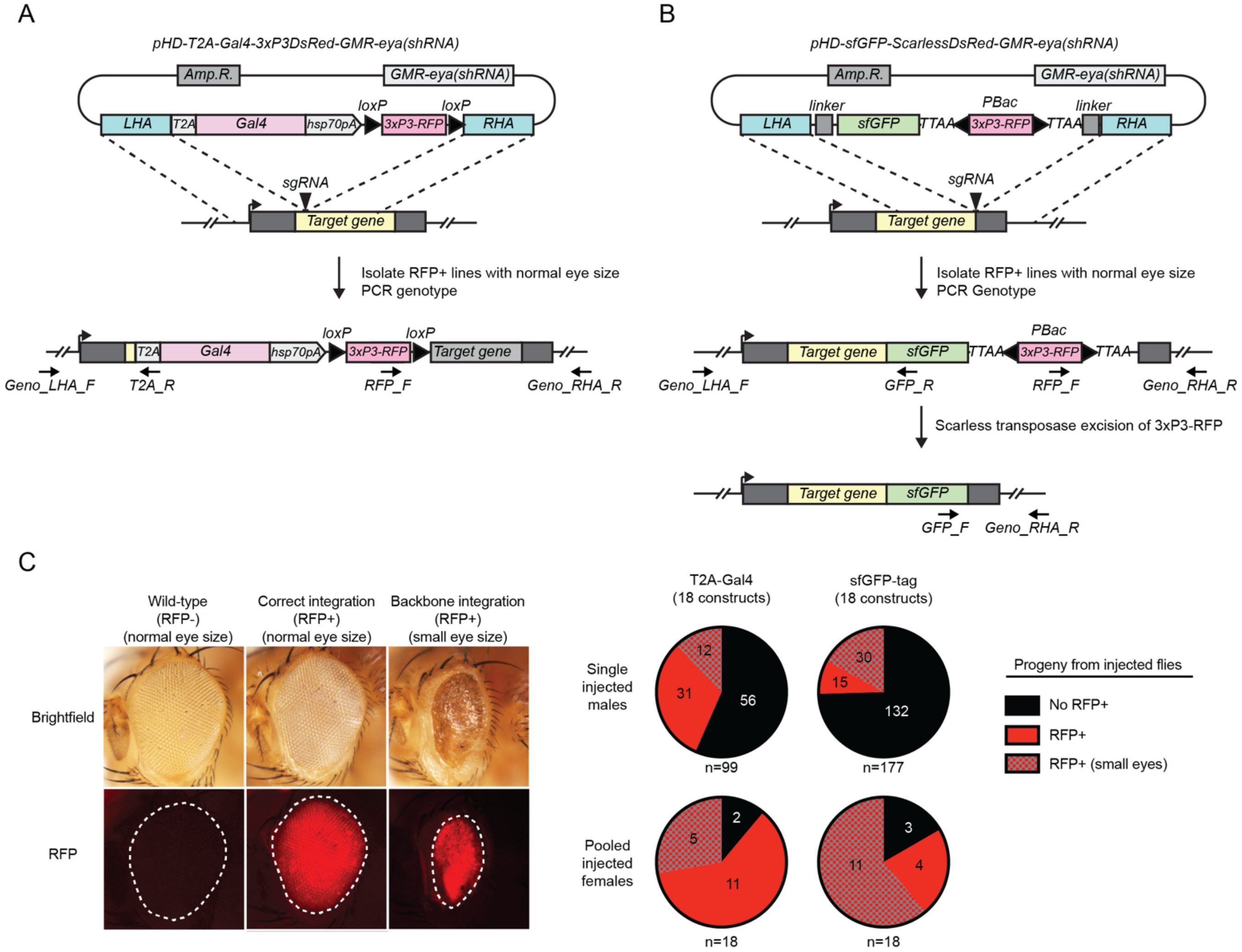

Supplementary Figure 19: CRISPR-HDR knock-in strategy, dual-selection screening, and integration outcomes (A-B) Schematic of donor plasmid, target gene, location of homology arms, sgRNA cut site, resulting knock-in locus, and location of genotyping primers. **(A)** T2A-Gal4 knock-in strategy. **(B)** GFP knock-in strategy. **(C)** Stereo fluorescence microscopy of adult fly eyes (left) and knock-in statistics (right). N values indicate the number of single injected males or pooled injected females yielding progeny. Percentage of single males or female pools giving rise to any RFP+ progeny is shown in red and red/grey checkered pattern. Percentage of single males or female pools giving rise to any RFP+/small eye progeny is shown in a red/grey checkered pattern.

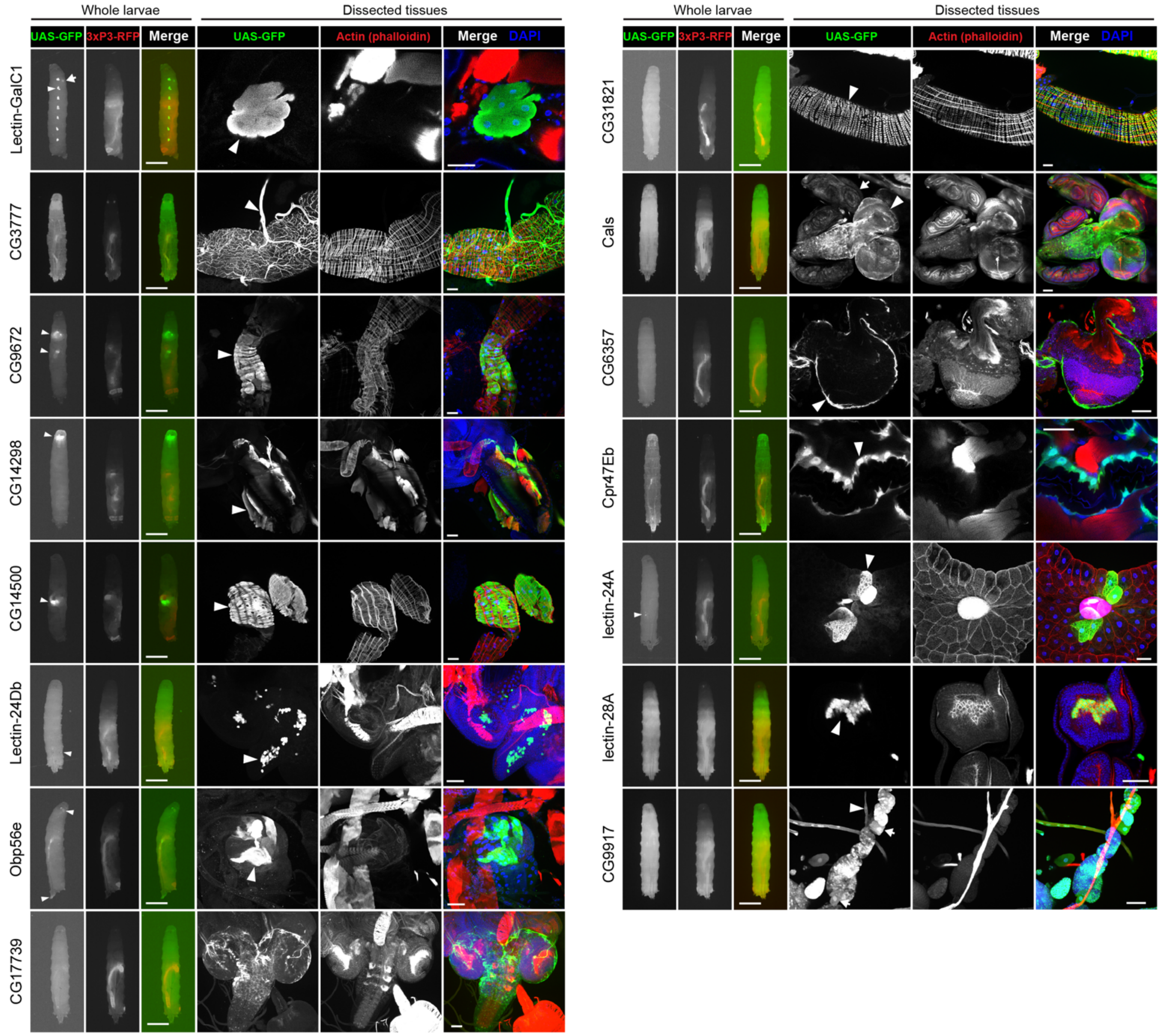

Supplementary Figure 20: Additional gene expression patterns of T2A-Gal4 knock-in lines. Widefield fluorescence microscopy images of whole 3rd instar larvae, or confocal microscopy of dissected tissues from 3rd instar larvae, expressing *UAS-GFP* (green) under the control of a *gene-T2A-Gal4* transgene. GFP fluorescence was imaged in whole heat-killed larvae or using anti-GFP-488 (green) antibodies in dissected tissues. Also shown is *3xP3-RFP* (red) marker fluorescence for whole larvae, and phalloidin (red) and DAPI (blue) for confocal images of dissected tissues. For confocal images, the imaged tissue(s) are: Lectin-GalC1 (arrowhead indicates oenocytes), CG3777 (arrowhead indicates trachea associated with gut), CG9672 (arrowhead indicates gut enterocytes), CG14298 (arrowhead indicates mouth hooks), CG14500 (arrowhead indicates gut enterocytes), Lectin-24Db (arrowhead indicates hemocytes associated with eye disc), Obp56e (arrowhead indicates spiracles), CG17739 (brain), CG31821 (arrowhead indicates visceral muscle), Cals (arrowhead indicates brain and arrow indicates imaginal discs), CG6357 (arrowhead indicates perineural glia), Cpr47Eb (arrowhead indicates epidermis), lectin-24A (arrowhead indicates fat body cells adjacent to gonad), lectin-28A (arrowhead indicates hemocytes associated with eye disc), CG9917 (arrowhead indicates heart tube, arrows indicate pericardial nephrocytes). Scale bars are 1mm for whole larvae and 50μm for dissected tissues. All confocal images are projections except *Lectin-GalC1-T2A-Gal4*, *Cals-T2A-Gal4*, *CG6357-T2A-Gal4*, *Cpr47Eb-T2A-Gal4*, and *lectin-28A-T2A-Gal4*, which are slices.

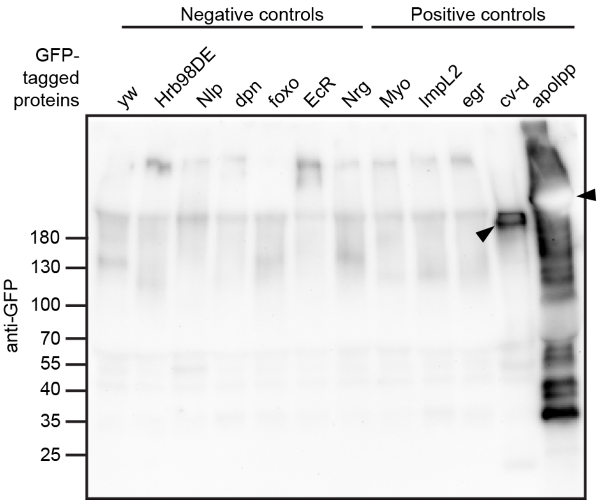

Supplementary Figure 21: Western detection of control GFP-tagged proteins in hemolymph. Western blot of 3rd instar larval hemolymph collected from GFP-tag lines. Negative controls are intracellular GFP-tagged proteins and positive controls are GFP-tagged known hemolymph proteins. Arrowheads indicate bands at the expected molecular weight (197.1 kDa = cv-d; 330 kDa = apoL1).

Supplementary Note 1 - Characterization of ER-localized TurboID to label secreted proteins in cultured *Drosophila* S2R+ cells

We previously labeled proteins in the secretory pathway using Myc-tagged promiscuous biotin ligase BirA*G3 in the ER (*Myc-BirA*G3-ER*)[1, 2, 3]. BirA*G3 (G3 = Generation 3) is a precursor to the engineered promiscuous biotin ligase TurboID (which has three additional mutations compared to G3), where TurboID has higher biotinylation activity than BirA*G3[2]. Therefore, we constructed updated plasmids to express ER-localized TurboID (*GFP-TurboID-ER*) (**Figure 1A**). ER localization is accomplished using an N-terminal 18aa secretion signal from *Drosophila* BiP, and a C-terminal KDEL ER retention signal. For comparison, we constructed plasmids to express a cytoplasmic TurboID (*GFP-TurboID*).

To rapidly test and characterize this TurboID labeling strategy, we first performed experiments in cultured *Drosophila* S2R+ cells, which are a widely used and well-characterized *Drosophila* cell culture model for genome-wide functional and proteomic studies[4, 5, 6]. Notably, this TurboID labeling approach has not been applied to any cultured *Drosophila* cell line, so we saw an opportunity to establish a resource that could benefit the broader community, even though this was not our primary objective.

To test if GFP-TurboID-ER labels proteins in the secretory compartment, we transfected TurboID DNA constructs into S2R+ cells grown in biotin-supplemented media, and visualized GFP and biotinylated proteins with confocal microscopy. GFP-TurboID-ER fluorescent signal was excluded from cell nuclei, enriched in a punctate pattern surrounding the nucleus, and overlapped with the ER protein Calnexin 99A (Cnx99A) (**Supplementary Figure 1**). Biotinylated proteins were similarly localized to the ER. In contrast, we detected GFP-TurboID and biotinylated proteins in the cytoplasm and nucleus. We observed similar results using our previous Myc-BirA*G3 Myc-BirA*G3-ER constructs (**Supplementary Figure 2**), indicating that our TurboID constructs are properly localized like prior BirA*G3 versions.

Next, we tested whether we could detect secreted biotinylated proteins in cell culture media supernatant from cells expressing GFP-TurboID-ER. To induce robust expression, we generated stable S2R+ cell lines expressing either GFP-TurboID or GFP-TurboID-ER under the control of a copper-inducible metallothionein (*MT*) promoter. After incubating cell lines with CuSO4 and excess biotin for 24 hours, we subjected cell pellets and cell media supernatant to SDS-PAGE, followed by western blotting using streptavidin-HRP to detect biotinylated proteins (**Supplementary Figure 3A-B)**. For *MT-GFP-TurboID-ER* cells, we detected a smear of biotinylated protein bands of different molecular weights from both cell pellets and cell media, suggesting that both intracellular and secreted proteins are being labeled. For *MT-GFP-TurboID* cells, we also saw a smear of biotinylated proteins from cell pellets, but with a different banding pattern compared to *MT-GFP-TurboID-ER*. Unlike *MT-GFP-TurboID-ER* cells, the biotinylated protein were barely detectible in the media from *MT-GFP-TurboID* cells, indicating minimal secretion of TurboID-labeled cytosolic proteins into the extracellular medium. In contrast, when probing for the TurboID fusion proteins themselves, we detected GFP-TurboID and GFP-TurboID-ER in cell pellets by anti-GFP westerns, and we additionally detected GFP-TurboID-ER in cell media supernatant, suggesting that the protein can be secreted or released. Similarly, we observed a band corresponding to the same molecular weight as GFP-TurboID-ER (~55 kDa) on streptavidin-HRP blots, suggesting that TurboID auto-biotinylates, which has been previously described[2]. We observed similar results using transfection of *MT-Myc-BirA*G3-ER* in S2R+ cells (**Supplementary Figure 3C-D**).

Next, we tested if GFP-TurboID-ER biotinylates known secreted proteins and if we could recover them from culture media supernatant. To accomplish this, we transfected S2R+ cells with HA-tagged versions of two secreted ligands, Spatzle (Spz) and Hedgehog (Hh), collected culture media, enriched biotinylated proteins on streptavidin beads, and detected the HA-tagged proteins by western blot. Whereas we observed both Spz-HA and Hh-HA in input samples, they were only detected after pulldowns when co-transfecting *MT-GFP-TurboID-ER* (**Supplementary Figure 4**).

To further confirm that GFP-TurboID-ER biotinylates secreted proteins, we used liquid chromatography-tandem mass spectrometry (LC-MS/MS) to identify labeled proteins in culture media from stable cell lines. Using streptavidin beads to enrich biotinylated proteins in culture media supernatant, we ran the eluted proteins on an SDS-PAGE gel and detected total proteins using a Coomassie stain. We observed a smear of protein bands from *MT-GFP-TurboID-ER* cell media, which were not present from negative control wild-type S2R+ cell media, including a prominent band at the molecular weight of auto-biotinylated TurboID (**Supplementary Figure 5A**). Similar results were observed using BirA*G3 expressing cells **(Supplementary Figure 5B)**. Unexpectedly, we also observed a smear from *MT-GFP-TurboID* cell media pulldowns. This is despite gentle harvesting and filtering

of cell media (see methods), suggesting that S2R+ cells undergo cell lysis during normal culture and/or unconventional secretion. To identify proteins labeled by GFP-TurboID or GFP-TurboID-ER, we cut out entire gel lanes, followed by in-gel digestion and LC-MS/MS. We identified 188 *Drosophila* proteins from *MT-GFP-TurboID* cell media and 72 from *MT-GFP-TurboID-ER* cell media (**Supplementary Figure 5C, Supplementary Data 1A**). Proteins that are predicted as extracellular, such as containing a signal sequence and/or a transmembrane domain, were highly enriched from GFP-TurboID-ER media (76%) relative to GFP-TurboID media (1%). These secreted proteins include signaling proteins, extracellular matrix proteins, chaperones, and uncharacterized proteins (**Supplementary Figure 5D**). We also identified GFP-TurboID in media from both cell lines, confirming the protein is released into the media (**Supplementary Data 1B**). Interestingly, we also identified cow proteins such as Complement C3, suggesting GFP-TurboID in media may label proteins in culture media derived from fetal bovine serum (FBS) (**Supplementary Data 1B**). Proteins identified in the media from *MT-GFP-TurboID* cells result from cell leakage or unconventional secretion of cytoplasmic contents. It is interesting and unexpected that more proteins were identified from *MT-GFP-TurboID* cells than *MT-GFP-TurboID-ER* cells. This may be due to several factors, including that cytoplasmic proteins are generally more abundant[7], GFP-TurboID may have higher enzymatic activity in the cytosol[2], and the expression level of GFP-TurboID protein may be higher in the stable cell line (**Supplementary Figure 3A**).

In summary, our experiments demonstrate that GFP-TurboID-ER biotinylates proteins in the secretory pathway of cultured fly cells, which can be recovered from the extracellular space by streptavidin-beads and identified by LC-MS/MS, supporting its utility for *in vivo* tissue-specific secretome profiling.

Supplementary Note 2 - Generating and characterizing a collection of transgenic fly lines for *in vivo* secretome labeling in 10 major tissue types

Following our encouraging results in cell culture, we set out to express TurboID-ER *in vivo* in a tissue-specific manner. To accomplish this, we generated transgenic flies that express GFP-TurboID-ER under the control of the *Gal4*/*UAS* binary expression system[8] (**Figure 1A**). We constructed 10 fly strains that each carry two transgenes, a *UAS-GFP-TurboID-ER* transgene and a tissue-specific *Gal4* transgene, which results in constitutive tissue-specific expression of TurboID-ER (**Supplementary Figure 6A**). This approach facilitates raising large numbers of developmentally synchronized flies with the desired genotype. *Gal4* lines were selected to represent most major non-overlapping tissues/organs (e.g. muscle, fat, neurons) and, when possible, express in all cell subtypes (e.g. pan-glial *repo-gal4*). We confirmed tissue-specific expression in each recombinant line by visualizing GFP fluorescence in 3rd instar larvae (**Supplementary Figure 6B**).

Next, we detected labeled proteins from isolated hemolymph using SDS-PAGE and streptavidin-HRP western blotting. We focused on larval rather than adult hemolymph because it is easier to collect in large quantities and is therefore more amenable to future large-scale experiments (see next results section). We collected hemolymph from 20 wandering 3rd instar larvae per genotype, after raising them on biotin-supplemented food for 24 hours, which we previously showed maximizes biotinylation[2]. As a negative control, we used hemolymph collected from *UAS-GFP-TurboID-ER* larvae (i.e. no Gal4) that were raised on biotin-supplemented food. This ligase- biotin+ control was chosen because TurboID can utilize endogenous biotin present in standard fly food for low level labeling[2], making ligase+ biotin- conditions an unreliable baseline. Similar ligase-, biotin+ controls have been used in prior *in vivo* proximity-labeling proteomic studies[1, 3]. We observed numerous biotinylated proteins in hemolymph from tissue-specific GFP-TurboID-ER strains (**Supplementary Figure 6C-D**). Importantly, the banding pattern and intensity of biotinylated proteins differed among the tissue-specific genotypes. For example, biotinylated proteins appeared as a smear of different molecular weights or as discrete protein bands, such as a prominent ~25 kDa band in hemolymph from oenocyte-labeling. Hemolymph from fat body-labeling gave by far the strongest signal compared to the other samples, agreeing with previous studies that the fat body (similar to human adipose and liver) is a major source of secreted hemolymph proteins[9]. To compensate for this signal disparity, we loaded 1/50th the amount of hemolymph from fat body-labeling samples in western blots, which was empirically determined (**Supplementary Figure 6E**). These results demonstrate that our *in vivo* TurboID-ER labeling approach enables detection of secreted proteins in larval hemolymph and suggests tissue-specific differences in the composition of the secretome.

All 10 tissue-specific TurboID-ER lines were viable, fertile, and morphologically normal, and have been stably propagated for years, including at the Bloomington *Drosophila* Stock Center. Notably, even ubiquitous, constitutive TurboID expression with continuous biotin supplementation throughout the fly lifespan (a worst-case scenario) does not cause detectable toxicity or morphological defects[2]. In contrast, our experiments use tissue-specific TurboID-ER expression with only 24hr biotin supplementation during the 3rd instar larval stage.

Consistent with this, fat body expression of BirA*G3-ER does not affect adult survival or climbing ability in flies[1], and body-wide BirA*G3-ER expression in mice yields viable and fertile animals with no obvious histopathology, ER stress, or cell death[3]. Together, these studies suggest that TurboID-ER-based proximity labeling is well tolerated *in vivo*, particularly under tissue-specific and temporally restricted expression conditions such as those used here.

Supplementary Note 3 - A *Drosophila* larval hemolymph collection protocol that is compatible with large-scale streptavidin pulldowns

Our standard hemolymph isolation protocol, which involves bleeding 20 larvae, typically yields 15μg of protein. However, large-scale streptavidin pulldowns for MS require at least 3mg of protein input to ensure sufficient material is recovered after enrichment[10]. In addition, we eventually aimed to perform quantitative MS analysis using biological triplicates for 10 tissue-specific labeling samples. Given our large number of samples and the substantial amount of hemolymph required, it became clear that our standard protocol was not scalable for this purpose. Therefore, we developed a protocol to isolate large amounts of purified hemolymph from 3rd instar larvae (**Supplementary Figure 7A**). First, we synchronized development using timed egg collections from each recombinant fly strain expressing *GFP-TurboID-ER*. After 4 days, we transferred larvae to biotin-supplemented food. After 24hrs, we collected ~400 3rd instar larvae and bled them in PBS. The crude diluted hemolymph was then filtered and centrifuged to remove larvae, tissue debris, and cellular fragments. Finally, we passed filtered cell-free hemolymph through a 3 kDa centrifuge filter to concentrate the sample and remove free biotin, yielding ~7-10 mg of purified hemolymph.

Next, we performed pilot streptavidin pulldowns and label-free LC-MS/MS using hemolymph isolated with our large-scale procedure (**Supplementary Figure 7B**). Our goal was to evaluate protein identification using our combined genetic and biochemical strategy, specifically to assess signal quality (i.e. detection of known or predicted secreted proteins), extent of background protein contamination, and the degree of tissue specificity. We focused on tissues that gave strong signal by western blot, including muscle, fat body, gut enterocytes, glia, and oenocytes, and only used single biological replicates at this stage. We purified hemolymph from larvae with tissue-specific labeling, enriched biotinylated proteins on streptavidin beads, and processed samples for LC-MS/MS by either in-gel (whole lane and prominent Coomassie-stained bands) or on-bead digestion (**Supplementary Figure 7C**). To help interpret our results, we also collected hemolymph from wild-type larvae and processed it using in-gel digestion of prominent Coomassie-stained bands and in-solution digestion (**Supplementary Figure 7D**).

In total, we identified 213 proteins from three tissue-labeling (with pulldown) experiments and 185 proteins from two wild-type hemolymph (no pulldown) experiments, with 56 overlapping proteins (**Supplementary Figure 7B, Supplementary Data 2A-B).** Over 50% of the proteins identified are predicted secreted (**Supplementary Figure 7E, Supplementary Data 2A**). The most abundant proteins identified in unlabeled hemolymph are the apolipoprotein Apolipophorin (Apolpp) and the larval serum proteins Lsp1β, Lsp1γ, Lsp1α, Lsp2, which is consistent with previous studies[9, 11, 12] (**Supplementary Figure 7D-E**). We readily identified these same abundant proteins in tissue-labeling samples, including other known hemolymph proteins such as ferritins, prophenoloxidases, and extracellular matrix proteins (**Supplementary Figure 7C,E, Supplementary Data 2A**)[9, 13, 14, 15].

Many proteins from tissue-labeling samples exhibited tissue-specific enrichment. For example, Ferritin 1 heavy chain homologue (Fer1HCH) and Ferritin 2 light chain homologue (Fer2LCH), which together form the major Ferritin complex and are known to be secreted from the gut[16], were the top proteins identified from a prominent ~25 kDa gel band that was specific to gut enterocyte labeling (**Supplementary Figure 7C, Supplementary Data 2A**). In addition, Lectin-22c, an uncharacterized C-type lectin protein, was the top protein identified from a prominent ~35 kDa gel band specific to oenocyte labeling (**Supplementary Figure 7C, Supplementary Data 2A**). We defined a set of 48 tissue-enriched proteins by comparing the spectral counts of proteins from whole gel lanes (**Supplementary Figure 7F, Supplementary Data 2A**). In many cases, identified proteins originated from the expected tissue, such as Viking (Vkg) from fat body[13, 17] and Odorant-binding protein 44a (Obp44a) from glia[18]. We also identified known hemolymph proteins from unexpected tissues, such as the fat body proteins apolpp, Lsp1β, and Gelsolin (Gel)[17, 19] from muscle-labeling samples, and the fat body proteins Crossveinless d (Cv-d), Imaginal disc growth factor 4 (Idgf4), and Transferrin 1 (Tsf1)[20, 21, 22] from glia-labeled samples. We also identified uncharacterized tissue-specific secreted proteins, such as CG6867 from muscle, CG5080 from glia, Lectin-22c from oenocytes, and CG2233 from fat body. Finally, we identified proteins in tissue-labeling samples that were missing from wild-type hemolymph samples (e.g. Lectin-22c, Cv-d, Bangles and beads (Bnb), and

CG5080) (**Supplementary Data 2A**), suggesting that tissue-labeling and pulldown helps enrich low abundance hemolymph proteins.

Our data also highlight unwanted background proteins binding to streptavidin beads. For example, one tissue labeling and pulldown experiment involved parallel processing of 5 tissue-labeling lines (*X-Gal4*, *UAS-GFP-TurboID-ER*) and one non-labeling line (*UAS-GFP-TurboID-ER*, no Gal4), identifying eluted proteins by whole lane gel digestion (**Supplementary Figure 7B, Supplementary Data 2A**). All 21 proteins identified in the control sample were present in at least one of the experimental labeling samples (**Supplementary Figure 7G, Supplementary Data 2B**), including the top 5 most abundant proteins in wild-type hemolymph (Apolpp, Lsp1β, Lsp1γ, Lsp1α, and Lsp2), as well as Acetyl-CoA carboxylase (ACC) and Pyruvate carboxylase (Pcb), which are naturally biotinylated proteins[23, 24]. Furthermore, ACC and Pcb were not identified from wild-type hemolymph (**Supplementary Figure 7H, Supplementary Data 2C**). Therefore, naturally biotinylated ACC and Pcb may be present in circulation, or released from tissue during larval bleeding, and bind to streptavidin beads, whereas abundant hemolymph proteins may bind beads non-specifically. This phenomenon of non-biotinylated proteins binding to streptavidin beads, despite extensive and harsh bead washes, has been observed by other studies[25, 26].

In summary, our tissue-specific TurboID-ER fly strains effectively label and enrich secreted biotinylated proteins in the hemolymph. These pilot results validate the logic of our approach and provide a strong rationale to scale up for quantitative MS analysis with biological replicates across all 10 tissue types. However, to maximize the impact of these quantitative MS experiments, we first addressed the issue of background proteins binding non-specifically to streptavidin beads.

Supplementary Note 4 - Reduced non-specific binding to streptavidin beads with more stringent washing

Non-specific binding of non-biotinylated abundant hemolymph proteins to the beads creates two problems for tissue secretome analysis. 1) It reduces the chances of identifying tissue-secreted biotinylated proteins, particularly those that are low abundance and 2) it prevents a proper analysis of the tissue of origin of abundant hemolymph proteins.

Streptavidin pulldown of wild-type hemolymph revealed a prominent band between 70-100 kDa on SDS-PAGE gels (**Supplementary Figure 7C**), and LC-MS/MS of the entire gel lane identified all four Larval Serum Proteins (LSPs) (**Supplementary Data 2A**). Indeed, LC-MS/MS of a similar molecular weight band from wild-type hemolymph (**Supplementary Figure 7D**) revealed that it was predominantly composed of all four LSPs (**Supplementary Data 2A**). Therefore, using our current pulldown protocol, which is based on previous studies[2], we hypothesize that LSPs and other abundant proteins in hemolymph non-specifically bind to streptavidin beads.

To test whether more stringent bead washing could reduce this non-specific binding, we applied a series of harsher wash conditions. 2M Urea is traditionally used as a harsh washing step because it denatures proteins, so we tested increasing Urea concentrations. In addition, others have used SDS in streptavidin bead washing steps[25, 27, 28], therefore we tested Urea washes with and without SDS (**Supplementary Figure 8A**). To monitor non-specific binding to the beads, we detected LSPs as a 70-100 kDa protein band on silver-stained gels and by western blotting using anti-LSP-1γ (**Supplementary Figure 8A**), which is cross-reactive with all three LSP-1 proteins[29]. Our results show that higher concentrations of Urea, as well as adding SDS, reduced non-specific binding of LSPs below the level of detection (**Supplementary Figure 3A**).

Next, we tested if more stringent washing had a negative impact on the binding of biotinylated hemolymph proteins to the beads. As described previously (**Supplementary Figure 7B**), we collected hemolymph from larvae with fat body labeling, performed streptavidin pulldowns, eluted biotinylated proteins, and detected biotinylated proteins on western blots with streptavidin-HRP. Using a panel of Urea and SDS bead washing conditions, there were no obvious differences in the intensity or banding pattern of biotinylated proteins (**Supplementary Figure 8B**). Furthermore, the prominent 70-100 kDa band corresponding to LSPs appears similar in intensity for all bead washing conditions. Therefore, we conclude that harsher bead washing reduces non-specific binding but does not reduce retention of biotinylated proteins on the beads. Thus, we adopted 4M Urea + 2% SDS in future bead washing steps.

Supplementary Note 5 - GFP-TurboID-ER sequence

MKLCILLAVVAFVGLSLGESKGEELFTGVVPILVELDGDVNGHKFSVRGEGEGDATNGKLTLKFICTTGKLPVPWP
TLVTTLTYGVQCFSRYPDHMKQHDFFKSAMPEGYVQERTISFKDDGTYKTRAEVKFEGDTLVNRIELKGIDFKED

GNILGHKLEYNFNSHNVYITADKQKNGIKANFKIRHNVEDGSVQLADHYQQNTPIGDGPVLLPDNHYLSTQSVLSK
DPNEKRDHMVLLEFVTAAGITLGMDELYKTGGSGGSGGSGGSGGSGGSGGKDNTVPLKLIALLANGEFHSGEQL
GETLGMSRAAINKHIQTLRDWGVDVFTVPGKGYSLPEPIPLLNAKQILGQLDGGSVAVLPVVDSTNQYLLDRIGEL
KSGDACIAEYQQAGRGSRGRKWFSPFGANLYLSMFWRLKRGPAAIGLGPVIGIVMAEALRKLGADKVRVKWPND
LYLQDRKLAGILVELAGITGDAAQIVIGAGINVAMRRVEESVVNQGWITLQEAGINLDRNTLAATLIRELRAALELFE
QEGLAPYLPRWEKLDNFINRPVKLIIGDKEIFGISRGIDKQGALLLEQDGVIKPWMGGEISLRSAEKKDEL

References:

1. Droujinine IA, *et al.* Proteomics of protein trafficking by in vivo tissue-specific labeling. *Nat Commun* **12**, 2382 (2021).

2. Branon TC, *et al.* Efficient proximity labeling in living cells and organisms with TurboID. *Nat Biotechnol* **36**, 880-887 (2018).

3. Yang R, *et al.* A genetic model for in vivo proximity labelling of the mammalian secretome. *Open Biol* **12**, 220149 (2022).

4. Viswanatha R, Li Z, Hu Y, Perrimon N. Pooled genome-wide CRISPR screening for basal and context-specific fitness gene essentiality in Drosophila cells. *Elife* **7**, (2018).

5. Chen CL, *et al.* Proteomic mapping in live Drosophila tissues using an engineered ascorbate peroxidase. *Proc Natl Acad Sci U S A* **112**, 12093-12098 (2015).

6. Yanagawa S, Lee JS, Ishimoto A. Identification and characterization of a novel line of Drosophila Schneider S2 cells that respond to wingless signaling. *J Biol Chem* **273**, 32353-32359 (1998).

7. Kuhn PH, *et al.* Secretome protein enrichment identifies physiological BACE1 protease substrates in neurons. *EMBO J* **31**, 3157-3168 (2012).

8. Brand AH, Perrimon N. Targeted gene expression as a means of altering cell fates and generating dominant phenotypes. *Development* **118**, 401-415 (1993).

9. Handke B, *et al.* The hemolymph proteome of fed and starved Drosophila larvae. *PLoS One* **8**, e67208 (2013).

10. Hung V, *et al.* Spatially resolved proteomic mapping in living cells with the engineered peroxidase APEX2. *Nat Protoc* **11**, 456-475 (2016).

11. Wolfe J, Akam ME, Roberts DB. Biochemical and immunological studies on larval serum protein 1, the major haemolymph protein of Drosophila melanogaster third-instar larvae. *Eur J Biochem* **79**, 47-53 (1977).

12. Akam ME, Roberts DB, Wolfe J. Drosophila hemolymph proteins: purification, characterization, and genetic mapping of larval serum protein 2 in D. melanogaster. *Biochem Genet* **16**, 101-119 (1978).

13. Pastor-Pareja JC, Xu T. Shaping cells and organs in Drosophila by opposing roles of fat body-secreted Collagen IV and perlecan. *Dev Cell* **21**, 245-256 (2011).

14. Binggeli O, Neyen C, Poidevin M, Lemaitre B. Prophenoloxidase activation is required for survival to microbial infections in Drosophila. *PLoS Pathog* **10**, e1004067 (2014).

15. Gonzalez-Morales N, Mendoza-Ortiz MA, Blowes LM, Missirlis F, Riesgo-Escovar JR. Ferritin Is Required in Multiple Tissues during Drosophila melanogaster Development. *PLoS One* **10**, e0133499 (2015).

16. Rosas-Arellano A, *et al.* Ferritin Assembly in Enterocytes of Drosophila melanogaster. *Int J Mol Sci* **17**, 27 (2016).

17. Deutsch J, Laval M, Lepesant JA, Maschat F, Pourrain F, Rat L. Larval fat body-specific gene expression in D. melanogaster. *Dev Genet* **10**, 220-231 (1989).

18. Yin J*, et al.* Glia-derived noncanonical fatty acid binding protein modulates brain lipid storage and clearance. *Sci Adv* **11**, eadv2902 (2025).

19. Stella MC, Schauerte H, Straub KL, Leptin M. Identification of secreted and cytosolic gelsolin in Drosophila. *J Cell Biol* **125**, 607-616 (1994).

20. Geiser DL, Winzerling JJ. Insect transferrins: multifunctional proteins. *Biochim Biophys Acta* **1820**, 437-451 (2012).

21. Kawamura K, Shibata T, Saget O, Peel D, Bryant PJ. A new family of growth factors produced by the fat body and active on Drosophila imaginal disc cells. *Development* **126**, 211-219 (1999).

22. Palm W*, et al.* Lipoproteins in Drosophila melanogaster--assembly, function, and influence on tissue lipid composition. *PLoS Genet* **8**, e1002828 (2012).

23. Ramirez J*, et al.* Proteomic Analysis of the Ubiquitin Landscape in the Drosophila Embryonic Nervous System and the Adult Photoreceptor Cells. *PLoS One* **10**, e0139083 (2015).

24. Larochelle M, Bergeron D, Arcand B, Bachand F. Proximity-dependent biotinylation mediated by TurboID to identify protein-protein interaction networks in yeast. *J Cell Sci* **132**, (2019).

25. Grismer TS*, et al.* Workflow enhancement of TurboID-mediated proximity labeling for SPY signaling network mapping. *bioRxiv*, (2024).

26. Mair A, Xu SL, Branon TC, Ting AY, Bergmann DC. Proximity labeling of protein complexes and cell-type-specific organellar proteomes in Arabidopsis enabled by TurboID. *Elife* **8**, (2019).

27. Cheah JS, Yamada S. A simple elution strategy for biotinylated proteins bound to streptavidin conjugated beads using excess biotin and heat. *Biochem Biophys Res Commun* **493**, 1522-1527 (2017).

28. De Munter S*, et al.* Split-BioID: a proximity biotinylation assay for dimerization-dependent protein interactions. *FEBS Lett* **591**, 415-424 (2017).

29. Burmester T, Antoniewski C, Lepesant JA. Ecdysone-regulation of synthesis and processing of fat body protein 1, the larval serum protein receptor of Drosophila melanogaster. *Eur J Biochem* **262**, 49-55 (1999).
