# Supplementary material for: Multi-omic mapping of Drosophila protein secretomes reveals tissue-specific origins and inter-organ trafficking
Source: Nat Commun. 2026 Apr 20;17:5425. doi: 10.1038/s41467-026-71763-8 (PMC13280006; doi:10.1038/s41467-026-71763-8)
Supplement: Supplementary file 10 — Reporting Summary [file 41467_2026_71763_MOESM10_ESM.pdf]

nature portfolio

Corresponding author(s): Justin Bosch

Last updated by author(s): Feb 21, 2026

Reporting Summary

Nature Portfolio wishes to improve the reproducibility of the work that we publish. This form provides structure for consistency and transparency in reporting. For further information on Nature Portfolio policies, see our Editorial Policies and the Editorial Policy Checklist.

Statistics

For all statistical analyses, confirm that the following items are present in the figure legend, table legend, main text, or Methods section.

n/a	Confirmed	
☐	☒	The exact sample size (*n*) for each experimental group/condition, given as a discrete number and unit of measurement
☒	☐	A statement on whether measurements were taken from distinct samples or whether the same sample was measured repeatedly
☐	☒	The statistical test(s) used AND whether they are one- or two-sided<br>*Only common tests should be described solely by name; describe more complex techniques in the Methods section.*
☒	☐	A description of all covariates tested
☐	☒	A description of any assumptions or corrections, such as tests of normality and adjustment for multiple comparisons
☐	☒	A full description of the statistical parameters including central tendency (e.g. means) or other basic estimates (e.g. regression coefficient) AND variation (e.g. standard deviation) or associated estimates of uncertainty (e.g. confidence intervals)
☐	☒	For null hypothesis testing, the test statistic (e.g. *F*, *t*, *r*) with confidence intervals, effect sizes, degrees of freedom and *P* value noted<br>*Give P values as exact values whenever suitable.*
☒	☐	For Bayesian analysis, information on the choice of priors and Markov chain Monte Carlo settings
☒	☐	For hierarchical and complex designs, identification of the appropriate level for tests and full reporting of outcomes
☒	☐	Estimates of effect sizes (e.g. Cohen's *d*, Pearson's *r*), indicating how they were calculated

Our web collection on statistics for biologists contains articles on many of the points above.

Software and code

Policy information about availability of computer code

Data collection	Zen Blue (Zeiss) for Axio Zoom V16, Image Lab software for ChemiDoc MP Imaging System (Bio-Rad), ZEN Black for confocal imaging LSM780 (Zeiss), SH800Z Cell Sorter Software (Sony)
Data analysis	Mascot (Matrix Science, v2.7); Scaffold Q+S (Proteome Software, v5.0); Spectrum Mill (Agilent Technologies, v6.1 pre-release); R (v4.1.0), limma (v3.48.3); PaxDB (v5.0); PANGEA (v2beta); TM4 (v4.7.4); gprofiler2 (v0.2.1); Cytoscape (v3.8.2) with the EnrichmentMap app; GLAD (v1.0); DGET (v2.0.1); DRscDB (PMID: 33995899); FlyPhoneDB2 (v1.0); Cell Ranger (10x Genomics, v7.1.0); DecontX (v1.0.0); DoubletFinder (v3); Harmony (v0.1); Python (v3.13.2); SignalP 6.0; SignalP 5.0; TMHMM 2.0; DeepTMHMM 1.0; DeepLoc 2.0; DIOPT Ortholog Finder (v10.0); Human Protein Atlas (PMID: 31772123); Find CRISPRs tool (v3.1.0); Fiji (v2.14); Prism (v10.4.1, Graphpad).

For manuscripts utilizing custom algorithms or software that are central to the research but not yet described in published literature, software must be made available to editors and reviewers. We strongly encourage code deposition in a community repository (e.g. GitHub). See the Nature Portfolio guidelines for submitting code & software for further information.

Data

Policy information about availability of data

All manuscripts must include a data availability statement. This statement should provide the following information, where applicable:

- Accession codes, unique identifiers, or web links for publicly available datasets
- A description of any restrictions on data availability
- For clinical datasets or third party data, please ensure that the statement adheres to our policy

Plasmids are available from Addgene and the Drosophila Genomic Resource Center (DGRC). Cell lines are available from the DGRC. Fly lines are available from the Bloomington Drosophila Stock Center (BDSC).

Raw mass spectrometry data for the pilot secretome experiments from S2R+ and 3rd instar larvae is available from FigShare. https://doi.org/10.6084/m9.figshare.31286095

Raw mass spectrometry data for the Tissue Secretome Map (TMT experiment) and Blood proteome (Label-free experiment) have been deposited in the public proteomics repository MassIVE and are accessible at accession number MSV000098658. ftp://massive-ftp.ucsd.edu/v10/MSV000098658/

Raw snRNA-seq is available from the GEO at accession number GSE302679. https://www.ncbi.nlm.nih.gov/geo/query/acc.cgi?acc=GSE302679

snRNA-seq data can also be accessed on web-based portal: https://www.flyrnai.org/scRNA/body_larvae/

Raw graph data, western blots, and gels are in Source Data.

Research involving human participants, their data, or biological material

Policy information about studies with human participants or human data. See also policy information about sex, gender (identity/presentation), and sexual orientation and race, ethnicity and racism.

Reporting on sex and gender	N/A
Reporting on race, ethnicity, or other socially relevant groupings	N/A
Population characteristics	N/A
Recruitment	N/A
Ethics oversight	N/A

Note that full information on the approval of the study protocol must also be provided in the manuscript.

Field-specific reporting

Please select the one below that is the best fit for your research. If you are not sure, read the appropriate sections before making your selection.

[x] Life sciences   [ ] Behavioural & social sciences   [ ] Ecological, evolutionary & environmental sciences

For a reference copy of the document with all sections, see nature.com/documents/nr-reporting-summary-flat.pdf

Life sciences study design

All studies must disclose on these points even when the disclosure is negative.

Sample size	No statistical method was used to predetermine sample sizes. Sample sizes were determined based on pilot experiments and practical feasibility.
Data exclusions	No data were excluded from the results.
Replication	For confocal imaging of S2R+ cells, at least three fields of >100 cells were collected, and at least three transfected cells shown in figures. Experiments involving SDS-PAGE and western blots were reliably performed at least twice independently. Pilot larval blood proteomic data was verified using separate protocols (streptavidin pulldown vs. raw blood) (on-bead digestion vs whole lane gel digestion). Quantitative larval tissue-secretome proteomics was performed in triplicate. GSEA analysis was performed using two independent methods (PANGEA, g:Profiler). For fluorescence stereomicroscopy, at least three whole larvae were imaged by fluorescence stereomicroscopy. For confocal microscopy, at least 6 larvae were dissected and stained, at least 6 replicate tissue samples (e.g. 6 or more imaginal discs) were mounted on slides, and at least 3 tissue samples were imaged. snRNA-seq cluster gene expression and fly knock-in expression and localization data served to verify the proteomics results. Pilot larval tissue-secretome and raw blood proteome results also verified large-scale quantitative proteomics results. Each TMT plex experiment contained a biological replicate of the negative control genotype (UAS-GFP-TurboID-ER), and an internal reference of raw hemolymph from yw larvae. To verify potential knock-in fly lines, at least two founder lines (when available) were genotyped to confirm the knock-in. All attempts at replication were successful, except the following (all discussed in the results and discussion): 1) Obp56e-

	T2A-Gal4 was not expressed in the same cell types that Obp56e was presumed secreted from. 2) A minority of GFP-tagged proteins (positive controls and experimentals) were detected in the blood by anti-GFP western blots.
Randomization	No randomization methods were used because this was not applicable for our experiments. Fields of view of S2R+ cells were selected based on pre-selected region in a consistent location within every well. A fixed number of larvae or adults of the desired genotypes were selected without bias from a developmentally synchronized cohort used for blood collection, imaging, or genetic crosses. All dissected tissues mounted on slides were examined by eye and undamaged representative tissues were selected for imaging.
Blinding	Our experiments did not include subjective measurements and therefore did not require blinding.

Reporting for specific materials, systems and methods

We require information from authors about some types of materials, experimental systems and methods used in many studies. Here, indicate whether each material, system or method listed is relevant to your study. If you are not sure if a list item applies to your research, read the appropriate section before selecting a response.

Materials & experimental systems

n/a	Involved in the study
☐	☒ Antibodies
☐	☒ Eukaryotic cell lines
☒	☐ Palaeontology and archaeology
☐	☒ Animals and other organisms
☒	☐ Clinical data
☒	☐ Dual use research of concern
☒	☐ Plants

Methods

n/a	Involved in the study
☒	☐ ChIP-seq
☒	☐ Flow cytometry
☒	☐ MRI-based neuroimaging

Antibodies

Antibodies used	mouse anti-Cnx99A (1:10, DSHB, Cnx99A 6-2-1)<br>chicken anti-GFP (1:1000, AVES labs GFP1020)<br>goat anti-chicken 488 (1:500, Thermo Fisher, A-11039)<br>donkey anti-mouse 488 (1:500, Molecular Probes A-21202)<br>rabbit anti-myc (1:250, Cell Signaling 71D10)<br>donkey anti-rabbit 488 (1:500, Molecular Probes A21206)<br>chicken anti-GFP (1:1000, AVES labs GFP1020)<br>goat anti-chicken 488 (1:500, Thermo Fisher, A-11039)<br>Rhodamine Anti-Actin (1:10,000, Biorad 12004163)<br>rabbit anti-GFP (1:5,000, Invitrogen a6455)<br>goat anti-rabbit 800 (1:5,000, Thermo Fisher, A32730)<br>chicken anti-HA (1:5,000, Aves, ET-HA100)<br>goat anti-chicken-HRP (1:1,000, Sigma, SAB3700199)<br>rabbit anti-LSP-1γ (1:5000) (PMID: 10231363)<br>rabbit anti-myc (1:2000, Cell Signaling 71D10)<br>donkey anti-rabbit-HRP (1:3000, Amersham NA934)
Validation	mouse anti-Cnx99A: https://dshb.biology.uiowa.edu/Cnx99A-6-2-1<br>chicken anti-GFP: https://www.antibodiesinc.com/products/anti-green-fluorescent-protein-antibody-gfp<br>rabbit anti-myc: https://www.cellsignal.com/products/primary-antibodies/myc-tag-71d10-rabbit-monoclonal-antibody/2278<br>Rhodamine Anti-Actin: https://www.bio-rad.com/en-us/sku/12004163-hfab-rhodamine-anti-actin-primary-antibody-200-ul?ID=12004163<br>rabbit anti-GFP: https://www.thermofisher.com/antibody/product/GFP-Antibody-Polyclonal/A-6455<br>chicken anti-HA: https://www.antibodiesinc.com/products/anti-ha-epitope-tag-antibody-et-ha100<br>rabbit anti-LSP-1γ: PMID: 10231363

Eukaryotic cell lines

Policy information about cell lines and Sex and Gender in Research

Cell line source(s)	S2R+ cells were originally from Dr. Schneider and have been passaged in Perrimon lab and DRSC at Harvard Medical School for decades.
Authentication	The S2R+ cell line was obtained from the Drosophila RNAi Screening Center/Transgenic RNAi Project Functional Genomics Resources and Drosophila Research & Screening Center-Biomedical Technology Research Resource at Harvard Medical School. It has distinct morphology that can be relied on for quality control in routine passages. Moreover, it has been molecularly validated by transposable elements DNA sequencing (https://doi.org/10.1093/g3journal/jkab403).

Mycoplasma contamination	Contamination with mycoplasma is not an issue for Drosophila cultured cells. We regularly monitored the cultures for contamination by bacteria or fungus, and did not observe any signs of microbial contamination in cultures used in this study.
Commonly misidentified lines (See ICLAC register)	N/A

Animals and other research organisms

Policy information about studies involving animals; ARRIVE guidelines recommended for reporting animal research, and Sex and Gender in Research

Laboratory animals	Drosophila melanogaster.
Wild animals	No wild animals were used in in this study.
Reporting on sex	All experiments were performed on mixtures of male and female 3rd instar larvae sibling progeny at ~50% ratios, except for snRNA-seq experiments in which exactly 50% mixtures of males and female larvae were used.
Field-collected samples	None.
Ethics oversight	No ethical approval was required for studies on on Drosophila melanogaster.

Note that full information on the approval of the study protocol must also be provided in the manuscript.

Plants

Seed stocks	N/A
Novel plant genotypes	N/A
Authentication	N/A
