# Supplementary material for: Multi-omic mapping of Drosophila protein secretomes reveals tissue-specific origins and inter-organ trafficking
Source: Nat Commun. 2026 Apr 20;17:5425. doi: 10.1038/s41467-026-71763-8 (PMC13280006; doi:10.1038/s41467-026-71763-8)
Supplement: Supplementary file 11 — Transparent Peer Review file [file 41467_2026_71763_MOESM11_ESM.pdf]

nature portfolio

Peer Review File

Multi-omic mapping of *Drosophila* protein secretomes reveals tissue-specific origins and inter-organ trafficking

Corresponding Author: Dr Justin Bosch

This file contains all reviewer reports in order by version, followed by all author rebuttals in order by version.

Version 0:

Reviewer comments:

Reviewer #1

(Remarks to the Author)
This manuscript addresses a timely and technically challenging question: the identification of cell- and tissue-specific secreted proteins and their roles in inter-organ communication. Secretome mapping remains limited by available methodologies, and the ability to trace tissue-specific origins of circulating proteins is of considerable diagnostic and therapeutic relevance.
The authors present an ambitious study that aims to map the tissue-of-origin of secreted proteins in Drosophila melanogaster larvae at an unprecedented scale. They employ TurboID proximity labeling, mass spectrometry, single-nucleus RNA sequencing (snRNA-seq), and CRISPR gene editing to generate an atlas of proteins present in the larval hemolymph and assign them to ten major tissue types. They identify 535 tissue-specific hemolymph proteins and observe a substantial correspondence between these results and whole-animal snRNA-seq datasets. The work is conceptually strong and leverages a simple and genetically tractable in vivo system to infer inter-organ communication pathways.

Major Comments
1. Balance of method development vs. biological insight
A substantial proportion of the manuscript is devoted to method optimization (TurboID expression strategies, ER labeling validation, optimization of bead pulldown conditions, etc.). While these steps are undoubtedly essential and the authors are well positioned as experts in proximity labeling, the emphasis on optimization detracts from the stated goal of generating a comprehensive tissue-specific secretome map.
These methodological details would be more appropriate as expanded Supplementary Information. Reducing their prominence in the main text would allow the biological insights—particularly the identification, classification, and functional interpretation of tissue-derived secreted proteins—to take center stage.

2. Validation and characterization of the genetic lines
The authors state that the ten tissue-specific TurboID lines exhibit normal phenotypes, but no supporting data are shown. Given that aberrant expression or ER-targeted enzymes could impact development or secretion pathways, phenotypic validation is essential for interpreting the secretome profiles. I recommend including data (e.g., viability, morphology, developmental timing) or citing established characterizations.

3. Unexpectedly low number of high-confidence secreted proteins
The authors identify 2,543 proteins by LC-MS/MS in total but conclude that only 535 represent tissue-specific secreted factors. This represents a modest fraction (~20%), which seems low given that a single cell line in vitro produced 72 secreted proteins, and that the global hemolymph proteome comprises over 4,500 proteins.
This raises questions about:
• The stringency of the filtering criteria
• Potential underestimation due to TurboID efficiency, labeling window, or tissue-specific expression differences
• The possibility that some true secreted proteins were excluded due to conservative thresholds
Clarifying the rationale and sensitivity/specificity tradeoffs in defining “high-confidence” secreted proteins would strengthen the manuscript. It may also be appropriate to discuss whether the 535 proteins represent a lower bound.

What is captured in their detection method?
Are small polar proteins among the peptides detected in the MS/MS?

Can such proteins be validated to be present by the authors detection method by plotting the amount of small polar proteins present in the Drosophila proteome as a distribution plot against what is found in the MS/MS analysis of both the full proteome and the proteins expressed from specific tissues (TurboID tagged and biotin purified) ? A pie diagram would suffice.

Following my initial comment and regarding the number of proteins the authors detect (535 proteins) compared to the total amount of proteins in the plasma they detect (>4500 proteins): It is my understanding that the TMT - MS/MS analysis is done in a way so that any proteins that are not expressed in all the specific tissues are excluded, which if true is producing a lot of 'false negative' proteins. It is not clear in the manuscript if the zero values are excluded from the TurboID labelled samples. Please mention is the values are imputed or not. Zero values are difficult to deal with, but this should maybe be commented on as a limitation.

4. Limited functional analysis of the identified secreted proteins
Many of the 535 proteins lack prior annotations as secreted factors, which highlights the novelty of the dataset. However, the manuscript devotes relatively little space to functional interpretation, GO/pathway analysis, evolutionary conservation, or connections to known signaling pathways. This is a missed opportunity for deeper biological insight.
Greater emphasis on:
• Functional categories within tissue-specific secretomes
• Enrichment analyses (and their biological implications)
• Homology to human proteins and translational relevance
would significantly increase the impact of the study.

5. Discrepancy in GFP-knock-in validation
When the authors perform GFP knock-ins to visualize selected tissue-specific secreted proteins, only a small subset is detectable in the hemolymph. This discrepancy warrants more discussion. Does GFP tagging interfere with secretion, folding, or stability? Are expression levels too low at the larval stage?
Addressing this point is important for validating the platform and for interpreting the absence of GFP signal as biological rather than technical.

6. Limited demonstration of inter-organ communication
One of the stated aims is to identify inter-organ communication factors. However, only two proteins are ultimately highlighted as examples. While the resource remains valuable, the biological conclusions regarding communication networks appear modest. This should be more clearly acknowledged in the Discussion.

7. Developmental-stage limitation
The authors use Drosophila larvae, which may limit the relevance of the secretome to adult physiology and known inter-organ axes (e.g., fat body–muscle, intestine–brain). This developmental constraint should be addressed more explicitly, especially in relation to claims of comprehensiveness and translational relevance.

Minor Comments
1. Clarify S2R+ cells.
Please provide a brief description or reference for Drosophila S2R+ cells, as not all readers may be familiar with their properties and suitability for ER-TurboID validation.
2. Figure 1 ER-localization validation.
Orthogonal confocal projections would help demonstrate ER specificity more convincingly.
3. Negative control conditions.
A negative control using flies raised without supplemental biotin would help quantify background biotinylation.
4. Supplementary Fig. 7 – GO analysis.
The biological relevance of the top two pathways should be explained, particularly in relation to secretome function.
5. Functional analysis.
Additional description or discussion of functional enrichment results would be beneficial.

Assessment of Data, Methods, and Presentation
The methodology is generally sound and employs state-of-the-art tools for proximity labeling and proteomics. The mass spectrometry data appear of high quality, and the integration with snRNA-seq is a strong asset. Figures are clearly presented, although some could be reorganized to emphasize biological results over methodological detail.
I do not identify concerns about statistical validity, but the manuscript would benefit from clearer justification of filtering criteria and explicit discussion of detection thresholds. All referenced datasets appear appropriate, though I encourage the authors to ensure full availability of raw and processed data for community use.

Significance
This work represents a substantial technical effort toward mapping tissue-specific secretomes in vivo. While the resource is valuable and the methodological platform may be widely useful, the biological conclusions are relatively limited, and the secretome map is incomplete both in developmental scope and functional interpretation.
Nevertheless, the study constitutes an important step in enabling scalable, genetically tractable secretome profiling and may serve as a basis for future work in both flies and higher organisms.

Reviewer #2

(Remarks to the Author)
I co-reviewed this manuscript with one of the reviewers who provided the listed reports. This is part of the Nature Communications initiative to facilitate training in peer review and to provide appropriate recognition for Early Career Researchers who co-review manuscripts.

Reviewer #4

(Remarks to the Author)
This manuscript by Bosch et al employed TurboID-based proximity labeling to characterize the secretomes in Drosophila third instar larvae. They identified identified 535 circulating secreted proteins from the larval blood. Then they performed snRNA-seq of the whole larvae to validate these proteins' origin tissues. They also generated knock-in fly lines using CRISPR and revealed proteins that are synthesized in one tissue and localized to another.

Overall, this interesting and comprehensive study establishes a novel platform for secretome discovery in Drosophila. It provides very useful resources to study secreting proteins from larval stages and also opens the possibility to systematic characterize adult secretomes for inter-organ communication. I don't have major concerns, but have several suggestions to further improve this manuscript.

General comments:
Figure 1 related
a. Could the authors explain why more proteins were detected from the MT-GFP-TurboID than MT-GFP-TurboID-ER cell media in Fig 1F?
b. Since many proteins localize to the ER (primary site for translation), is it possible that many of the unpredicted secreted proteins in the GFP-TurboID-ER are non-specific biotinylation? Or could this have been from the gel lane extraction? Maybe elaborating on this result in the text would be helpful. It may also be possible that secreted proteins could be rapidly taken up by the cell that just secreted them. Is there sufficient time for this to occur?

Figure 2 related
a. Fig 2G: 213 proteins are detected from tissue specific labeling sample with streptavidin pulldown, and 185 proteins were detected from wild type control without pulldown. How many of these two groups are overlapped? Also, an important control can be from wild type with streptavidin pulldown. This control will help validate the fidelity of tissue-labeling and to identify background noise. It seems the authors have this data (line 313/314) but didn't compare this control with these 213 and 185 proteins. Additional analysis will be appreciated.
b. Since different tissue drivers likely have different expression levels (based on snRNA-seq, repo is lower whereas apolpp is much higher), could this also contribute to the discrepancy seen in the concentrations of secreted proteins? It may also enrich for specific populations of those cell types that express the tissue drivers highest. This may be another caveat that can be mentioned in the text.
c. Figure 2E: 100µm to 100µM?

Supplemental Figure 2 related
a. If specific proteins are endocytosed by a target cell and then secrete the protein again (apolpp being loaded with cargo for example), would this also be labeled? This is in regards to the unexpected tissue of origin being the muscle for apolpp in Supp Fig 2E.

Figure 3 related
a. Very interesting and exciting data. For those tissues showing overlap between other tissues in terms of secretome, could this be due to tissue driver non-specificity? For example, some glia subtypes, based on sequencing and some literature, also express apolpp. This could contribute to a shared secretome rather than independent events.
b. Minor for Figure 3E and Supplemental Figure 3D: It would be better to flip the tissue order in Supplemental Figure 3D as Figure 3E.

Figure 4 related. The overall snRNA-seq data can be further improved.
a. Fig 4B/C: it would be better to improve the color code of UMAP. Current color code is a bit confusing. Also, matching the labels of each cluster to Supplemental Figure 8 would be more helpful.
b. In Supplemental Figure 8, many genes show broad labeling although claimed as tissue specific. Did the authors try different tools for ambient RNA removal and doublet removal? Different combinations of these two steps? Sometimes it can be tricky to apply one single tool to the whole organism snRNA-seq dataset, which contains many different cell types.
c. Line 504: "In addition, using the glial marker gene repo, we identified 3,476 putative glial nuclei and computed a list of marker genes." There is no glial cluster formed from Fig 4B and 4C, or in the web data portal - https://www.flyrnai.org/scRNA/body_larvae/. 3,476 glia, if true, should form a big cluster. Also, cell types should be identified based on cluster level, not based on one gene's expression. Some repo ambient RNAs can be detected in other cell type cluster, but they are not glial cells.

Figure 5 related:
a. Fig 5E: The high false negative rate is a bit concerning. Would the high false negative rate from detecting the proteins in circulation also impact the TurboID detection of secreted proteins discussed previously? Did you observe any correlation

between the expression level of the secreted proteins (RNA) and the level of the protein obtained from the hemolymph?

Figure 6 related:
a. Did you also run FlyPhoneDB2 on the snRNA-seq data? It would be interesting to also validate how well the RNA and secretome analyses correlate through known interactions between cells. It would also provide a hint as to which interactions are most trustworthy (as not all predictions in the FlyPhoneDB2 analysis are accurate). Or can this secretome be used to update the FlyPhoneDB2?
b. The paper ends with Figure 6C summarizing only 6A and 6B. It will be nice to have an overall summary of all major findings from above sections to end the paper.

Minor things:
Line 99 and other places: single nuclear RNA-seq (snRNA-seq) should be single nucleus RNA-seq.
Line 40: change ‘remarkably’ to ‘remarkable’
Line 122: Would a brief description of the ER retention signal and secretion signal be necessary? Since other details about the system are mentioned.
Lines 153-158: The same information was repeated twice in this section. I think the sentence from lines 156-158 would be sufficient.
Line 186/189: Figure 1F is missing. 1G in Line 186 and 189 should be 1F? also there is no 1G in figure Legends.
Line 188: Spell out “TM”
Line 327: LC-MS/MS was already spelled out in Line 173. Can be removed from Line 327.
Lines 359-363: Based on the Western in Supp Fig 3B, it looks like there is a point where the washes may be too harsh (band in between 70 and 100 kDa). I may rephrase this to reflect that result. The band looks like it is gone with too high of Urea (maybe just difficult to see).
Line 495-496: it would be good to mention the total number of nuclei captured in the text, not only in the Figure.
Line 508: it is great to have the data available online. Maybe specifying the full link would be better https://www.flyrnai.org/scRNA/body_larvae/.
Acknowledgement: the last name of Tzu-Chiao is missing.

Version 1:

Reviewer comments:

Reviewer #1

(Remarks to the Author)
The authors have thoroughly adressed all the points raised by me and I appreciate the effort they made to improve the manuscript.
I believe this paper will be a great resource for everyone studying cell-specific secretome changes.

Reviewer #2

(Remarks to the Author)
I co-reviewed this manuscript with one of the reviewers who provided the listed reports. This is part of the Nature Communications initiative to facilitate training in peer review and to provide appropriate recognition for Early Career Researchers who co-review manuscripts.

Reviewer #4

(Remarks to the Author)
All my concerns have been sufficiently addressed.

Open Access This Peer Review File is licensed under a Creative Commons Attribution 4.0 International License, which permits use, sharing, adaptation, distribution and reproduction in any medium or format, as long as you give appropriate credit to the original author(s) and the source, provide a link to the Creative Commons license, and indicate if changes were made.
In cases where reviewers are anonymous, credit should be given to 'Anonymous Referee' and the source.
The images or other third party material in this Peer Review File are included in the article's Creative Commons license, unless indicated otherwise in a credit line to the material. If material is not included in the article's Creative Commons license and your intended use is not permitted by statutory regulation or exceeds the permitted use, you will need to obtain permission directly from the copyright holder.
To view a copy of this license, visit https://creativecommons.org/licenses/by/4.0/

Response to reviewer comments

We thank the reviewers for their helpful comments. Below we provide point-by-point responses to all comments. We also provide the changes to the manuscript text as tracked changes using Microsoft Word.

Please note that, in response to feedback from Reviewer #1, we reorganized the figures and text. As a result:
1) Most figure numbering changed
2) Some main Figures were moved to Supplementary Figures
3) Some main text was moved to Supplemental Notes. The Supplemental Notes show tracked changes.

We address all comments below referencing the revised figure/table/note numbers.

REVIEWER COMMENTS

Reviewer #1 (Remarks to the Author):

This manuscript addresses a timely and technically challenging question: the identification of cell- and tissue-specific secreted proteins and their roles in inter-organ communication. Secretome mapping remains limited by available methodologies, and the ability to trace tissue-specific origins of circulating proteins is of considerable diagnostic and therapeutic relevance.

The authors present an ambitious study that aims to map the tissue-of-origin of secreted proteins in Drosophila melanogaster larvae at an unprecedented scale. They employ TurboID proximity labeling, mass spectrometry, single-nucleus RNA sequencing (snRNA-seq), and CRISPR gene editing to generate an atlas of proteins present in the larval hemolymph and assign them to ten major tissue types. They identify 535 tissue-specific hemolymph proteins and observe a substantial correspondence between these results and whole-animal snRNA-seq datasets. The work is conceptually strong and leverages a simple and genetically tractable in vivo system to infer inter-organ communication pathways.

Major Comments
1. Balance of method development vs. biological insight
A substantial proportion of the manuscript is devoted to method optimization (TurboID expression strategies, ER labeling validation, optimization of bead pulldown conditions, etc.). While these steps are undoubtedly essential and the authors are well positioned as experts in proximity labeling, the emphasis on optimization detracts from the stated goal of generating a comprehensive tissue-specific secretome map.
These methodological details would be more appropriate as expanded Supplementary Information. Reducing their prominence in the main text would allow the biological insights—particularly the identification, classification, and functional interpretation of tissue-derived secreted proteins—to take center stage.

We thank the reviewer for this helpful point. In response, we have reorganized the text and figures to prioritize our quantitative tissue-secretome map as a new Figure 1 in the main text.

2. Validation and characterization of the genetic lines
The authors state that the ten tissue-specific TurboID lines exhibit normal phenotypes, but no supporting data are shown. Given that aberrant expression or ER-targeted enzymes could impact development or secretion pathways, phenotypic validation is essential for interpreting the secretome profiles. I recommend including data (e.g., viability, morphology, developmental timing) or citing established characterizations.

We thank the reviewer for raising this important point. We have added additional text in Supplementary Note 2 describing how these fly lines have been stably propagated for years, including at a public fly stock repository. Furthermore, we have added increased emphasis on cited phenotypic characterization of TurboID expression in flies and mice.

3. Unexpectedly low number of high-confidence secreted proteins
The authors identify 2,543 proteins by LC-MS/MS in total but conclude that only 535 represent tissue-specific secreted factors. This represents a modest fraction (~20%), which seems low given that a single cell line in vitro produced 72 secreted proteins, and that the global hemolymph proteome comprises over 4,500 proteins.
This raises questions about:
• The stringency of the filtering criteria
• Potential underestimation due to TurboID efficiency, labeling window, or tissue-specific expression differences
• The possibility that some true secreted proteins were excluded due to conservative thresholds

Response to reviewer comments

Clarifying the rationale and sensitivity/specificity tradeoffs in defining “high-confidence” secreted proteins would strengthen the manuscript. It may also be appropriate to discuss whether the 535 proteins represent a lower bound.

We thank the reviewer for this important point. We have added a new paragraph to the discussion that addresses this point about filtering stringency and the possibility of true secreted proteins excluded due to conservative thresholds. In addition, we added discussion about TurboID efficiency, labeling window, or tissue-specific expression differences, which also addresses a similar concern from Reviewer #4.

What is captured in their detection method?
Are small polar proteins among the peptides detected in the MS/MS?
Can such proteins be validated to be present by the authors detection method by plotting the amount of small polar proteins present in the Drosophila proteome as a distribution plot against what is found in the MS/MS analysis of both the full proteome and the proteins expressed from specific tissues (TurboID tagged and biotin purified) ? A pie diagram would suffice.

As described in the methods, we used standard reverse phase liquid chromatography with a C18 stationary phase that separates peptides on hydrophobicity. We added text to the methods that clarify that, for the tissue secretome map, the instrument was set for acquisition using positive ion mode. It is likely that there are biases for identification of small polar proteins because (1) smaller proteins produce less tryptic peptides that can be identified by LC-MS/MS (e.g. PMID 37223358), (2) highly polar and hydrophilic peptides elute earlier and possibly in the void volume, therefore may not be well-separated by the LC gradient leading to poor identification, and (3) highly polar peptides may result in poor fragmentation patterns for MS/MS identification.

We now address potential bias against small proteins in the results and discussion. We also present new analysis (Supplementary Figure 10, Supplementary Table 3E) on the proportion of small proteins ≤100 amino acids. This analysis showed that small proteins are present in both our whole hemolymph and tissue secretome datasets. Compared to the fraction of small proteins in the fly proteome (6.0%), whole hemolymph and tissue secretome datasets have smaller fractions (2.2% and 1.9%, respectively). This difference may reflect the LC-MS/MS underestimates described above, or because less small proteins are secreted than intracellular. The slight bias against identifying small proteins in tissue secretome datasets relative to whole hemolymph (1.9% vs 2.2%) may reflect additional bias introduced by streptavidin-based enrichment with on-bead digestion, as described by others (PMID: 39231529). For example, biotinylated peptides typically remain bound to streptavidin after on-bead digestion. For small proteins, there are few remaining peptides left for protein identification by LC-MS/MS.

Following my initial comment and regarding the number of proteins the authors detect (535 proteins) compared to the total amount of proteins in the plasma they detect (>4500 proteins): It is my understanding that the TMT - MS/MS analysis is done in a way so that any proteins that are not expressed in all the specific tissues are excluded, which if true is producing a lot of 'false negative' proteins. It is not clear in the manuscript if the zero values are excluded from the TurboID labelled samples. Please mention is the values are imputed or not. Zero values are difficult to deal with, but this should maybe be commented on as a limitation.

For the TMT analysis, each tissue group was analyzed separately, and we did not require the protein to be detected across all other tissue groups or plexes. We did not impute missing/zero values, however we had filtering criteria to deal with missing values. As a reminder, each plex has 2 tissue groups (Figure 3a). Within each plex, if the negative control (non-TurboID experimental group) had missing values in all replicates, the protein was excluded from analysis in that plex (for both tissue groups) because we cannot calculate a fold-change. Similarly, if the experimental group in the plex has missing values in all replicates, then that protein was excluded only from that tissue group, as fold-changes cannot be estimated. By not imputing, we are following a conservative approach to provide a high quality list of proteins with minimal false discoveries, however, as noted in the response to the reviewer’s comment above, we understand that this conservative approach has tradeoffs which we explain in the Discussion. We have added text to the Methods clarifying how missing values are handled during data analysis. Furthermore, to highlight possible “false negative” proteins that were excluded by our criteria, we provide a list of “infinite” log2fold change proteins (i.e. proteins identified in all three tissue replicates and never identified in any negative control replicates) in Supplemental Table 3C. A description of this list is added in the Results and Methods sections.

4. Limited functional analysis of the identified secreted proteins
Many of the 535 proteins lack prior annotations as secreted factors, which highlights the novelty of the dataset. However, the manuscript devotes relatively little space to functional interpretation, GO/pathway analysis, evolutionary conservation, or connections to known signaling pathways. This is a missed opportunity for deeper biological insight.

Response to reviewer comments

Greater emphasis on:
• Functional categories within tissue-specific secretomes
• Enrichment analyses (and their biological implications)
• Homology to human proteins and translational relevance
would significantly increase the impact of the study.

We thank the reviewer for this helpful suggestion. In response, we have expanded our prior bioinformatic functional analysis of the 535 tissue-secreted proteins as a stand-alone functional bioinformatic analysis Results section, a new paragraph in the Discussion, a new Main figure, and supplemental figures/tables (Figure 2, Supplementary Figures 14-16, Supplementary Table 4)

Specifically, we added gene set enrichment analysis using PANGEA, to expand on our prior g:Profiler analysis. Together, this work reinforces that extracellular function terms are highly enriched in our dataset, and that there are tissue-specific pathways enriched across our dataset.

We also expanded on our prior human homology searches. We added the number of fly proteins with at least one human homolog with an OMIM entry, as well as enrichment of human disease associations using PANGEA. These new analyses reinforce the value and human translational relevance of our fly dataset.

We also expanded the discussion to highlight ectodomain shedding as a potential mechanism for generating circulating inter-organ factors, since we identified a number of transmembrane proteins in our hemolymph tissue-secretome, and because we identified the transmembrane protein CG6867 ectodomain as transiting from muscle to imaginal discs.

5. Discrepancy in GFP-knock-in validation
When the authors perform GFP knock-ins to visualize selected tissue-specific secreted proteins, only a small subset is detectable in the hemolymph. This discrepancy warrants more discussion. Does GFP tagging interfere with secretion, folding, or stability? Are expression levels too low at the larval stage?
Addressing this point is important for validating the platform and for interpreting the absence of GFP signal as biological rather than technical.

We thank the reviewer for these important comments. We have added a paragraph to the discussion addressing these concerns, as well as a new comparison of gene expression vs protein band detection in the Results and Supplemental Table 7C. See also the response to a similar concern from Reviewer #4.

6. Limited demonstration of inter-organ communication
One of the stated aims is to identify inter-organ communication factors. However, only two proteins are ultimately highlighted as examples. While the resource remains valuable, the biological conclusions regarding communication networks appear modest. This should be more clearly acknowledged in the Discussion.

We thank the reviewer for their comments. Respectfully, the two inter-organ factors highlighted in our manuscript were identified through extensive molecular, genetic, and imaging work, reflecting the high bar required to validate bona fide inter-organ trafficking in vivo.

In response to the reviewer's concern, we added bioinformatic analysis of our dataset to highlight both known and predicted inter-organ proteins. We also expanded the text to better contextualize the scale of our experimental efforts to identify candidate inter-organ proteins, including text in the results, discussion, and Supplemental Figure 19 describing improved CRISPR-HDR donor plasmids that facilitated generating the knock-in fly lines.

Specifically, focusing on the 535 tissue-secreted proteins, we identified 33 known interorgan proteins and a larger set of 143 interorgan candidate proteins. We also point out examples of inter-organ proteins that were confirmed secreted from the expected tissue, as well as examples of unexpected tissues. These additions are presented as new Results and Discussion text and supported by new Figures and Supplements (Figure 2E, Supplemental Table 3B). This new information complements our existing experimental results, and we feel it is a valuable contribution to the manuscript and as a community resource.

In addition, in response to Reviewer #4, we added FlyPhoneDB2 analysis of our snRNA-seq data, which revealed additional candidate inter-organ ligand-receptor pairs (Supplementary Figure 18, Supplementary Table 6K).

Response to reviewer comments

7. Developmental-stage limitation
The authors use Drosophila larvae, which may limit the relevance of the secretome to adult physiology and known inter-organ axes (e.g., fat body–muscle, intestine–brain). This developmental constraint should be addressed more explicitly, especially in relation to claims of comprehensiveness and translational relevance.

We thank the reviewer for raising this point. We have now added text to the Discussion noting that our focus on 3rd instar larvae may limit extrapolation to adult fly physiology and translation. As already described in the manuscript, larvae were intentionally chosen because this developmental stage uniquely enables collection of sufficient hemolymph for large-scale streptavidin pulldown and replicated quantitative mass spectrometry. We therefore view the larval secretome as a foundational resource, with the expectation that analogous approaches could be adapted in the future to other developmental stages as technical limitations permit.

Minor Comments
1. Clarify S2R+ cells.
Please provide a brief description or reference for Drosophila S2R+ cells, as not all readers may be familiar with their properties and suitability for ER-TurboID validation.

We thank the reviewer and have added additional text to Supplementary Note 1 to clarify this point.

2. Figure 1 ER-localization validation.
Orthogonal confocal projections would help demonstrate ER specificity more convincingly.

We thank the reviewer for this comment. We have added an increased zoom image of TurboID-ER, as well as 10 examples of transfected cells for GFP-TurboID and for GFP-TurboID-ER (Supplementary Figure 1). These results show GFP-TurboID localization in cytoplasm and nuclei, whereas GFP-TurboID-ER colocalizes with the ER marker (Cnx99A). We hope this satisfies the reviewers comment without the need for additional confocal analysis. We also note that our results match ER co-localization results using Myc-BirA*G3-ER in Supplemental Figure 2 and as seen by other groups (Droujinine et al. 2021, PMID: 33888706; Yang et al., 2022, PMID: 35946312)

3. Negative control conditions.
A negative control using flies raised without supplemental biotin would help quantify background biotinylation.

We thank the reviewer for this suggestion. Respectfully, we consider this proposed experiment as peripheral to our goal of generating a tissue secretome map.

First, this type of experiment has been documented by others. For example, Branon et al. 2018 (PMID: 30125270) showed that TurboID in flies can utilize endogenous biotin in fly food to achieve low-level labeling, biotin supplementation does not substantially increase the abundance of naturally biotinylated proteins, and biotin supplementation drives robust labeling. Similar observations have been reported for BirA*G3-based labeling in mice (Yang et al., 2022, PMID: 35946312). Therefore, a "ligase+ biotin-" condition would not represent a clean negative control and may underestimate background labeling.

Second, we deliberately chose our negative control (UAS-GFP-TurboID-ER with no Gal4 driver) with supplemental biotin for several reasons. 1) To simultaneously control for multiple types of background signal - endogenously biotinylated proteins (e.g., ACC), non-specific streptavidin binding, and possible Gal4-independent TurboID activity. 2) As a universal control for all the tissue-Gal4 UAS-GFP-TurboID-ER experiments, as opposed to biotin-counterparts for every X-Gal4 UAS-GFP-TurboID-ER line. 3) To ensure labeling experiments are consistent and reproducible, as fly food batches may vary in the amount of endogenous biotin. 4) Using such a "ligase- biotin+" negative control is typical when performing proximity labeling with large-scale pulldown and MS from other groups (Sears et al. 2020, PMID: 31161514; Dumrongprechachan et al. 2021, PMID: 34381044; Yang et al. 2022, PMID: 35946312; Droujinine et al. 2021, PMID: 33888706). Therefore, we believe our control provides a more appropriate and conservative baseline for defining ligase-dependent enrichment in our MS-based analyses.

We have added text to Supplementary Note 2 clarifying our rationale for using a ligase-, biotin+ negative control rather than a biotin- condition.

4. Supplementary Fig. 7 – GO analysis.
The biological relevance of the top two pathways should be explained, particularly in relation to secretome function.

We thank the reviewer. We believe we have addressed this comment in the course of responding to major comment #4, please see above.

5. Functional analysis.
Additional description or discussion of functional enrichment results would be beneficial.

We thank the reviewer. We believe we have addressed this comment in the course of responding to major comment #4, please see above.

Assessment of Data, Methods, and Presentation
The methodology is generally sound and employs state-of-the-art tools for proximity labeling and proteomics. The mass spectrometry data appear of high quality, and the integration with snRNA-seq is a strong asset. Figures are clearly presented, although some could be reorganized to emphasize biological results over methodological detail. I do not identify concerns about statistical validity, but the manuscript would benefit from clearer justification of filtering criteria and explicit discussion of detection thresholds. All referenced datasets appear appropriate, though I encourage the authors to ensure full availability of raw and processed data for community use.

Significance
This work represents a substantial technical effort toward mapping tissue-specific secretomes in vivo. While the resource is valuable and the methodological platform may be widely useful, the biological conclusions are relatively limited, and the secretome map is incomplete both in developmental scope and functional interpretation. Nevertheless, the study constitutes an important step in enabling scalable, genetically tractable secretome profiling and may serve as a basis for future work in both flies and higher organisms.

Reviewer #2 (Remarks to the Author):

I co-reviewed this manuscript with one of the reviewers who provided the listed reports. This is part of the Nature Communications initiative to facilitate training in peer review and to provide appropriate recognition for Early Career Researchers who co-review manuscripts.

Reviewer #4 (Remarks to the Author):

This manuscript by Bosch et al employed TurboID-based proximity labeling to characterize the secretomes in Drosophila third instar larvae. They identified identified 535 circulating secreted proteins from the larval blood. Then they performed snRNA-seq of the whole larvae to validate these proteins' origin tissues. They also generated knock-in fly lines using CRISPR and revealed proteins that are synthesized in one tissue and localized to another.

Overall, this interesting and comprehensive study establishes a novel platform for secretome discovery in Drosophila. It provides very useful resources to study secreting proteins from larval stages and also opens the possibility to systematic characterize adult secretomes for inter-organ communication. I don't have major concerns, but have several suggestions to further improve this manuscript.

General comments:
Figure 1 related
a. Could the authors explain why more proteins were detected from the MT-GFP-TurboID than MT-GFP-TurboID-ER cell media in Fig 1F?

We thank the reviewer for this intriguing point.

Cell/tissue leakage is a well-known and inherent problem with proteomics of plasma and culture media. For example, human plasma contains intracellular proteins (PMID: 31772123), and our Drosophila raw plasma MS dataset (non-pulldown) does as well (e.g. Actin and Tubulin). In cultured cells, we attempted to minimize this effect by gentle manipulation, described in the Methods.

Response to reviewer comments

Furthermore, unconventional secretion including release of extracellular vesicles is an established mechanism of secretion. Extracellular vesicles are typically 30–150 nm in diameter (PMID: 31311206), which is smaller than the 0.2μm filter used to filter our culture media, and so should be present in our purified cell media samples.

As to why more proteins were detected from MT-GFP-TurboID samples, this may be due to one or more factors. For example 1) Cytoplasmic proteins may be more abundant than secreted proteins (e.g. 22728825), 2) GFP-TurboID may have higher enzymatic activity in the cytosol (supported by Branon et al. PMID: 30125270), and/or 2) the MT-GFP-TurboID cell line may express higher levels of GFP-TurboID (Supplementary Figure 3A shows GFP-TurboID levels are higher than GFP-TurboID-ER). We added this explanation in Supplementary Note 1.

b. Since many proteins localize to the ER (primary site for translation), is it possible that many of the unpredicted secreted proteins in the GFP-TurboID-ER are non-specific biotinylation? Or could this have been from the gel lane extraction? Maybe elaborating on this result in the text would be helpful. It may also be possible that secreted proteins could be rapidly taken up by the cell that just secreted them. Is there sufficient time for this to occur?

We interpret the reviewer's comment to mean that "non-specific biotinylation" refers to proteins that were labeled by TurboID outside the ER lumen, such as those near the cytoplasmic face of the ER. Apologies if this was misinterpreted. ER-localized TurboID should, in theory, not label nuclear and cytoplasmic proteins, but still, some are present in our secretome datasets, such as ribosomal proteins. We agree that one explanation is if some proportion of GFP-TurboID-ER were to mislocalize to the cytoplasm, it could biotinylate cytoplasmic proteins, and these labeled proteins could leak out of cells or be released in exosomes. We already address this explanation in the discussion, but have added additional text to the discussion to make it clear this scenario could occur in cell culture and in vivo. Interestingly, all studies using promiscuous biotin ligases (BioID/BirA*G3/TurboID) to identify classically secreted proteins (e.g. Droujinine et al. 2021, Nat. Commun., PMID: 33888706) identify some nuclear/cytoplasmic proteins, implying this is an inherent problem with this approach.

We do not believe the problem occurred from problems with gel lane extraction as the lanes were spaced 1 lane apart, and because we observe similar results using in vivo GFP-TurboID-ER, including experiments in which we used on-bead digestion as opposed to gel digestion.

We interpret the final comment about secreted proteins taken up by cells to be related to the comment by the same reviewer above ("Supplemental Figure 2 related"), concerning endocytosis of extracellular proteins, which are then labeled by recipient cells expressing GFP-TurboID-ER, and then re-secreted. Apologies if this was misinterpreted. We have added text to the discussion to address this point. As for whether there is sufficient time for this to occur, this is an interesting question. Estimates for endocytosis and re-secretion are in the 1-20min range (e.g. PMID: 31843759). Considering labeling occurs S2R+ cells for 24 hours, this indicates that there is sufficient time for endocytosis, labeling, and re-secretion to occur in our experiments.

Figure 2 related
a. Fig 2G: 213 proteins are detected from tissue specific labeling sample with streptavidin pulldown, and 185 proteins were detected from wild type control without pulldown. How many of these two groups are overlapped? Also, an important control can be from wild type with streptavidin pulldown. This control will help validate the fidelity of tissue-labeling and to identify background noise. It seems the authors have this data (line 313/314) but didn't compare this control with these 213 and 185 proteins. Additional analysis will be appreciated.

We observed an overlap of 56 proteins between the 213 proteins from tissue labeling samples and 185 proteins from wild-type non-pulldown samples. This information is now reflected in Supplementary Note 3, a Venn diagram in Supplementary Figure 7B, and the protein IDs are in Supplemental Table 2B.

We regret that the data concerning the negative control was not clear and have taken steps to better highlight this and perform the requested additional analysis. We added new text Supplementary Note 3, updated Supplementary Figure 7B, added new Supplemental Figures 7G-H, and new Supplemental Table 2C. This showed that all proteins identified from negative control pulldowns were identified in at least one of the tissue-labeling samples, illustrating the extent of background noise. Furthermore, ACC and Pcb were identified with pulldown but not in non-pulldown samples, suggesting these naturally biotinylated proteins are present in the collected hemolymph and enriched with streptavidin pulldown. We note that our quantitative tissue-secretome proteomic map extensively uses such negative controls to normalize tissue-labeling results, and we consider the data collected in Supplementary Figure 6-7 as proof of principle and rationale for why we optimized bead washing conditions to reduce background binding.

b. Since different tissue drivers likely have different expression levels (based on snRNA-seq, repo is lower whereas apolpp is much higher), could this also contribute to the discrepancy seen in the concentrations of secreted proteins? It may also enrich for specific populations of those cell types that express the tissue drivers highest. This may be another caveat that can be mentioned in the text.

We thank the reviewer and have added additional text to the discussion to address this caveat.

c. Figure 2E: 100μm to 100μM?

We thank the reviewer and have made this change.

Supplemental Figure 2 related
a. If specific proteins are endocytosed by a target cell and then secrete the protein again (apolpp being loaded with cargo for example), would this also be labeled? This is in regards to the unexpected tissue of origin being the muscle for apolpp in Supp Fig 2E.

We thank the reviewer for this comment. We have added text to the discussion to address this point.

Figure 3 related
a. Very interesting and exciting data. For those tissues showing overlap between other tissues in terms of secretome, could this be due to tissue driver non-specificity? For example, some glia subtypes, based on sequencing and some literature, also express apolpp. This could contribute to a shared secretome rather than independent events.

We thank the reviewer for their excitement and appreciate this helpful comment. We agree that overlap between tissue secretomes (i.e. proteins that are secreted from >1 tissue), could in fact be due to unexpected expression of our Gal4 driver outside of the expected domain. We have added text and references to the Discussion to address this important caveat.

b. Minor for Figure 3E and Supplemental Figure 3D: It would be better to flip the tissue order in Supplemental Figure 3D as Figure 3E.

We thank the reviewer and have made this change.

Figure 4 related. The overall snRNA-seq data can be further improved.
a. Fig 4B/C: it would be better to improve the color code of UMAP. Current color code is a bit confusing. Also, matching the labels of each cluster to Supplemental Figure 8 would be more helpful.

We thank the reviewer for this helpful suggestion. Because the snRNA-seq dataset contains 28 annotated clusters, it is challenging to assign a distinct and easily distinguishable color to each cluster within a single UMAP visualization. To address this, the associated data portal https://www.flyrnai.org/tools/rna_seq_base/web/showProject/59/plot_coord=1/sample_id=all) allows users to interactively explore the UMAP; hovering over individual cells displays the corresponding cell-type annotation. In addition, Figure 3B-C now includes cluster number annotations, enabling readers to more easily cross-reference clusters with those shown in Supplemental Figure 17 and the associated supplemental tables.

b. In Supplemental Figure 8, many genes show broad labeling although claimed as tissue specific. Did the authors try different tools for ambient RNA removal and doublet removal? Different combinations of these two steps? Sometimes it can be tricky to apply one single tool to the whole organism snRNA-seq dataset, which contains many different cell types.

We thank the reviewer for raising this point and for highlighting a common challenge in whole-organism snRNA-seq analyses. We did evaluate multiple approaches for ambient RNA correction and doublet detection, including methods such as Dropkick and DecontX. After careful comparison of these strategies, we selected SoupX and DoubletFinder as the most appropriate combination for this dataset. Specifically, all samples were first corrected for ambient RNA using SoupX with default parameters, and the corrected expression matrices were subsequently processed with DoubletFinder to identify and remove doublets, using an estimated doublet rate of 4.6%.

c. Line 504: “In addition, using the glial marker gene repo, we identified 3,476 putative glial nuclei and computed a list

of marker genes." There is no glial cluster formed from Fig 4B and 4C, or in the web data portal - https://www.flyrnai.org/scRNA/body_larvae/. 3,476 glia, if true, should form a big cluster. Also, cell types should be identified based on cluster level, not based on one gene's expression. Some repo ambient RNAs can be detected in other cell type cluster, but they are not glial cells.

We agree that, under typical circumstances, glial cells would be expected to form a distinct cluster and that cluster-level annotation is generally preferable to assignment based on a single marker gene. In this larval whole-organism dataset, however, we found that standard clustering approaches were unable to reliably separate glial nuclei from other cell types, even after applying ambient RNA correction and doublet removal. Notably, this challenge appears specific to the larval dataset and contrasts with our experience analyzing comparable adult fly snRNA-seq datasets, in which glial clusters are readily resolved.

Figure 5 related:
a. Fig 5E: The high false negative rate is a bit concerning.

We agree that the high false negative rate in this experiment does not fit our expectations and was disappointing considering the investment of time and resources to construct the GFP knock-in fly lines. We have added a paragraph in the discussion to address this point. See also similar concern from Reviewer #1.

Would the high false negative rate from detecting the proteins in circulation also impact the TurboID detection of secreted proteins discussed previously?

In theory, many variables can contribute to false negatives in our anti-GFP western blots. 1) Low abundance of the protein in the hemolymph, 2) instability/degradation due to the GFP tag, 3) cleavage of the protein such that the GFP-tagged portion is not secreted or degraded, 4) high background signal from the anti-GFP antibody, 5) overlap of faint GFP-tagged protein band and background band at same molecular weight, obscuring the GFP-tagged protein band.

#1 would impact the detection of TurboID-labeled biotinylated proteins by pulldown/MS, which we previously mentioned in the discussion. These experiments are not directly comparable, however, because pulldown enriches proteins and decomplexifies the sample (non-biotinylated proteins are washed away).

Did you observe any correlation between the expression level of the secreted proteins (RNA) and the level of the protein obtained from the hemolymph?

We thank the reviewer for this helpful suggestion. Using DGET, we analyzed RNA-seq expression of the 19 experimental genes shown in Figure 4E, and 5 positive control genes shown in Supplemental Figure 21 (24 genes total). In some cases, RNA-seq levels clearly matched protein detection by western blot, such as high apolpp transcript abundance and a robust Apolpp-GFP band (Supplemental Figure 21). To test whether missing GFP bands were due to low gene expression, we compared RNA-seq expression levels between genes with detectable GFP bands ($n = 10$) and those without detectable bands ($n = 14$) using Mann-Whitney U tests, performed independently across six larval developmental stages (L1 through puffstage 7–9). At no developmental stage did genes with detectable GFP bands show significantly higher expression than genes without detectable bands (all $p > 0.48$). This indicates that lack of GFP-tagged protein detection cannot be explained simply by lower gene expression. We provide this analysis in Supplemental Table 7, text in the results and methods sections.

We note that the false negative rate appears similar for both experimentals (Fig. 4E) and positive controls (Supplemental Figure 21) GFP-tagged fly lines, which we previously pointed out in the results. 2 out of 5 lines (40%) showed GFP-tagged protein band by western, and 8/19 (42%) lines for experimental samples. Therefore, we believe that false-positives are inherent in this protocol.

To our knowledge, no one has systematically compared hemolymph protein abundance by western blot with the gene expression of the encoding gene. We hope this information will be valuable to the community, and stimulate efforts to detect hemolymph proteins with higher signal-to-background, such as with optimized anti-GFP antibodies, blotting conditions, or alternative methods (e.g. https://www.biorxiv.org/content/10.1101/2025.10.31.685841v1.full)

Figure 6 related:
a. Did you also run FlyPhoneDB2 on the snRNA-seq data? It would be interesting to also validate how well the RNA and secretome analyses correlate through known interactions between cells. It would also provide a hint as to which

interactions are most trustworthy (as not all predictions in the FlyPhoneDB2 analysis are accurate). Or can this secretome be used to update the FlyPhoneDB2?

We thank the reviewer for their helpful suggestion. In response, we have added FlyPhoneDB2 analysis of our snRNA-seq data, with a new paragraph in the text, supplemental figure, and supplemental table (Supplementary Figure 18, Supplementary Table 6K). These results show known and potentially novel ligand-receptor relationships between cell-types and organs.

b. The paper ends with Figure 6C summarizing only 6A and 6B. It will be nice to have an overall summary of all major findings from above sections to end the paper.

We thank the reviewer for this thoughtful suggestion. In response, we have added a new concluding figure (Figure 6) that summarizes the main strategies and findings from the paper as a schematic (similar to a graphical abstract). The previous inter-organ trafficking schematic was removed from Figure 5, which now only contains confocal data.

Minor things:
Line 99 and other places: single nuclear RNA-seq (snRNA-seq) should be single nucleus RNA-seq.

We thank the reviewer and have made this change.

Line 40: change ‘remarkably’ to ‘remarkable’

We thank the reviewer and opted to delete the word “remarkably”

Line 122: Would a brief description of the ER retention signal and secretion signal be necessary? Since other details about the system are mentioned.

We thank the reviewer and have added this description in Supplementary Note 1.

Lines 153-158: The same information was repeated twice in this section. I think the sentence from lines 156-158 would be sufficient.

We apologize for the confusion. The first sentence addressed biotinylated proteins in the media. The 2nd sentence addressed GFP-TurboID protein in the media. We have rewritten these sentences in Supplementary Note 1 with improved clarity.

Line 186/189: Figure 1F is missing. 1G in Line 186 and 189 should be 1F? also there is no 1G in figure Legends.

Good catch, thank you. We have fixed this problem in the process of restructuring the figures.

Line 188: Spell out “TM”

We thank the reviewer and have made this change.

Line 327: LC-MS/MS was already spelled out in Line 173. Can be removed from Line 327.

We thank the reviewer and have made this change.

Lines 359-363: Based on the Western in Supp Fig 3B, it looks like there is a point where the washes may be too harsh (band in between 70 and 100 kDa). I may rephrase this to reflect that result. The band looks like it is gone with too high of Urea (maybe just difficult to see).

We respectfully disagree. In Supplementary Figure 8B, the major band between 70kDa and 100kDa, which we labeled as “LSPs” and assume is biotinylated LSP-1, remains constant intensity with higher concentrations of Urea in the wash. The reviewer may be referring to Supplementary Figure 8A, which shows that endogenous LSPs between 70kDa and 100kDa non-specifically bind streptavidin beads, and this non-specific binding is greatly reduced by increasing Urea concentrations (as well as SDS). We acknowledge that the difference between the experimental details between Supplementary Figure 8A and B are subtle. To clarify this, we added experimental schematics to Supplementary Figure 8.

Response to reviewer comments

Line 495-496: it would be good to mention the total number of nuclei captured in the text, not only in the Figure.

We thank the reviewer and have added the total of number filtered nuclei to the results text.

Line 508: it is great to have the data available online. Maybe specifying the full link would be better https://www.flyrnai.org/scRNA/body_larvae/.

We thank the reviewer. The full link was previously only in the methods section. Now we added the full link to the results section.

Acknowledgement: the last name of Tzu-Chiao is missing.

We thank the reviewer and have added Tzu-Chiao’s last name.
